# Fluorous-Directed Assembly of DNA Origami Nanostructures

**DOI:** 10.1021/acsnano.2c10727

**Published:** 2022-12-20

**Authors:** Jiajia Zou, Ashley C. Stammers, Andrea Taladriz-Sender, Jamie M. Withers, Iain Christie, Marina Santana Vega, Badri L. Aekbote, William J. Peveler, David A. Rusling, Glenn A. Burley, Alasdair W. Clark

**Affiliations:** † James Watt School of Engineering, Advanced Research Centre, University of Glasgow, Glasgow G11 6EW, United Kingdom; ‡ Department of Pure Applied Chemistry, Thomas Graham Building, 295 Cathedral Street, University of Strathclyde, Glasgow G1 1XL, United Kingdom; § School of Chemistry, Joseph Black Building, University of Glasgow, Glasgow G12 8QQ, United Kingdom; ∥ School of Pharmacy and Biomedical Sciences, St. Michael’s Building, University of Portsmouth, Portsmouth PO1 2DT, United Kingdom

**Keywords:** DNA origami, DNA nanotechnology, self-assembly, fluorous, fluorous DNA, molecular recognition, DNA origami dimerization

## Abstract

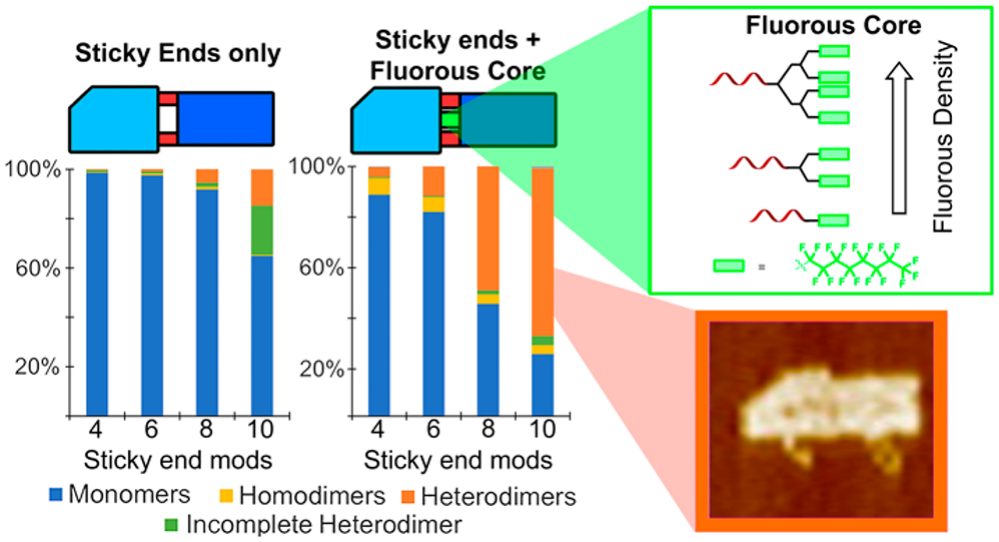

An orthogonal, noncovalent approach to direct the assembly of higher-order DNA origami nanostructures is described. By incorporating perfluorinated tags into the edges of DNA origami tiles we control their hierarchical assembly via fluorous-directed recognition. When we combine this approach with Watson–Crick base-pairing we form discrete dimeric constructs in significantly higher yield (8x) than when either molecular recognition method is used in isolation. This integrated “catch-and-latch” approach, which combines the strength and mobility of the fluorous effect with the specificity of base-pairing, provides an additional toolset for DNA nanotechnology, one that enables increased assembly efficiency while requiring significantly fewer DNA sequences. As a result, our integration of fluorous-directed assembly into origami systems represents a cheap, atom-efficient means to produce discrete superstructures.

## Introduction

DNA self-assembly is the pre-eminent strategy to construct multidimensional functional nanostructures with angstrom-level precision.^1^ The DNA origami method uses Watson–Crick base-pairing to direct the folding of a long single-stranded piece of DNA (ssDNA) (the scaffold) with a set of complementary oligodeoxyribonucleotide (ODN) strands (staples), whose rational design leads to the creation of intricate two- and three-dimensional nanostructures.^2−5^ The modularity of DNA origami and the accessibility of a defined ssDNA template makes this approach one of the most versatile methods to predictably and reproducibly prepare nanostructures in the 100 nm regime. Furthermore, the incorporation of recognition elements in ODN staples permits the spatial arrangement of components with subnanometer precision, providing additional functionality for applications spanning biosensing, drug delivery, and the fabrication of photonic devices.^6−9^

**Figure 1:**
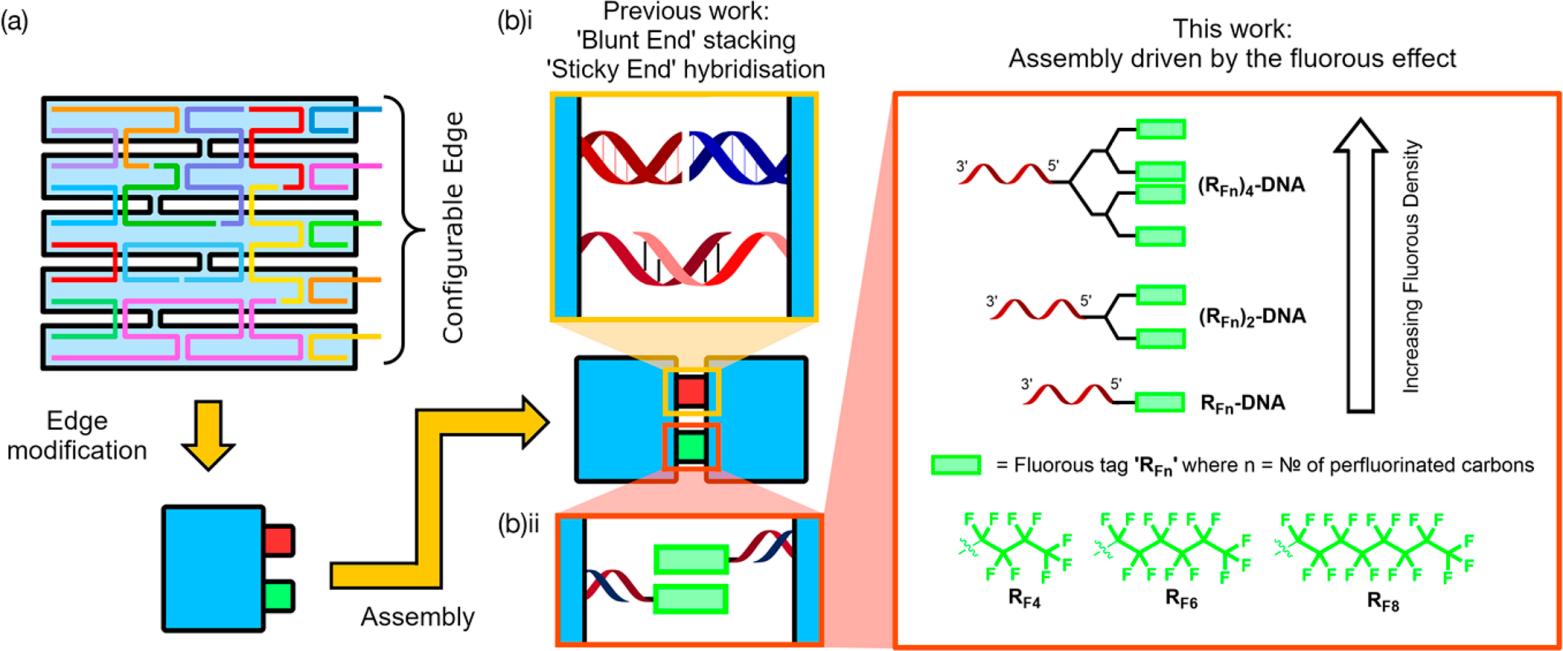
Schematics showing (a) a typical origami structure with configurable edge modifications, folded into shape using a circular DNA scaffold (black line) and DNA staples (colored lines). (b) Assembly of DNA origami dimers using edge modifications: (i) previously known DNA-based methods; (ii) the fluorous effect as a tool for hierarchical origami assembly.

As the field of DNA nanotechnology progresses from the design of nanostructures toward their application, one current limitation of DNA origami as an engineering tool is the length of the scaffold strand, for which the circular 7249 nucleotide (nt) M13mp18 phage DNA is the most commonly used, confining the dimensions of folded origami structures to ∼100 nm. To overcome this limitation, a methodology is now required to enable the assembly of origami nanostructures into discrete micron-scale ensembles. Although increasing the length of the scaffold strand is one strategy, this method requires an increase in the number of staples required to fold the resultant structure, complicating the design and increasing the cost of singular origami motifs.^10,11^ An alternative strategy to address these size limitations is the hierarchical assembly of individual DNA origami nanostructures into higher-order superstructures (Figure 1a).^12−14^

Incorporating ssDNA overhangs (sticky ends) onto the edges of nanostructures that base-pair with complementary sequences or the use of blunt-ended base-stacking interactions are the most common of these strategies, and they have been used to great effect to promote the assembly of extended two-dimensional assemblies and complex, three-dimensional origami superstructures.^15−18^ Theoretically, these highly programmable pure DNA approaches should enable architecture scaling to any desired size; however, in practice they have distinct limitations. Blunt end stacking is restricted by the number of helical edges within an origami structure and requires stringent alignment of these edges, making it sensitive to global origami distortions. Both sticky- and blunt-ends methods have the disadvantage that the binding strength of individual recognition elements is sequence-dependent, coming with the associated cost of large sets of unique DNA sequences. They are further hampered by the low yield of final products, which drops precipitously as the assembly sizes increase (for structures approaching 1 μm in size yields can be as low as 2–3%).^19^

Blending orthogonal modes of molecular recognition that can function co-operatively^20−22^ has the potential to address the limitations of these pure DNA methods and represents a step toward creating a library of nucleic acid recognition elements analogous to the more diverse range of elements employed in protein–protein assembly.^23^ The incorporation of perfluorinated tags at precise locations within a DNA sequence is one such molecular recognition modality. These fluorine-rich groups preferentially associate with one another while excluding other forms of hydrophilic and hydrophobic interactions, a phenomenon known as the “fluorous effect”.^24^ The fluorous effect has been utilized extensively in the preparation of microarrays^25−28^ in which small molecules or biomacromolecules incorporating perfluorinated tags [-(CF_2_)_*n*_CF_3_ where *n* ≥ 3] are immobilized onto fluorous-micropatterned surfaces. The affinity of self-association of perfluorinated tags (R_F_) is far stronger than equivalent hydrophobic interactions, rendering the fluorous effect an atom-efficient yet reversible noncovalent interaction.

We surmised that the strategic placement of perfluorinated tags at the interface between origami nanostructures would provide an orthogonal recognition mode for their hierarchical assembly; a strong yet mobile binding solution (the fluorous pony-tails can slide over one another without separating) to deliver added stability when mixed with ssDNA sticky ends. In this paper, we show that blending DNA base-pairing with fluorous-directed recognition results in a “catch-and-latch” system; the fluorous effect provides strength and stability, while the DNA provides specificity, locking the individual origami into the correct positions (Figure 1). The result is an assembly methodology for origami dimers, which, in our model system, results in a significantly higher yield than can be achieved using DNA alone.

## Results and Discussion

**Figure 2:**
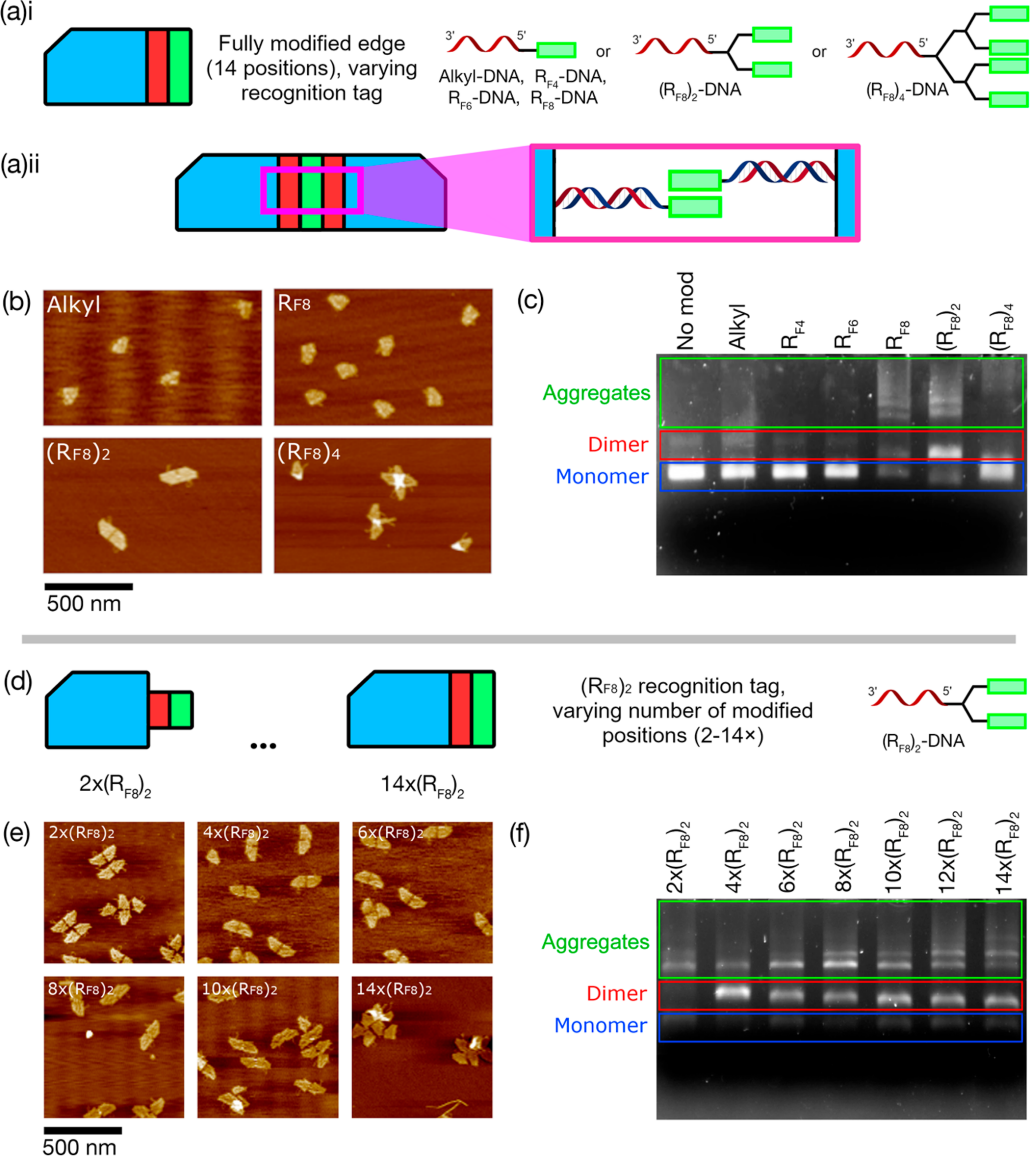
**(a)i**. Schematic illustration of an origami tile modified at 14 positions along one edge using either an alkyl (hydrophobic, nonfluorous control), R_F4_, R_F6_, R_F8_, (R_F8_)_2_, or (R_F8_)_4_ tag. **(a)ii**. Schematic showing dimerization via fluorous-modified DNA hybridized to the edge staples of the origami. (**b**) AFM images and (**c**) AGE images showing origami assemblies resulting from (a). (**d**) Schematic illustration of origami tiles modified at 2–14 postions along one edge using (R_F8_)_2_ tags. (**e**) AFM images and (**f**) AGE images showing origami assemblies resulting from (d).

The origami structure shown in Figure 2 was used as our workhorse design; it is a truncated rectangle with dimensions of 70 × 90 nm, where staples along the short edge were modified to include molecular recognition groups. Perfluorinated tags were incorporated into the structure by hybridizing ODNs containing fluorous tags (R_F_-ODNs) to sticky ends of an identical sequence, providing a flexible system in which the tile itself is assembled first and is later modified by incubation with the appropriate R_F_-ODN, allowing for a maximum of 14 tags. Assessment of the origami assembly was done using a combination of agarose gel electrophoresis (AGE) and atomic force microscopy (AFM). R_F_-ODN tags were prepared by a solid-phase synthesis using phosphoramidite chemistry (details in Supporting Information, Figure S1), with the lengths of R_F_ including R_F4_, R_F6_, and R_F8_ variants. In addition, branched designs (1-, 2-, and 4-branched; referred to as R_F8_, (R_F8_)_2_, and (R_F8_)_4_, respectively) were prepared using a branching phosphoramidite installed at the 5′ end to modulate the density of perfluorinated groups (Figure 2).

### Limited Assembly Control when Fluorous Tags Are Added to Prefolded Origami

The first phase of this study focused on determining which, if any, of the fluorous tags could promote dimerization of the origami. The tags, in order of increasing fluorous content, were R_F4_, R_F6_, R_F8_, (R_F8_)_2_, and (R_F8_)_4_. It was anticipated that higher fluorous content would lead to stronger interactions between origami and thus higher dimerization yields.

First, the origami structure was modified with one of the fluorous tag variants, at all 14 positions along its short edge, Figure 2a, with the rate of dimerization measured using AFM and AGE. Unmodified origami, and origami containing nonfluorous alkyl tags, were included as controls. Analysis (Figure 2b,c) reveals that monomers dominated when the R_F4_ and R_F6_ tags were used. Using the R_F8_ tag resulted in the formation of some higher-order structures, including dimers and larger aggregates (visible on the AFM and observed in the gel as bands with lower mobility). When using the (R_F8_)_2_ tag there was a clear shift toward dimers as the predominant formation (although larger aggregates were also present). This tendency toward dimerization did not appear for the (R_F8_)_4_ tags, which produced a mixture of formations. Larger aggregates were particularly prominent for this variant in AFM analysis, although monomers appear to dominate in the AGE analysis. No origami assembly was observed when unmodified structures were used, and, significantly, no assembly was observed for the alkyl tag, which is directly comparable in size to the fluorous R_F8_ tag, confirming that origami-to-origami assembly is specifically driven by the fluorous effect rather than a general hydrophobic effect.

While these results demonstrate that the fluorous effect can promote origami assembly when all 14 modification sites are used, we next wanted to explore how the strength of the fluorous effect could be modulated by changing the number of R_F_-ODNs included in each structure. Having obtained the highest dimer yield using the (R_F8_)_2_ tag, we systematically varied the number of (R_F8_)_2_-ODNs incorporated into the origami (from 2 to 14, Figure 2d). Gel and AFM analyses confirm that dimers are the major species when four or more (R_F8_)_2_ tags are used (Figure 2e,f). As the number of tags increased, we also observed an increase in aggregates. Taken collectively, these results show that the assembly of individual origami nanostructures into higher-order networks can be directed by the fluorous effect and that the strength of the fluorous interaction can be tuned by altering the number and density of the R_F_-ODN tags (to prioritize controlled dimerization over aggregation, for example).

### Greater Assembly Control and Superior Dimer Yield when Fluorous Staples Are Included in the Origami Folding Phase

**Figure 3:**
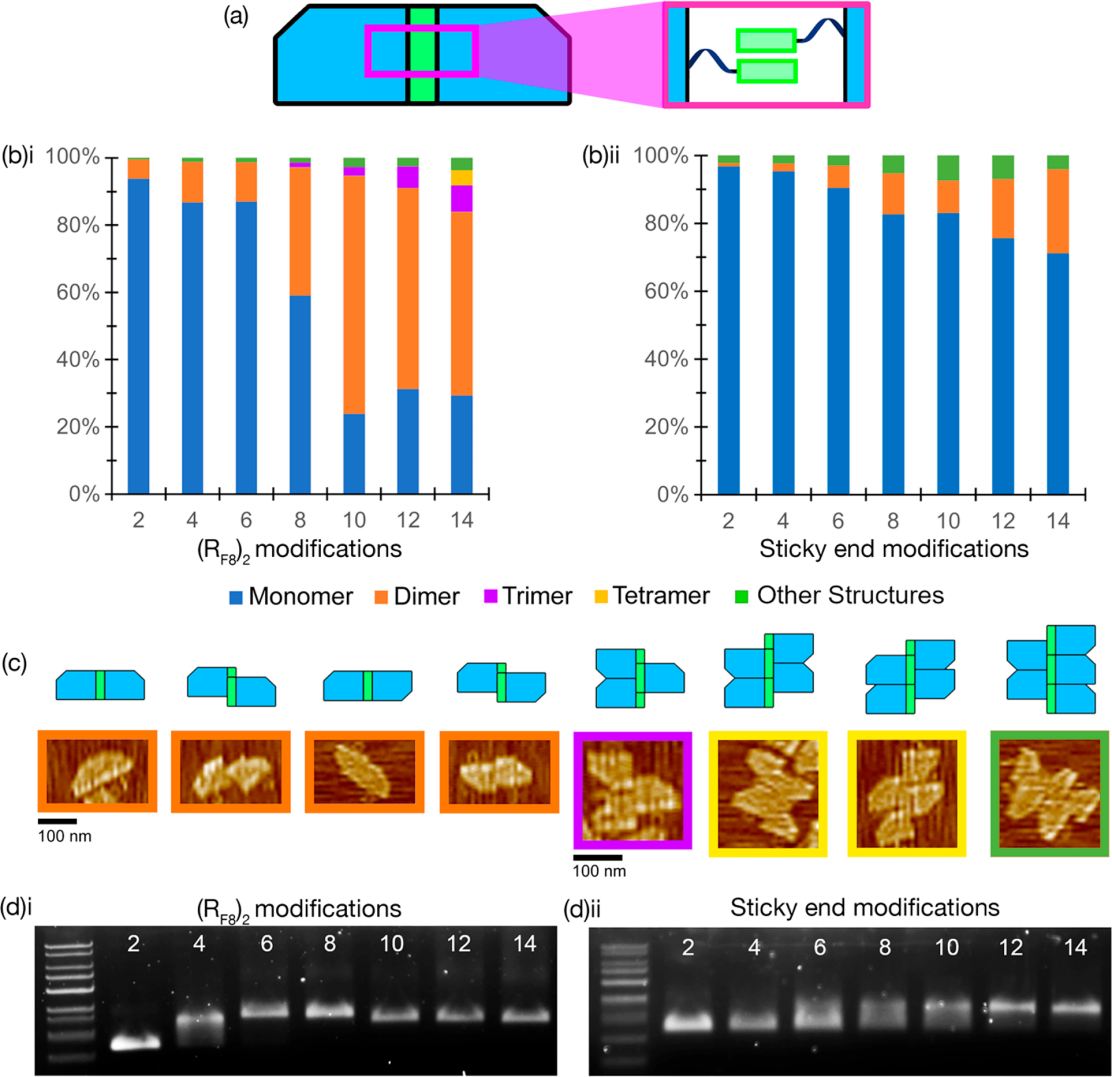
Origami assembly using integrated R_F_ staples; comparison of assembly via sticky ends and fluorous (R_F8_)_2_ tags. (**a**) Schematic showing dimerization via fluorous-modified staples. (**b**) AFM data analysis showing origami dimerization rates for (**i**) (R_F8_)_2_ staples and (**ii**) DNA sticky ends. (**c**) AFM images of origami with 14 (R_F8_)_2_ staples, from (b), showing the existence of dimers, trimers, tetramers, and pentamers. (**d**) AGE analysis of assembly using 2–14 overhangs of either (**i**) (R_F8_)_2_ staples or (**ii**) DNA sticky ends.

Having demonstrated fluorous-directed dimerization and established guidelines for the type and number of fluorous tags necessary to achieve assembly, we sought to refine the design by integrating the fluorous tags at the origami folding stage (as fluorous-modified staple strands), rather than add them to preformed nanostructures, as we did for the results shown in Figure 2. Significantly, this minimizes the distance between the origami edge and the end of the recognition tag (comparing Figure 3a to Figure 2**(a)ii**), reducing conformational flexibility, improving the likelihood of successful contact between elements (particularly in systems that include both R_F_-tags and DNA sticky ends), and decreasing the likelihood of unwanted aggregation between multiple origami structures. A series of origami nanostructures was prepared using this technique, with a systematic increase in (R_F8_)_2_-staples from 2 to 14. An equivalent series of nanostructures was also prepared, which incorporated sticky ends at the equivalent sites.

An AFM comparison between the R_F_ staple and sticky end systems (Figure 3**(b)i**,**ii**, respectively) shows that, for all modification numbers, the fluorous effect is significantly more efficient than DNA hybridization at promoting dimer assembly. The largest yield improvement observed was for the 10-tag systems, where the fluorous-directed approach produced a 7.4x increase in yield. Generally, the percentage of dimer formations increases with the number of R_F_ staples used, until we reach 12 and 14 R_F_ staples, at which point trimers, tetramers, pentamers, and higher-order aggregates begin appearing in larger numbers. However, as can also be seen in both the AFM (Figure 3b) and AGE results (Figure 3d) the use of integrated R_F_ staples significantly reduces the formation of higher-order structures compared to the R_F_-tag system shown in Figure 2. It appears that the shorter, less conformationally flexible R_F_ staples lead to more stringent spatial requirements to achieve effective alignment between fluorous groups, leading to a reduction of larger, less structured aggregates.

Although these results show that the fluorous effect is a powerful means of driving origami assembly, the range of slipped-dimers and higher-order structures shown in Figure 3c demonstrates that additional design considerations are necessary to direct the formation of specific, discrete assemblies that would be suitable as building blocks for the creation of far larger engineered assemblies. We anticipated that a blended approach that used DNA hybridization to complement the fluorous effect would be a potent strategy to address this issue, making use of the fluorous effect to drive dimer assembly and the sequence selectivity of sticky end hybridization as a means for controlling the relative orientation between origami tiles.

### Optimizing the Number of Fluorous Tags for Combination with DNA Base-Pairing

**Figure 4:**
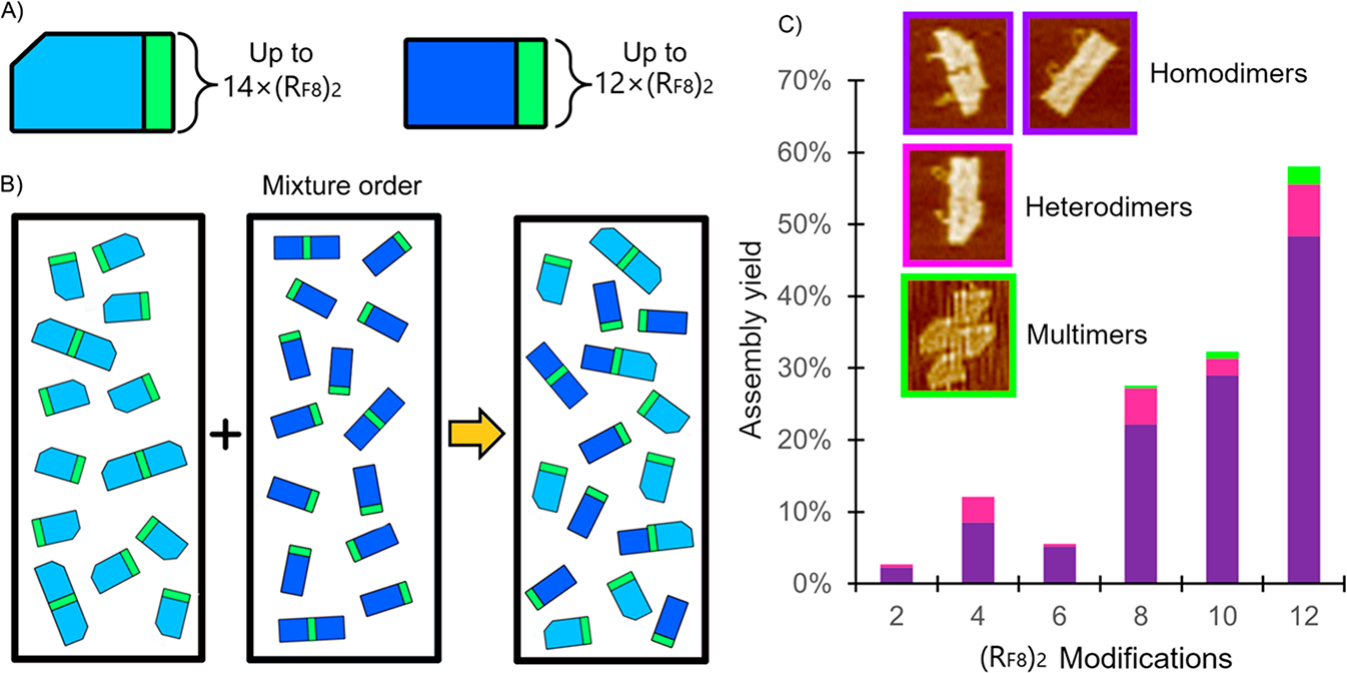
(**a**) Schematic representation of the two monomer designs. The rectangular origami is shorter than the origami used previously and only has 12 staple strands available for edge modification. (**b**) Individual monomer motifs are folded before being mixed together. (**c**) AFM analysis of the observed homodimer, heterodimer, and multimer yields of origami after the two designs are mixed.

To explore systems that contain both fluorous and ssDNA assembly tags it is necessary that we differentiate between dimers that occur solely because of the fluorous effect and those that are the product of ssDNA and fluorous working together. In any fluorous-assisted system that comprises two or more origami designs, a challenge will be preventing individual designs from adhering to each other before they are mixed with their counterparts, preventing hierarchical assembly. It is therefore necessary to establish how many fluorous tags can be added to each origami before homodimer formation (single designs sticking to each other) becomes too high, while also still leaving room along the origami edge for the addition of ssDNA sticky ends. To do this, we created a rectangular origami design that could easily be distinguished from the motifs used thus far (Figure 4a). We incorporated and systematically increased the number of (R_F8_)_2_ staples in both origami motifs, folding them separately before mixing both designs (Figure 4b).

AFM analysis revealed that homodimers predominate over heterodimers with the overall dimerization rate increasing with the number of (R_F8_)_2_ staples integrated into the designs. The small number of heterodimers may indicate that the fluorous interactions are relatively stable, with little dissociation and recombination occurring. This suggests that, if the fluorous-driven dimerization is too stable (because too may fluorous tags have been used), the inclusion of ssDNA sticky ends may be insufficient to dissociate the homodimers and promote heterodimer formation. Since 8, 10, and 12 R_F_ tags result in a high degree of dimerization and a small number of multimers (2.5% for 12 R_F_ tags) (Figure 4c), they are less suitable as candidates for a system containing both fluorous and DNA recognition. Origami with fewer R_F_ tags (2, 4, 6), where the fluorous-driven dimerization is less pronounced, the number of remaining attachment sites is high, and no multimers are formed, would offer a flexible platform for integration with Watson–Crick base-pairing, i.e., integrating fluorous-driven association with sequence-selective molecular recognition of DNA sticky ends.

### Combining Fluorous- and Watson–Crick-Directed Molecular Recognition

**Figure 5:**
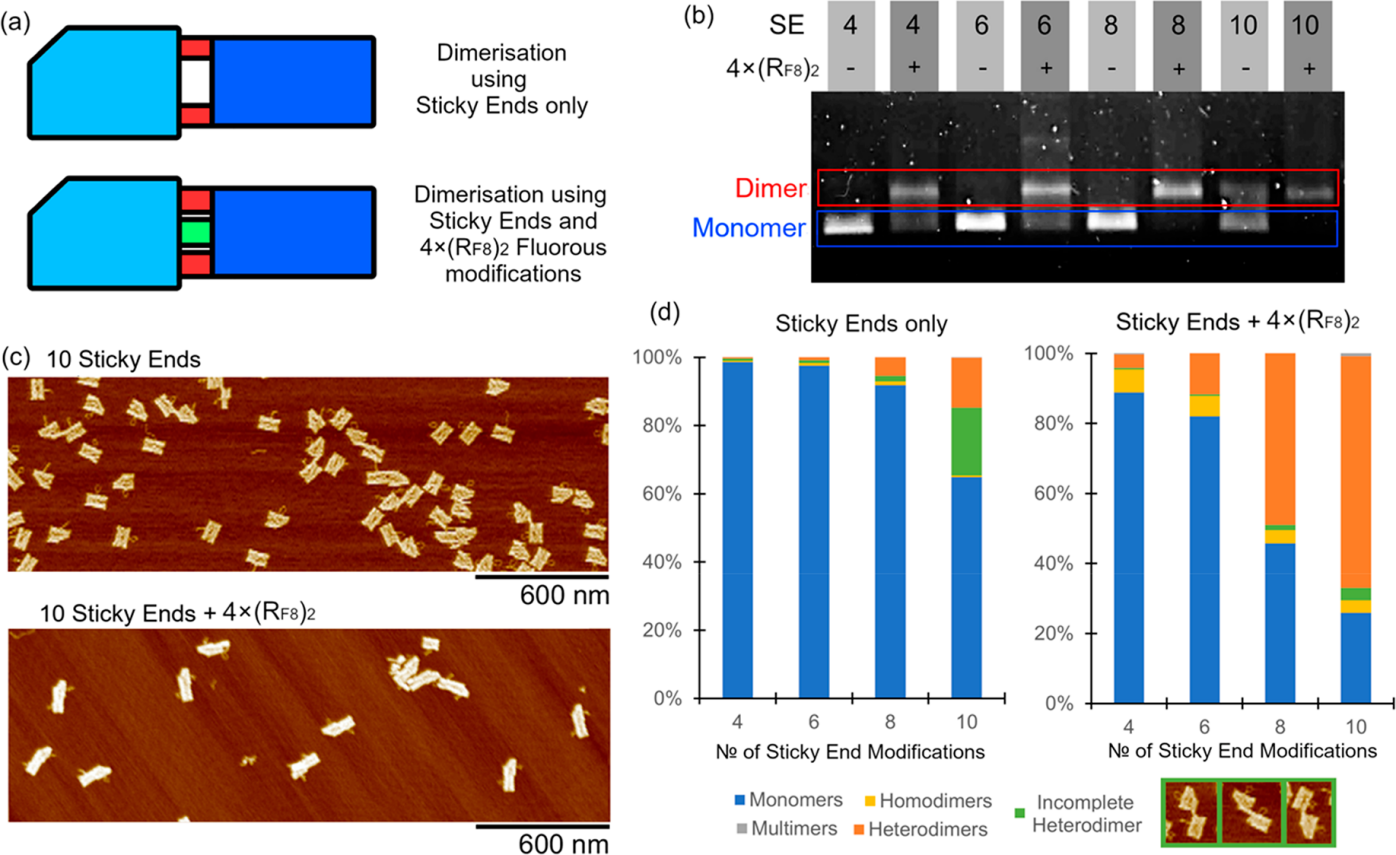
(a) Schematics highlighting the relative position of the fluorous core and flanking sticky-end recognition elements. The central fluorous core contains four (RF_8_)_2_ tags, with sticky-end overhangs placed on either side of this fluorous region. (b) AGE data showing heterodimerization driven by a combination of the fluorous effect and sticky-end association. (c) Representative AFM images used for heterodimer analysis showing the origami dimerization driven by sticky ends only (top) and the impact of combining sticky ends with fluorous recognition (bottom). (d) AFM analysis of observed assembly yields formed with origami containing sticky ends and those containing both fluorous and sticky ends.

Our studies show that the strategic placement of perfluorinated tags at the interface of origami tiles is an effective strategy for the assembly of higher-order origami nanostructures. Although discrete dimeric species are the major products of our fluorous-directed assembly experiments, the formation of slipped dimers, multimers, and larger aggregates is inevitable given the nonspecific, mobile nature of the fluorous effect. We therefore explored the potential to enhance the formation of discrete origami heterodimers by combining fluorous and Watson–Crick modes of molecular recognition. To this end, a series of single-stranded, five-nucleotide sticky ends were integrated into the origami structures in positions flanking a central fluorous core comprising four (R_F8_)_2_ tags, Figure 5a (chosen based on Figure 4, where small tag numbers show measurable effects while leaving space for ssDNA sticky ends). Control structures that did not contain the fluorous core were also prepared. We hypothesized that the fluorous core would act as a “catch” and that the sequence selectivity imparted by complementary Watson–Crick base-pairing would act as a “latch” to anchor the formation of stable heterodimers.

Gel shift and AFM analyses show an increase in the formation of heterodimers when the number of flanking single-stranded DNA overhangs is increased (Figures 5b–d). Minimal to no heterodimerization was observed without the presence of the fluorous core, indicating that the fluorous effect is a driving force for hierarchical assembly in this system. Quantification of heterodimerzation by AFM (Figure 5d) shows that, when using only sticky-end recognition, the highest heterodimer yield achieved was 23% (10 sticky ends), with a large proportion of partially assembled, incomplete heterodimers (19.9%). In contrast, a combined molecular recognition approach with 10 sticky ends flanking the central fluorous core forms heterodimers in 77% yield (a threefold enhancement in heterodimerization) with a markedly lower proportion of incomplete heterodimers (3.6%), indicating that the presence of a fluorous core helps the dimer assembly “snap shut”. The comparative yield of heterodimerization is even more apparent using eight sticky ends (i.e., four each side of the fluorous core) where using Watson–Crick base-pairing alone gives 7% yield, while the mixed recognition approach results in the formation of heterodimers at 56% (an eightfold yield increase). The proportion of heterodimers formed in this combined sticky-end/fluorous system, Figure 5d, is much higher than observed when fluorous tags are used alone, Figure 4c, demonstrating that sticky ends assist in dimer exchange, shifting the equilibrium in favor of the heterodimer product and supporting a catch and latch type of assembly mechanism. The combination of these two molecular recognition modes also results in the virtual elimination of higher-order multimers (trimers and above); multimers comprised 0.3, 0.0, 0.0, and 0.8% of the 4, 6, 8, and 10 SE + 4x(RF)_2_ variants, respectively. This suggests that fluorous-directed molecular recognition works in concert with Watson–Crick base-pairing to maximize the formation of discrete assemblies. We believe that these results show the potential for fluorous staples to benefit more complex superstructures, where, in tandem with the specificity provided by DNA recognition, it could act as a strong, mobile binding element to increase the yield of larger hierarchical assemblies while minimizing the need for bespoke staple production.

## Conclusion

In summary, we have presented an atom-efficient molecular recognition approach to direct the hierarchical assembly of DNA origami nanostructures. Integrating Watson–Crick base-pairing with the fluorous effect results in a complementary palette of noncovalent interactions, which directs the formation of discrete dimeric species in our model origami system with an efficiency greater than when either molecular recognition approach is used in isolation. Since this method of origami attachment is orthogonal to DNA-based methods, the benefits we have demonstrated will be transferrable to other DNA-based origami tiling systems, including complex three-dimensional assemblies. The combination of its ease of synthesis, its small size, and the relative strength and mobility of the fluorous effect makes R_F_-DNA a powerful tool in the self-assembly arsenal of structural DNA nanotechnology and may prove to be useful in the formation of functional assemblies that span into the micron-scale. Furthermore, interfacing fluorous-directed assembly of DNA nanostructures with other components could provide a molecular blueprint to enhance the delivery of biological components to specific cell types.^29−31^

## Methods

### Synthesis of Fluorous ODN Strands

Fluorous-tagged oligonucleotides were synthesized using standard solid-phase methods on an Applied Biosystems 392 DNA/RNA synthesizer. DNA synthesis reagents and solutions were purchased from Link Technologies Ltd. Oligonucleotides were purified by reverse-phase high-performance liquid chromatography (RP-HPLC) and characterized by matrix-assisted laser desorption/ionization mass spectrometry (MALDI-MS).

### Assembly of DNA Origami

Single-stranded M13mp18 DNA was purchased from Tilibit nanosystems (type p7249, 2000uL at 100 nM). Staple strands (sequences listed in Supporting Information) were purchased unpurified from Integrated DNA Technologies (IDT) in 96-well plates, suspended in water, and normalized to 100 μM. DNA origami were folded in a buffer solution containing 1 × TAE (Tris, 40 mM; acetic acid, 20 mM; EDTA, 1 mM) with 12.5 mM magnesium acetate. M13mp18 concentrations ranged from 4 to 10 nM, with staple strands being at a 10x molar excess. Solutions were heated to 95 °C and cooled to 25 °C at a rate of 1 °C/min.

For origami with modified edges, a core set of staples was maintained. Sets were then made, with each containing the unique staple strands for that given setup.

Heterodimer assemblies were made by mixing the filtered monomer solutions together. Samples were incubated at 25 °C for 12 h with a monomer-equivalent concentration of 4 nM.

### Filtration of Staple Strands

Excess staple strands were removed using Amicon Ultra-0.5 mL 100 kDa. 500 μL of 1xTAE-Mg2+ buffer was added and spun at 10 000*g* for 5 min. Then 370 μL of buffer along with 100 μL of the unfiltered origami was added and spun at 5000*g* for 7 min. A further 470 μL of buffer was added and spun at 5000*g* for 7 min. The sample was then inverted and placed into a fresh 0.5 mL tube and spun at 13 000*g* for 2 min. The concentration of filtered origami was measured by using a UV/vis spectrophotomer (NanoDrop Lite Spectrophotometer, Thermo Scientific).

### Agarose Gel Electrophoresis

4 μL of loading dye (6X loading dye, ThermoFisher Scientific, #60111) was added to 20 μL of annealed and filtered samples (1 nM monomer-equivalent) before being loaded into the wells. Four microliters of DirectLoadT 1 kb ladder (Sigma-Aldrich, #D3937-1VL) was used as a reference. The gel consisted of 1% agarose containing 1xMg-TAE buffer stained with SYBR safe (ThermoFisher Scientific, #S33102). The electrophoresis voltage was set to 80 V and run in an ice bath for a total of 90 min.

### Atomic Force Microscopy

Ten microliters of sample containing 1 nM of origami were deposited onto freshly cleaved mica and left to adsorb for 2 min. Samples were then rinsed with DI water before being dried under a weak flux of N_2_ for 10 s. Samples were imaged under ambient conditions using Tapping Mode on a Dimension Icon (Bruker) with FESPA-V2 probes. For counts of origami assemblies made from AFM images, *n* > 275 for each dimer type. Typical scan parameters were scan rate: 1 Hz, resolution: 3072 × 1024, area: 10 μm, amplitude set point: 250 mV, drive frequency: 75 kHz, drive amplitude: 1000 mV.

## Data Availability

All data relating to the work outlined in the article can be found at: 10.5525/gla.researchdata.1353.

## Supporting Information Available

Details of: DNA synthesis; Origami assembly; Gel electrophoresis; AFM and gel imaging; All DNA sequences used; HPLC of modified sequences; and DNA modeling (DOCX)
