# Supplementary material for: Fluorous-Directed Assembly of DNA Origami Nanostructures
Source: ACS Nano. 2022 Dec 20;17(1):752–9. doi: 10.1021/acsnano.2c10727 (PMC9835977; doi:10.1021/acsnano.2c10727)
Supplement: Supplementary file 1 — nn2c10727_si_001.pdf [file nn2c10727_si_001.pdf]

Fluorous Directed Assembly of DNA Origami Nanostructures

Supporting Information

Jiajia Zou,[a§] Ashley C. Stammers,[a§] Andrea Taladriz-Sender,[b] Jamie M. Withers,[a] Iain Christie,[a] Marina Santana Vega,[a] Badri L. Aekbote,[a] William J. Peveler,[c] David A. Rusling,[d] Glenn A. Burley,[b*] Alasdair W. Clark[a*]

[§] These authors contributed equally to this work

[a] James Watt School of Engineering, Advanced Research Centre, University of Glasgow, Glasgow, G11 6EW, United Kingdom. *Email: alasdair.clark@glasgow.ac.uk

[b] Department of Pure Applied Chemistry, Thomas Graham Building, 295 Cathedral Street, University of Strathclyde, Glasgow, G1 1XL, United Kingdom. Email: glenn.burley@strath.ac.uk

[c] School of Chemistry, Joseph Black Building, University of Glasgow, Glasgow, G12 8QQ, United Kingdom.

[d] School of Pharmacy and Biomedical Sciences, St. Michael's Building, University of Portsmouth, Portsmouth, PO1 2DT, United Kingdom.

DNA Synthesis

Oligodeoxyribonucleotides (ODN) were synthesized according to standard solid-phase oligonucleotide-synthesis protocols on an ABI 392 DNA/RNA synthesizer on a 1 μM scale. Coupling efficiency was monitored after removal of the dimethoxytrityl (DMTr) 5'-OH protecting groups. Controlled pore glass (CPG, 1000Å/110mm) supports loaded with standard nucleosides and standard phosphoramidites were purchased from LINK-LGC Biosearch Technologies.

Special branched phosphoramidite **1** (0.1 M solution in CH_3CN), alkyl phosphoramidite **5** and single fluorous phosphoramidites **2**-**4** (0.2 M solution in CH_3CN) were introduced at the 5' position using a 1 μM CE cycle. When incorporating multiple fluorous tags using phosphoramidite **4** (0.2 M solution in CH_3CN) after branched phosphoramidite **1**, a modified cycle was used with longer coupling times (double coupling, 3 min each).

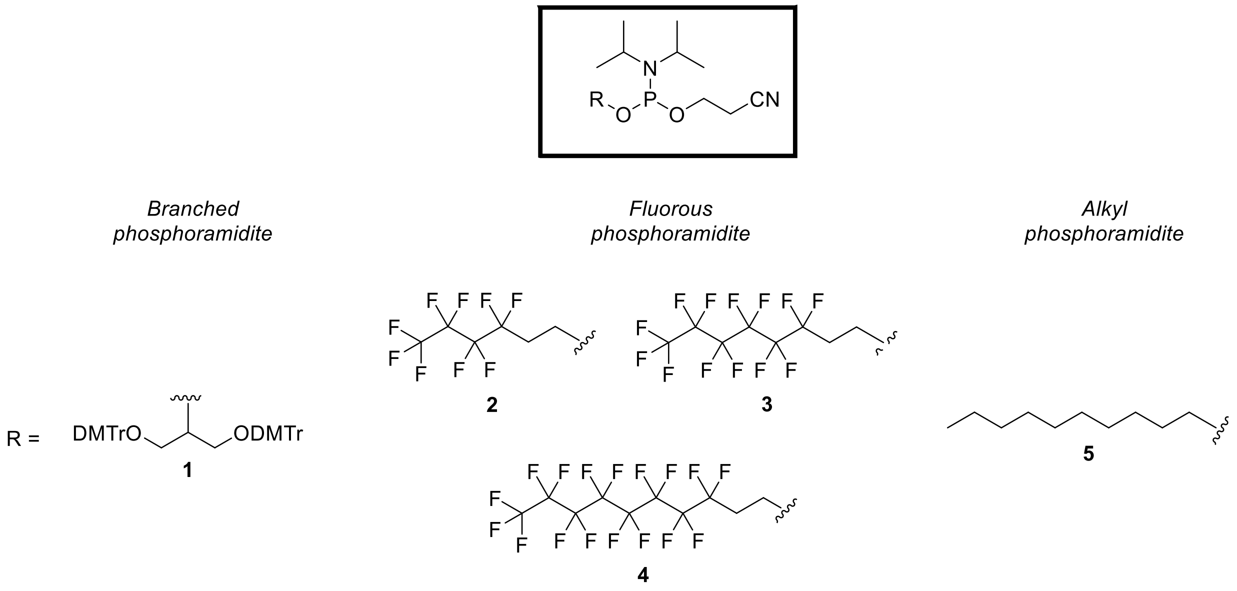

Figure S1. Structures of the phosphoramidites used in the synthesis of the modified strands. Their synthesis and characterisation will be reported elsewhere.

Cleavage and deprotection procedure: ODNs were cleaved from the solid support and deprotected in 28% aqueous ammonium hydroxide solution for 4 h at 55°C or overnight at rt. The crude product solution was separated from the solid support and concentrated under reduced pressure at 30 °C in a speed vacuum concentrator. This crude solid was re-suspended in 1 mL Millipore water and purified by HPLC.

HPLC purification: Analytical RP-HPLC was performed at rt on an ULTIMAT 3000 Instrument (DIONEX). UV absorbance was measured using a photodiode array detector at 260 nm. A Clarity Oligo RP C18 (10 × 250 mm, 5 µm) column was used for semi-prep RP-HPLC. Solvent system: Buffer A, 0.1 M Triethylammonium acetate (TEAA) in H_2O, pH 7.4; Buffer B: 0.1 M TEAA, pH 7.6, 80% MeCN and 20% H_2O. Flow rate: 3.0 mL/min.

Gradient profile:

Time (min)	%A	%B
**0**	90	10
**3**	90	10
**38**	0	100
**50**	0	100
**51**	90	10
**60**	90	10

Table S1. Solvent gradient profile for HPLC purification.

Assembly of DNA origami nanotiles

The rectangle and truncated rectangle tiles were folded in a buffer solution containing 1 x TAE (40 mM Tris, 20mM Acetic acid, 1mM EDTA, pH 8.4) with 12.5 mM Magnesium acetate. M13mp18 concentrations ranged from 4 nM to 10 nM, with staple strands at a 10x molar excess. Solutions were heated to 95°C and cooled to 25°C at a rate of 1°C/min.

Excess staple strands were removed using Amicon Ultra-0.5 mL 100 kDa. 500 µL of 1xTAE-Mg^{2+} buffer was added and spun at 10,000 G for 5 min. Then 370 µL of buffer along with 100 µL of the unfiltered origami was added and spun at 5,000 G for 7 min. A further 470 µL of buffer was added and spun at 5,000 G for 7 min. The sample was then inverted and placed into a fresh 0.5 mL tube and spun at 13,000 G for 2 min.

The concentrations of filtered origami were measured by using a NanoDrop Lite Spectrophotometer (Thermo Scientific). The extinction coefficient used was 91886000 L $mol^{-1}cm^{-1}$.

Edge-modified origami of the type shown in **Figure 2** were made by first annealing the truncated rectangle, including 'capture' staples (sequences below) which have single stranded regions that extend beyond the edge of the origami tile. After annealing and removal of excess staples by spin filtration, a tenfold excess of the modified ODNs (the 'captured' strand) was added and allowed to hybridise. Excess captured strands were then removed by spin filtration.

Edge modified origami of the type shown in **Figures 3**, **4**, and **5** were made by including all staples, including modified staples (referred to as 'integrated' staples, below), during the initial annealing step. Excess staples were then removed by spin filtration.

Dimer constructs were made by mixing the two filtered monomer solutions together. Samples were incubated at 25°C for 12 h. Origami concentration used for was 4 nM homodimer assembly and 2 nM of each tile for heterodimers.

Gel electrophoresis

1% agarose in TBE buffer (pH 8.4), 80V, 90 min. An ice bath was used to control temperature. DNA loading dye (6x, Thermo Scientific) was added to samples immediately prior to loading onto the gel. Samples were compared against a GeneRuler 1kb DNA ladder (Thermo Scientific).

AFM imaging

10 µL of samples containing 1 nM of origami were deposited onto freshly cleaved mica and left to adsorb for 2 min. Samples were then rinsed with DI water before being dried under a weak flux of N_2 for 10 s. Samples were imaged using a Bruker Dimension Icon instrument using FESPA-V2 probes, tapping mode in air. Collected AFM images were analysed using the cell counter function in ImageJ. Yields were calculated based on the total number of individual origami tiles in each type of assembly.

Truncated Origami tile (Figures 2, 3, 4, 5)

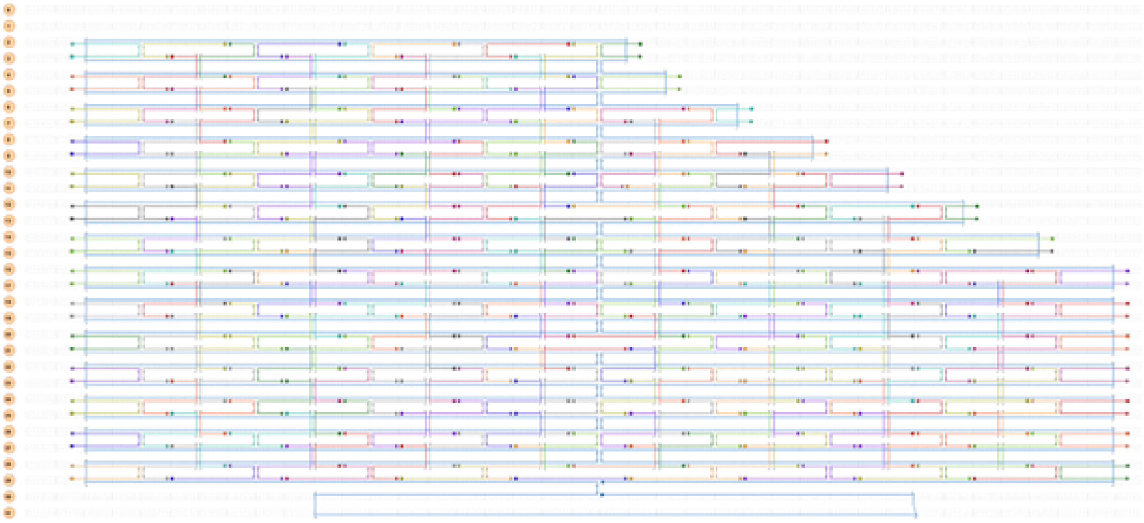

Figure S2. CaDNAno schematic of the truncated rectangle origami used in figures 2, 3, 4, and 5. This tile is modified from the rectangle origami such that the left-hand side of both share staple sequences.

Truncated-rectangle origami staples

3[12]2[12]	TTTTATAAATCCTCATTAAATGATATTCACAAACAATTTT
5[12]4[12]	TTTTTATAAGTATAGCCCGGCCGTCGAGAGGGTTGATTTT
7[12]6[12]	TTTTAACTTTCAACAGTTTCTGGGATTTTGCTAAACTTTT
9[12]8[12]	TTTTGACAGCATCGGAACGAACCCTCAGCAGCGAAATTTT
11[12]10[12]	TTTTTCATAAGGGAACCGAAAGGCGCAGACGGTCAATTTT
13[12]12[12]	TTTTGGAAGAAAAATCTACGACCAGTCAGGACGTTGTTTT
15[12]14[12]	TTTTCATTGAATCCCCCTCAAATCGTCATAAATATTTTTT
17[12]16[12]	TTTTCTGTAGCTCAACATGTATTGCTGAATATAATGTTTT
19[12]18[12]	TTTTGGTTGTACCAAAAACAAGCATAAAGCTAAATCTTTT
21[12]20[12]	TTTTGATGAACGGTAATCGTAGCAAACAAGAGAATCTTTT
23[12]22[12]	TTTTGTAATGGGATAGGTCAAAACGGCGGATTGACCTTTT
25[12]24[12]	TTTTTGTAAAACGACGGCCATTCCCAGTCACGACGTTTTT
27[12]26[12]	TTTTCGGTTTGCGTATTGGGAACGCGCGGGGAGAGGTTTT
29[12]28[12]	TTTTCGATGGCCCACTACGTAAACCGTCTATCAGGGTTTT
3[40]5[39]	GGAAAGCGACCAGGCGGATAAGTGAATAGGTG
5[40]7[39]	TATCACCGAAATGAATTTTCTGTAAGCGGAGT
7[40]9[39]	GAGAATAGCTTTTGCGGGATCGTCGGGTAGCA
9[40]11[39]	ACGGCTACTTACTTAGCCGGAACGCTGACCAA
11[40]13[39]	CTTTGAAAAGAACTGGCTCATTATTTAATAAA
13[40]15[39]	ACGAACTAGCGTCCAATACTGCGGAATGCTTT
15[40]17[39]	AAACAGTTGATGGCTTAGAGCTTATTTAAATA
17[40]19[39]	TGCAACTAAGCAATAAAGCCTCAGTTATGACC
19[40]21[39]	CTGTAATATTGCCTGAGAGTCTGGAAAACTAG
21[40]23[39]	CATGTCAAGATTCTCCGTGGGAACCGTTGGTG
23[40]25[39]	TAGATGGGGGGTAACGCCAGGGTTGTGCCAAG
25[40]27[39]	CTTGCATGCATTAATGAATCGGCCCGCCAGGG
27[40]29[39]	TGGTTTTTAACGTCAAAGGGCGAAGAACCATC
29[40]28[56]	ACCCAAATCAAGTTTTTTGGGGTCAAAGAACG
2[55]3[39]	TGAGGCAGGTCAGACGATTGGCCTGCCAGAAT
4[55]2[56]	TGCTCAGTCAGTCTCTGAATTTACCAGGAGGT
6[55]4[56]	ACGTTAGTTACTCAGGAGGTTTAGCGGGGTTT

8[55]6[56]	AAAGGCCGAAAGGAACAACTAAAGCTTTCCAG
10[55]8[56]	GCTCCATGAGAGGCTTTGAGGACTAGGGAGTT
12[55]10[56]	CGATTTTAGAGGACAGATGAACGGCGCGACCT
14[55]12[56]	ACTGGATAACGGAACAACATTATTACCTTATG
16[55]14[56]	TTTTTGCGCAGAAAACGAGAATGAATGTTTAG
18[55]16[56]	CAAAATTAAAGTACGGTGTCTGGAAGAGGTCA
20[55]18[56]	TCAGGTCACTTTTGCGGGAGAAGCAGAATTAG
22[55]20[56]	ACCCGTCGTCATATGTACCCCGGTAAAGGCTA
24[55]22[56]	ATTAAGTTCGCATCGTAACCGTGCGAGTAACA
26[55]24[56]	GCCAGCTGCCTGCAGGTCGACTCTGCAAGGCG
28[55]26[56]	TGGACTCCCTTTTCACCAGTGAGACCTGTCGT
3[72]5[71]	TAAGCGTCGAAGGATTAGGATTAGTACCGCCA
5[72]7[71]	CCCTCAGATCTAAAGTTTTGTCGTGAATTGCG
7[72]9[71]	AATAATAAGGTCGCTGAGGCTTGCAAAGACTT
9[72]11[71]	TTTCATGAAAATTGTGTCGAAATCTGTACAGA
11[72]13[71]	CCAGGCGCTTAATCATTGTGAATTACAGGTAG
13[72]15[71]	AAAGATTCAGGGGGTAATAGTAAACCATAAAT
15[72]17[71]	CAAAAATCATTGCTCCTTTTGATAAGTTTCAT
17[72]19[71]	TCCATATACATACAGGCAAGGCAACTTTATTT
19[72]21[71]	CAACGCAATTTTTGAGAGATCTACTGATAATC
21[72]23[71]	AGAAAAGCAACATTAAATGTGAGCATCTGCCA
23[72]25[71]	GTTTGAGGGAAAGGGGGATGTGCTAGAGGATC
25[72]27[71]	CCCGGGTACTTTCCAGTCGGGAAACGGGCAAC
27[72]29[71]	AGCTGATTACAAGAGTCCACTATTGAGGTGCC
29[72]28[88]	GTAAAGCACTAAATCGGAACCCTAGTTGTTCC
2[87]3[71]	CACCAGAGCCGCCGCCAGCATTGACGTTCCAG
4[87]2[88]	CCTCAAGAATACATGGCTTTTGATAGAACCAC
6[87]4[88]	CGTAACGAACCGCCACCCTCAGAACTGAGACT
8[87]6[88]	ATATATTCTTTTTTCACGTTGAAAATAGTTAG
10[87]8[88]	CGCCTGATGGAAGTTTCCATTAAACATAACCG
12[87]10[88]	TTTCAACTATAGGCTGGCTGACCTTGTATCAT
14[87]12[88]	TTTGCCAGATCAGTTGAGATTTAGTGGTTTAA
16[87]14[88]	TACCTTTAAGGTCTTTACCCTGACAAAGAAGT
18[87]16[88]	CAATAAATACAGTTGATTCCCAATTTAGAGAG
20[87]18[88]	GGTAGCTAGGATAAAAATTTTTAGTTAACATC
22[87]20[88]	CTTTCATCCCCAAAAACAGGAAGACCGGAGAG
24[87]22[88]	CAGCTGGCGGACGACGACAGTATCGTAGCCAG
26[87]24[88]	ACTGCCCGCCGAGCTCGAATTCGTTATTACGC
28[87]26[88]	AGTTTGGAGCCCTTCACCGCCTGGTTGCGCTC
3[104]5[103]	AGTGTACTTGAAAGTATTAAGAGGCCGCCACC
5[104]7[103]	CTCAGAGCTCCACAGACAGCCCTCATCTCCAA
7[104]9[103]	AAAAAAGGACAACCATCGCCCACGCGGGTAAA
9[104]11[103]	ATACGTAAAAGTACAACGGAGATTTCATCAAG
11[104]13[103]	AGTAATCTTAAATTGGGCTTGAGAGAATACCA
13[104]15[103]	CATTCAACGCGAGAGGCTTTTGCATATTATAG
15[104]17[103]	TCAGAAGCCTCCAACAGGTCAGGATCTGCGAA
17[104]19[103]	CGAGTAGAACTAATAGTAGTAGCAAACCCTCA
19[104]21[103]	TATATTTTAGCTGATAAATTAATGTTGTATAA
21[104]23[103]	GCAAATATCGCGTCTGGCCTTCCTGGCCTCAG
23[104]25[103]	GAAGATCGGTGCGGGCCTCTTCGCAATCATGG
25[104]27[103]	TCATAGCTACTCACATTAATTGCGCCCTGAGA
27[104]29[103]	GAGTTGCACGAGATAGGGTTGAGTAAGGGAGC
29[104]28[120]	CCCCGATTTAGAGCTTGACGGGGAAATCAAAA
2[119]3[103]	GCCACCACCCTCAGAGCCGCCACCGATACAGG
4[119]2[120]	CTGAAACAGGTAATAAGTTTTAACCCCTCAGA
6[119]4[120]	TGTAGCATCACCACCCTCATTTTCCTATTATT
8[119]6[120]	CAATGACACTCCAAAAGGAGCCTTACAACGCC
10[119]8[120]	GCGAAACATGCCACTACGAAGGCATGCGCCGA
12[119]10[120]	ACGAGTAGTGACAAGAACCGGATATACCAAGC
14[119]12[120]	CCAAAATATAATGCAGATACATAAACACCAGA
16[119]14[120]	GAAGCAAAAAAGCGGATTGCATCAGATAAAAA
18[119]16[120]	TCAATTCTTTTAGTTTGACCATTACCAGACCG

20[119]18[120]	ACCGTTCTAAATGCAATGCCTGAGAGGTGGCA
22[119]20[120]	AAATAATTTTAAATTGTAAACGTTGATATTCA
24[119]22[120]	GGCGATCGCACTCCAGCCAGCTTTGCCATCAA
26[119]24[120]	GTGAGCTAGTTTCCTGTGTGAAATTTGGGAAG
28[119]26[120]	GAATAGCCGCAAGCGGTCCACGCTCCTAATGA
3[136]5[135]	TGCCTTGACTGCCTATTTCGGAACAGGGATAG
5[136]7[135]	CAAGCCCAGTCACCAGTACAAACTTAATTGTA
7[136]9[135]	TCGGTTTAGCTTGATACCGATAGTCCAACCTA
9[136]11[135]	AAACGAAATGACCCCCAGCGATTATTCATTAC
11[136]13[135]	CCAAATCACTTGCCCTGACGAGAACGCCAAAA
13[136]15[135]	GGAATTACTCGTTTACCAGACGACAAAAGATT
15[136]17[135]	AAGAGGAACGAGCTTCAAAGCGAAGATACATT
17[136]19[135]	TCGCAAATGGGGCGCGAGCTGAAATAATGTGT
19[136]21[135]	AGGTAAAGAAATCACCATCAATATAATATTTT
21[136]23[135]	GTTAAAATTTTAACCAATAGGAACCCGGCACC
23[136]25[135]	GCTTCTGGTCAGGCTGCGCAACTGTGTTATCC
25[136]27[135]	GCTCACAATGTAAAGCCTGGGGTGGGTTTGCC
27[136]29[135]	CCAGCAGGGGCAAAATCCCTTATAAAGCCGGC
29[136]29[167]	GAACGTGGCGAGAAAGGAAGGGAACAAACTAT
2[151]3[135]	GAGCCGCCACCCTCAGAACCGCCAGGGGTCAG
4[151]2[152]	AATGCCCCGTAACAGTGCCCGTATCTCCCTCA
6[151]4[152]	TGAGTTTCATAGGAACCCATGTACAAACAGTT
8[151]6[152]	CTTAAACATCAGCTTGCTTTCGAGCGTAACAC
10[151]8[152]	CTCATCTTGAGGCAAAAGAATACAGTGAATTT
12[151]10[152]	GAATAAGGACGTAACAAAGCTGCTCTAAAACA
14[151]13[167]	CATAACCCGAGGCATAGTAAGAGCGCAAGAAA
16[151]14[152]	TTTTAATTGCCCGAAAGACTTCAAAACACTAT
18[151]16[152]	TTTCATTTGGTCAATAACCTGTTTATATCGCG
20[151]18[152]	AGACAGTCATTCAAAAGGGTGAGAAGCTATAT
22[151]21[167]	GCTCATTTTCGCATTAAATTTTTGAGCTTAGA
24[151]22[152]	TTCGCCATTGCCGGAAACCAGGCATTAAATCA
26[151]24[152]	GCATAAAGTTCCACACAACATACGAAGCGCCA
28[151]26[152]	CCGAAATCCGAAAATCCTGTTTGAAGCCGGAA
7[168]9[167]	AGTTTGCCCCGTAATCAGTAGCGACACCGACT
9[168]11[167]	TGAGCCATGGTGAATTATCACCGTAAAGAAAC
11[168]12[152]	GCAAAGACAAGGTGGCAACATATACATTCAGT
13[168]15[167]	CAATGAAAAAGCCCAATAATAAGATTCCAGAG
15[168]17[167]	CCTAATTTACGCTAACGAGCGTCTAATCAATA
17[168]19[167]	ATCGGCTGCGAGCATGTAGAAACCTATCATAT
19[168]20[152]	GCGTTATAGAAAAAGCCTGTTTAGAAGGCCGG
21[168]23[167]	TTAAGACGTTGAAAACATAGCGATAACAGTAC
23[168]25[167]	CTTTTACACAGATGAATATACAGTAAACAATT
25[168]27[167]	CGACAACTAAGTATTAGACTTTACAATACCGA
27[168]28[152]	ACGAACCAAAACATCGCCATTAAATGGTGGTT
29[168]28[184]	CGGCCTTGCTGGTAATATCCAGAACGAACTGA
2[171]3[171]	TTTTGGAACCGCCCACCACCTTTT
4[182]5[182]	TTTTAAAATCACCGGAACCAGAGTTGCCATCTTTTCATAATCTTTT
6[183]7[167]	CGGTCATAGCCCCCTTATTAGCGTCAGAATCA
8[183]6[184]	TAGCAGCATTTAGCGTCAGACTGTGGCATTTT
10[183]8[184]	TCATTAAATTGGGAATTAGAGCCAACCATCGA
12[183]10[184]	CATACATAACCACGGAATAAGTTTGAAATTAT
14[183]12[184]	ATTGAGTTTAGCAATAGCTATCTTAGAAAATA
16[183]14[184]	TCTTACCAGCCAGTTACAAAATAACCACAAGA
18[183]16[184]	CTAATTTATCTTTCCTTATCATTCATCCTGAA
20[183]18[184]	AATTACTACAAATTCTTACCAGTAATCCCATC
22[183]20[184]	TAGAATCCCTGAGAAGAGTCAATAGGAATCAT
24[183]22[184]	TTTAACGTTCGGGAGAAACAATAATTTTCCCT
26[183]24[184]	GGATTTAGCGTATTAAATCCTTTGTTTTCAGG
28[183]26[184]	TAGCCCTACCAGCAGAAGATAAAAACATTTGA
9[200]11[199]	ACCAGTAGAAGGTAAATATTGACGATTTTGTC
11[200]13[199]	ACAATCAAGTATGTTAGCAAACGTACCGAAGC
13[200]15[199]	CCTTTTTAATATCAGAGAGATAACACAGCCAT

15[200]17[199]	ATTATTTAACCCAGCTACAATTTTCAAGAACG
17[200]19[199]	GGTATTAAGAACAAGAAAAATAATTAAAGCCA
19[200]21[199]	ACGCTCAAAATAAGAATAAACACCGTGAATTT
21[200]23[199]	ATCAAAATCGTCGCTATTAATTAACGGATTCG
23[200]25[199]	CCTGATTGAAAGAAATTGCGTAGACCCGAACG
25[200]27[199]	TTATTAATGCCGTCAATAGATAATCAGAGGTG
27[200]29[199]	AGGCGGTCATTAGTCTTTAATGCGCAATATTA
29[200]28[216]	CCGCCAGCCATTGCAACAGGAAAAATATTTTT
6[202]7[202]	TTTTTTTCATCAGCGCGTTTTT
10[215]9[223]	AGGGAGGGCACCATTACCATTTTT
12[215]10[216]	ATTACGCATAGAAAATTCATATGGACCGATTG
14[215]12[216]	GAGCGCTAAGAAAAGTAAGCAGATGACTCCTT
16[215]14[216]	TATTTTGCTCCCAATCCAAATAAGGGGTAATT
18[215]16[216]	TAAGTCCTACCAAGTACCGCACTCTTAGTTGC
20[215]18[216]	AGGCGTTACAGTAGGGCTTAATTGACAATAGA
22[215]20[216]	CTGTAAATCATAGGTCTGAGAGACGATAAATA
24[215]22[216]	ACAGAAATCTTTGAATACCAAGTTCCTTGCTT
26[215]24[216]	AGATTAGATTTAAAAGTTTGAGTACACGTAAA
28[215]26[216]	GAATGGCTAGTATTAACACCGCCTCAACTAAT
8[223]9[199]	TTTTTAGCAAGGCCGGAAACGTCACCAATGAAGCAAAATC
13[232]15[231]	AAAGTTACCCTGAACAAAGTCAGAAAACGATT
15[232]17[231]	TTTTGTTTAAGCCTTAAATCAAGAATCGAGAA
17[232]19[231]	CAAGCAAGACGCGCCTGTTTATCAAGAATCGC
19[232]21[231]	CATATTTAGAAATACCGACCGTGTTACCTTTT
21[232]23[231]	TAACCTCCATATGTGAGTGAATAAACAAAATC
23[232]25[231]	GCGCAGAGATATCAAAATTATTTGACATTATC
25[232]27[231]	ATTTTGCGTCTTTAGGAGCACTAAGCAACAGT
27[232]29[231]	GCCACGCTATACGTGGCACAGACAACGCTCAT
29[232]28[248]	GGAAATACCTACATTTTGACGCTCACCTGAAA
10[244]11[244]	TTTTCAAAAGGGCGACATTCATTTACCAGCGCCAAAGATTTT
12[247]13[231]	ACCCAAAAGAACTGGCATGATTAAAGCCGAAC
14[247]12[248]	CTGAACACCAGAAGGAAACCGAGGAACGGAAT
16[247]14[248]	AGGTTTTGAACGTCAAAAATGAAAAGAATTAA
18[247]16[248]	AATGCAGACCGTTTTTATTTTCATCTTGCGGG
20[247]18[248]	AATGGTTTACAACGCCAACATGTAGTTCAGCT
22[247]20[248]	AAATCAATGGCTTAGGTTGGGTTACTAAATTT
24[247]22[248]	AACCTACCGCGAATTATTCATTTCCAGTACAT
26[247]24[248]	CTAAAATAGAACAAAGAAACCACCAGGGTTAG
28[247]26[248]	GCGTAAGAGAGAGCCAGCAGCAAAAAGGTTAT
15[264]15[286]	CTTTACAGAGAGAATAACATTTT
17[264]19[263]	TCATTACCCGACAATAAACAACATATTTAGGC
19[264]21[263]	AGAGGCATAATTTCATCTTCTGACTATAACTA
21[264]23[263]	TATGTAAACCTTTTTTAATGGAAAAATTACCT
23[264]25[263]	GAGCAAAAACTTCTGAATAATGGAAGAAGGAG
25[264]27[263]	CGGAATTATTGAAAGGAATTGAGGTGAAAAAT
27[264]29[263]	CTAAAGCAAGATAGAACCCTTCTGAATCGTCT
29[264]28[280]	GAAATGGATTATTTACATTGGCAGACATTCTG
12[265]13[265]	TTTTAATAATAAACGCTTTT
16[279]17[263]	GAGGCGTTTTAGCGAACCTCCCGACGTAGGAA
18[279]16[280]	CCAGACGAGCGCCCAATAGCAAGCAAGAACGC
20[279]18[280]	TTTTAGTTTTTCGAGCCAGTAATAAATTCTGT
22[279]20[280]	TTGAATTATGCTGATGCAAATCCACAAATATA
24[279]22[280]	TGGATTATGAAGATGATGAAACAAAATTTCAT
26[279]24[280]	ATCAACAGTCATCATATTCCTGATTGATTGTT
28[279]26[280]	GCCAACAGTCACCTTGCTGAACCTGTTGGCAA
14[286]15[263]	TTTTTAAAAACAGGGAAGCGCATTAGACGGGATAGCAGC
16[307]17[307]	TTTTGCTTATCCGGTATTCTAAATCAGATATAGAAGTTTT
18[307]19[307]	TTTTCGACAAAAGGTAAAGTAGAGAATATAAAGTACTTTT
20[307]21[307]	TTTTCGCGAGAAAACTTTTTATCGCAAGACAAAGAATTTT
22[307]23[307]	TTTTATTAATTACATTTAACACATCAAGAAAACAAATTTT
24[307]25[307]	TTTTTTCATCAATATAATCCTATCAGATGATGGCAATTTT
26[307]27[307]	TTTTAATCAATATCTGGTCACAAATATCAAACCCTCTTTT

28[307]29[307]	TTTTACCAGTAATAAAAGGGATTCACCAGTCACACGTTTT

Table S2. Staple sequences for the truncated rectangle origami used in figures 2, 3, 4, and 5. The staple name refers to the starting helix[nucleotide] and the ending helix[nucleotide].

Rectangle Origami tile (Figures 4, 5)

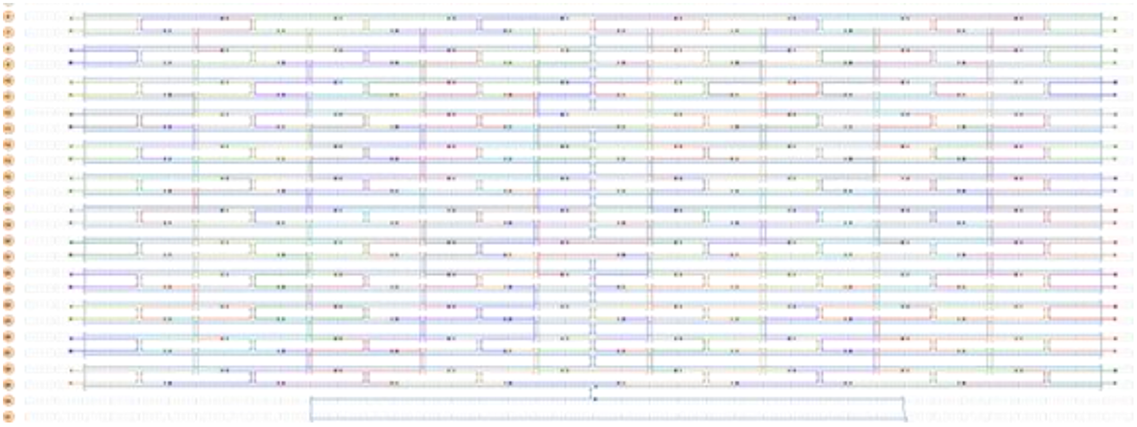

Figure S3. CaDNAno schematic of the rectangle origami used in figures 4 and 5.

Rectangle Origami Staples

7[12]6[12]	TTTTAACTTTCAACAGTTTCTGGGATTTTGCTAAACTTTT
9[12]8[12]	TTTTGACAGCATCGGAACGAACCCTCAGCAGCGAAATTTT
11[12]10[12]	TTTTTCATAAGGGAACCGAAAGGCGCAGACGGTCAATTTT
13[12]12[12]	TTTTGGAAGAAAAATCTACGACCAGTCAGGACGTTGTTTT
15[12]14[12]	TTTTCATTGAATCCCCCTCAAATCGTCATAAATATTTTTT
17[12]16[12]	TTTTCTGTAGCTCAACATGTATTGCTGAATATAATGTTTT
19[12]18[12]	TTTTGGTTGTACCAAAAACAAGCATAAAGCTAAATCTTTT
21[12]20[12]	TTTTGATGAACGGTAATCGTAGCAAACAAGAGAATCTTTT
23[12]22[12]	TTTTGTAATGGGATAGGTCAAAACGGCGGATTGACCTTTT
25[12]24[12]	TTTTTGTAAAACGACGGCCATTCCCAGTCACGACGTTTTT
27[12]26[12]	TTTTCGGTTTGCGTATTGGGAACGCGCGGGGAGAGGTTTT
29[12]28[12]	TTTTCGATGGCCCACTACGTAAACCGTCTATCAGGGTTTT
7[40]9[39]	GAGAATAGCTTTTGCGGGATCGTCGGGTAGCA
9[40]11[39]	ACGGCTACTTACTTAGCCGGAACGCTGACCAA
11[40]13[39]	CTTTGAAAAGAACTGGCTCATTATTTAATAAA
13[40]15[39]	ACGAACTAGCGTCCAATACTGCGGAATGCTTT
15[40]17[39]	AAACAGTTGATGGCTTAGAGCTTATTTAAATA
17[40]19[39]	TGCAACTAAGCAATAAAGCCTCAGTTATGACC
19[40]21[39]	CTGTAATATTGCCTGAGAGTCTGGAAAACTAG
21[40]23[39]	CATGTCAAGATTCTCCGTGGGAACCGTTGGTG
23[40]25[39]	TAGATGGGGGGTAACGCCAGGGTTGTGCCAAG
25[40]27[39]	CTTGCATGCATTAATGAATCGGCCCGCCAGGG
27[40]29[39]	TGGTTTTTAACGTCAAAGGGCGAAGAACCATC
29[40]28[56]	ACCCAAATCAAGTTTTTTGGGGTCAAAGAACG
6[55]7[39]	ACGTTAGTAAATGAATTTTCTGTAAGCGGAGT
8[55]6[56]	AAAGGCCGAAAGGAACAACTAAAGCTTTCCAG
10[55]8[56]	GCTCCATGAGAGGCTTTGAGGACTAGGGAGTT
12[55]10[56]	CGATTTTAGAGGACAGATGAACGGCGCGACCT
14[55]12[56]	ACTGGATAACGGAACAACATTATTACCTTATG
16[55]14[56]	TTTTTGCGCAGAAAACGAGAATGAATGTTTAG
18[55]16[56]	CAAAATTAAAGTACGGTGTCTGGAAGAGGTCA
20[55]18[56]	TCAGGTCACTTTTGCGGGAGAAGCAGAATTAG
22[55]20[56]	ACCCGTCGTCATATGTACCCCGGTAAAGGCTA
24[55]22[56]	ATTAAGTTCGCATCGTAACCGTGCGAGTAACA
26[55]24[56]	GCCAGCTGCCTGCAGGTCGACTCTGCAAGGCG
28[55]26[56]	TGGACTCCCTTTTCACCAGTGAGACCTGTCGT
7[72]9[71]	AATAATAAGGTCGCTGAGGCTTGCAAAGACTT
9[72]11[71]	TTTCATGAAAATTGTGTCGAAATCTGTACAGA

11[72]13[71]	CCAGGCGCTTAATCATTGTGAATTACAGGTAG
13[72]15[71]	AAAGATTCAGGGGGTAATAGTAAACCATAAAT
15[72]17[71]	CAAAAATCATTGCTCCTTTTGATAAGTTTCAT
17[72]19[71]	TCCATATACATACAGGCAAGGCAACTTTATTT
19[72]21[71]	CAACGCAATTTTTGAGAGATCTACTGATAATC
21[72]23[71]	AGAAAAGCAACATTAAATGTGAGCATCTGCCA
23[72]25[71]	GTTTGAGGGAAAGGGGGATGTGCTAGAGGATC
25[72]27[71]	CCCGGGTACTTTCCAGTCGGGAAACGGGCAAC
27[72]29[71]	AGCTGATTACAAGAGTCCACTATTGAGGTGCC
29[72]28[88]	GTAAAGCACTAAATCGGAACCCTAGTTGTTCC
6[87]7[71]	CGTAACGATCTAAAGTTTTGTCGTGAATTGCG
8[87]6[88]	ATATATTCTTTTTTCACGTTGAAAATAGTTAG
10[87]8[88]	CGCCTGATGGAAGTTTCCATTAAACATAACCG
12[87]10[88]	TTTCAACTATAGGCTGGCTGACCTTGTATCAT
14[87]12[88]	TTTGCCAGATCAGTTGAGATTTAGTGGTTTAA
16[87]14[88]	TACCTTTAAGGTCTTTACCCTGACAAAGAAGT
18[87]16[88]	CAATAAATACAGTTGATTCCCAATTTAGAGAG
20[87]18[88]	GGTAGCTAGGATAAAAATTTTTAGTTAACATC
22[87]20[88]	CTTTCATCCCCAAAAACAGGAAGACCGGAGAG
24[87]22[88]	CAGCTGGCGGACGACGACAGTATCGTAGCCAG
26[87]24[88]	ACTGCCCGCCGAGCTCGAATTCGTTATTACGC
28[87]26[88]	AGTTTGGAGCCCTTCACCGCCTGGTTGCGCTC
7[104]9[103]	AAAAAAGGACAACCATCGCCCACGCGGGTAAA
9[104]11[103]	ATACGTAAAAGTACAACGGAGATTTCATCAAG
11[104]13[103]	AGTAATCTTAAATTGGGCTTGAGAGAATACCA
13[104]15[103]	CATTCAACGCGAGAGGCTTTTGCATATTATAG
15[104]17[103]	TCAGAAGCCTCCAACAGGTCAGGATCTGCGAA
17[104]19[103]	CGAGTAGAACTAATAGTAGTAGCAAACCCTCA
19[104]21[103]	TATATTTTAGCTGATAAATTAATGTTGTATAA
21[104]23[103]	GCAAATATCGCGTCTGGCCTTCCTGGCCTCAG
23[104]25[103]	GAAGATCGGTGCGGGCCTCTTCGCAATCATGG
25[104]27[103]	TCATAGCTACTCACATTAATTGCGCCCTGAGA
27[104]29[103]	GAGTTGCACGAGATAGGGTTGAGTAAGGGAGC
29[104]28[120]	CCCCGATTTAGAGCTTGACGGGGAAATCAAAA
6[119]7[103]	TGTAGCATTCCACAGACAGCCCTCATCTCCAA
8[119]6[120]	CAATGACACTCCAAAAGGAGCCTTACAACGCC
10[119]8[120]	GCGAAACATGCCACTACGAAGGCATGCGCCGA
12[119]10[120]	ACGAGTAGTGACAAGAACCGGATATACCAAGC
14[119]12[120]	CCAAAATATAATGCAGATACATAAACACCAGA
16[119]14[120]	GAAGCAAAAAAGCGGATTGCATCAGATAAAAA
18[119]16[120]	TCAATTCTTTTAGTTTGACCATTACCAGACCG
20[119]18[120]	ACCGTTCTAAATGCAATGCCTGAGAGGTGGCA
22[119]20[120]	AAATAATTTTAAATTGTAAACGTTGATATTCA
24[119]22[120]	GGCGATCGCACTCCAGCCAGCTTTGCCATCAA
26[119]24[120]	GTGAGCTAGTTTCCTGTGTGAAATTTGGGAAG
28[119]26[120]	GAATAGCCGCAAGCGGTCCACGCTCCTAATGA
7[136]9[135]	TCGGTTTAGCTTGATACCGATAGTCCAACCTA
9[136]11[135]	AAACGAAATGACCCCCAGCGATTATTCATTAC
11[136]13[135]	CCAAATCACTTGCCCTGACGAGAACGCCAAAA
13[136]15[135]	GGAATTACTCGTTTACCAGACGACAAAAGATT
15[136]17[135]	AAGAGGAACGAGCTTCAAAGCGAAGATACATT
17[136]19[135]	TCGCAAATGGGGCGCGAGCTGAAATAATGTGT
19[136]21[135]	AGGTAAAGAAATCACCATCAATATAATATTTT
21[136]23[135]	GTTAAAATTTTAACCAATAGGAACCCGGCACC
23[136]25[135]	GCTTCTGGTCAGGCTGCGCAACTGTGTTATCC
25[136]27[135]	GCTCACAATGTAAAGCCTGGGGTGGGTTTGCC
27[136]29[135]	CCAGCAGGGGCAAAATCCCTTATAAAGCCGGC
29[136]29[167]	GAACGTGGCGAGAAAGGAAGGGAACAAACTAT
6[151]7[135]	TGAGTTTCGTCACCAGTACAAACTTAATTGTA
8[151]6[152]	CTTAAACATCAGCTTGCTTTCGAGCGTAACAC
10[151]8[152]	CTCATCTTGAGGCAAAAGAATACAGTGAATTT
12[151]10[152]	GAATAAGGACGTAACAAAGCTGCTCTAAAACA
14[151]13[167]	CATAACCCGAGGCATAGTAAGAGCTTTTTAAG
16[151]14[152]	TTTTAATTGCCCGAAAGACTTCAAAACACTAT
18[151]16[152]	TTTCATTTGGTCAATAACCTGTTTATATCGCG
20[151]18[152]	AGACAGTCATTCAAAAGGGTGAGAAGCTATAT

22[151]21[167]	GCTCATTTTCGCATTAAATTTTTGAGCTTAGA
24[151]22[152]	TTCGCCATTGCCGGAAACCAGGCATTAAATCA
26[151]24[152]	GCATAAAGTTCCACACAACATACGAAGCGCCA
28[151]26[152]	CCGAAATCCGAAAATCCTGTTTGAAGCCGGAA
7[168]9[167]	AATGCCCCGTAACAGTGCCCGTATCTCCCTCA
9[168]11[167]	GAGCCGCCCCACCACCGGAACCGCGACGGAAA
11[168]12[152]	TTATTCATAGGGAAGGTAAATATTCATTCAGT
13[168]15[167]	AAAAGTAATATCTTACCGAAGCCCTTCCAGAG
15[168]17[167]	CCTAATTTACGCTAACGAGCGTCTAATCAATA
17[168]19[167]	ATCGGCTGCGAGCATGTAGAAACCTATCATAT
19[168]20[152]	GCGTTATAGAAAAAGCCTGTTTAGAAGGCCGG
21[168]23[167]	TTAAGACGTTGAAAACATAGCGATAACAGTAC
23[168]25[167]	CTTTTACACAGATGAATATACAGTAAACAATT
25[168]27[167]	CGACAACTAAGTATTAGACTTTACAATACCGA
27[168]28[152]	ACGAACCAAAACATCGCCATTAAATGGTGGTT
29[168]28[184]	CGGCCTTGCTGGTAATATCCAGAACGAACTGA
6[183]7[167]	CAAGCCCAATAGGAACCCATGTACAAACAGTT
8[183]6[184]	TGCCTTGACTGCCTATTTCGGAACAGGGATAG
10[183]8[184]	AACCAGAGACCCTCAGAACCGCCAGGGGTCAG
12[183]10[184]	ATTGAGGGTAAAGGTGAATTATCAATCACCGG
14[183]12[184]	GCAATAGCGCAGATAGCCGAACAATTCAACCG
16[183]14[184]	TCTTACCAGCCAGTTACAAAATAAATGAAATA
18[183]16[184]	CTAATTTATCTTTCCTTATCATTCATCCTGAA
20[183]18[184]	AATTACTACAAATTCTTACCAGTAATCCCATC
22[183]20[184]	TAGAATCCCTGAGAAGAGTCAATAGGAATCAT
24[183]22[184]	TTTAACGTTCGGGAGAAACAATAATTTTCCCT
26[183]24[184]	GGATTTAGCGTATTAAATCCTTTGTTTTCAGG
28[183]26[184]	TAGCCCTACCAGCAGAAGATAAAAACATTTGA
7[200]9[199]	CTGAAACAGGTAATAAGTTTTAACCCCTCAGA
9[200]11[199]	GCCACCACTCTTTTCATAATCAAACCGTCACC
11[200]13[199]	GACTTGAGAGACAAAAGGGCGACAAGTTACCA
13[200]15[199]	GAAGGAAAATAAGAGCAAGAAACAACAGCCAT
15[200]17[199]	ATTATTTAACCCAGCTACAATTTTCAAGAACG
17[200]19[199]	GGTATTAAGAACAAGAAAAATAATTAAAGCCA
19[200]21[199]	ACGCTCAAAATAAGAATAAACACCGTGAATTT
21[200]23[199]	ATCAAAATCGTCGCTATTAATTAACGGATTCG
23[200]25[199]	CCTGATTGAAAGAAATTGCGTAGACCCGAACG
25[200]27[199]	TTATTAATGCCGTCAATAGATAATCAGAGGTG
27[200]29[199]	AGGCGGTCATTAGTCTTTAATGCGCAATATTA
29[200]28[216]	CCGCCAGCCATTGCAACAGGAAAAATATTTTT
6[215]7[199]	CTCAGAGCCACCACCCTCATTTTCCTATTATT
8[215]6[216]	AGTGTACTTGAAAGTATTAAGAGGCCGCCACC
10[215]8[216]	GTTTGCCACCTCAGAGCCGCCACCGATACAGG
12[215]10[216]	AGCGCCAACCATTTGGGAATTAGATTATTAGC
14[215]12[216]	GCCCAATACCGAGGAAACGCAATAGGTTTACC
16[215]14[216]	TATTTTGCTCCCAATCCAAATAAGTGAGTTAA
18[215]16[216]	TAAGTCCTACCAAGTACCGCACTCTTAGTTGC
20[215]18[216]	AGGCGTTACAGTAGGGCTTAATTGACAATAGA
22[215]20[216]	CTGTAAATCATAGGTCTGAGAGACGATAAATA
24[215]22[216]	ACAGAAATCTTTGAATACCAAGTTCCTTGCTT
26[215]24[216]	AGATTAGATTTAAAAGTTTGAGTACACGTAAA
28[215]26[216]	GAATGGCTAGTATTAACACCGCCTCAACTAAT
7[232]9[231]	CCTCAAGAATACATGGCTTTTGATAGAACCAC
9[232]11[231]	CACCAGAGTTCGGTCATAGCCCCCGCCAGCAA
11[232]13[231]	AATCACCAAATAGAAAATTCATATATAACGGA
13[232]15[231]	ATACCCAAGATAACCCACAAGAATAAACGATT
15[232]17[231]	TTTTGTTTAAGCCTTAAATCAAGAATCGAGAA
17[232]19[231]	CAAGCAAGACGCGCCTGTTTATCAAGAATCGC
19[232]21[231]	CATATTTAGAAATACCGACCGTGTTACCTTTT
21[232]23[231]	TAACCTCCATATGTGAGTGAATAAACAAAATC
23[232]25[231]	GCGCAGAGATATCAAAATTATTTGACATTATC
25[232]27[231]	ATTTTGCGTCTTTAGGAGCACTAAGCAACAGT
27[232]29[231]	GCCACGCTATACGTGGCACAGACAACGCTCAT
29[232]28[248]	GGAAATACCTACATTTTGACGCTCACCTGAAA
6[247]7[231]	CCCTCAGAACCGCCACCCTCAGAACTGAGACT
8[247]6[248]	TAAGCGTCGAAGGATTAGGATTAGTACCGCCA

10[247]8[248]	TCGGCATTCCGCCGCCAGCATTGACGTTCCAG
12[247]10[248]	TCACAATCGTAGCACCATTACCATCGTTTTCA
14[247]12[248]	ATCAGAGAAAGAACTGGCATGATTTTATTTTG
16[247]14[248]	AGGTTTTGAACGTCAAAAATGAAAGCGCTAAT
18[247]16[248]	AATGCAGACCGTTTTTATTTTCATCTTGCGGG
20[247]18[248]	AATGGTTTACAACGCCAACATGTAGTTCAGCT
22[247]20[248]	AAATCAATGGCTTAGGTTGGGTTACTAAATTT
24[247]22[248]	AACCTACCGCGAATTATTCATTTCCAGTACAT
26[247]24[248]	CTAAAATAGAACAAAGAAACCACCAGGGTTAG
28[247]26[248]	GCGTAAGAGAGAGCCAGCAGCAAAAAGGTTAT
7[264]9[263]	TGCTCAGTCAGTCTCTGAATTTACCAGGAGGT
9[264]11[263]	TGAGGCAGGCGTCAGACTGTAGCGTAGCAAGG
11[264]13[263]	CCGGAAACACACCACGGAATAAGTAAGACTCC
13[264]15[263]	TTATTACGGTCAGAGGGTAATTGAATAGCAGC
15[264]17[263]	CTTTACAGTTAGCGAACCTCCCGACGTAGGAA
17[264]19[263]	TCATTACCCGACAATAAACAACATATTTAGGC
19[264]21[263]	AGAGGCATAATTTCATCTTCTGACTATAACTA
21[264]23[263]	TATGTAAACCTTTTTTAATGGAAAAATTACCT
23[264]25[263]	GAGCAAAAACTTCTGAATAATGGAAGAAGGAG
25[264]27[263]	CGGAATTATTGAAAGGAATTGAGGTGAAAAAT
27[264]29[263]	CTAAAGCAAGATAGAACCCTTCTGAATCGTCT
29[264]28[280]	GAAATGGATTATTTACATTGGCAGACATTCTG
6[279]7[263]	TATCACCGTACTCAGGAGGTTTAGCGGGGTTT
8[279]6[280]	GGAAAGCGACCAGGCGGATAAGTGAATAGGTG
10[279]8[280]	TGCCTTTAGTCAGACGATTGGCCTGCCAGAAT
12[279]10[280]	ACGCAAAGGTCACCAATGAAACCAATCAAGTT
14[279]12[280]	TGAACAAACAGTATGTTAGCAAACTAAAAGAA
16[279]14[280]	GAGGCGTTAGAGAATAACATAAAAGAACACCC
18[279]16[280]	CCAGACGAGCGCCCAATAGCAAGCAAGAACGC
20[279]18[280]	TTTTAGTTTTTCGAGCCAGTAATAAATTCTGT
22[279]20[280]	TTGAATTATGCTGATGCAAATCCACAAATATA
24[279]22[280]	TGGATTATGAAGATGATGAAACAAAATTTCAT
26[279]24[280]	ATCAACAGTCATCATATTCCTGATTGATTGTT
28[279]26[280]	GCCAACAGTCACCTTGCTGAACCTGTTGGCAA
6[307]7[307]	TTTTTATAAGTATAGCCCGGCCGTCGAGAGGGTTGATTTT
8[307]9[307]	TTTTATAAATCCTCATTAAATGATATTCACAAACAATTTT
10[307]11[307]	TTTTAATCAGTAGCGACAGATCGATAGCAGCACCGTTTTT
12[307]13[307]	TTTTTAAAGGTGGCAACATAGTAGAAAATACATACATTTT
14[307]15[307]	TTTTGACGGGAGAATTAACTACAGGGAAGCGCATTATTTT
16[307]17[307]	TTTTGCTTATCCGGTATTCTAAATCAGATATAGAAGTTTT
18[307]19[307]	TTTTCGACAAAAGGTAAAGTAGAGAATATAAAGTACTTTT
20[307]21[307]	TTTTCGCGAGAAAACTTTTTATCGCAAGACAAAGAATTTT
22[307]23[307]	TTTTATTAATTACATTTAACACATCAAGAAAACAAATTTT
24[307]25[307]	TTTTTTCATCAATATAATCCTATCAGATGATGGCAATTTT
26[307]27[307]	TTTTAATCAATATCTGGTCACAAATATCAAACCCTCTTTT
28[307]29[307]	TTTTACCAGTAATAAAAGGGATTCACCAGTCACACGTTTT

Table S3. Staple sequences for the rectangular origami used in figures 4 and 5. The staple name refers to the starting helix[nucleotide] and the ending helix[nucleotide].

CanDo Models of origami designs

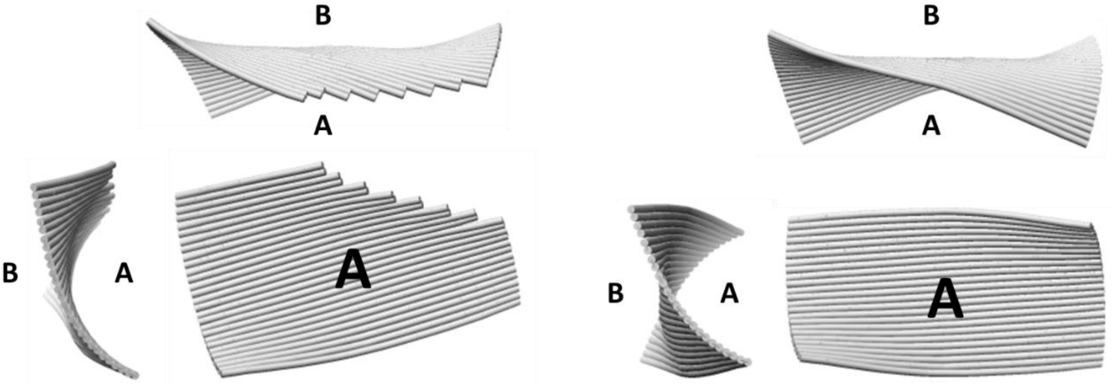

Figure S4. Cando models of the origami used in this work. The curvature and twisted structure arises from the inability to place a non-integer number of bases between crossovers such that the rotational offset between crossover points would conform to an integer DNA number of full-DNA turn. The orientations (i.e. A and B faces) of each design are highlighted

Schematics for origami modified edges

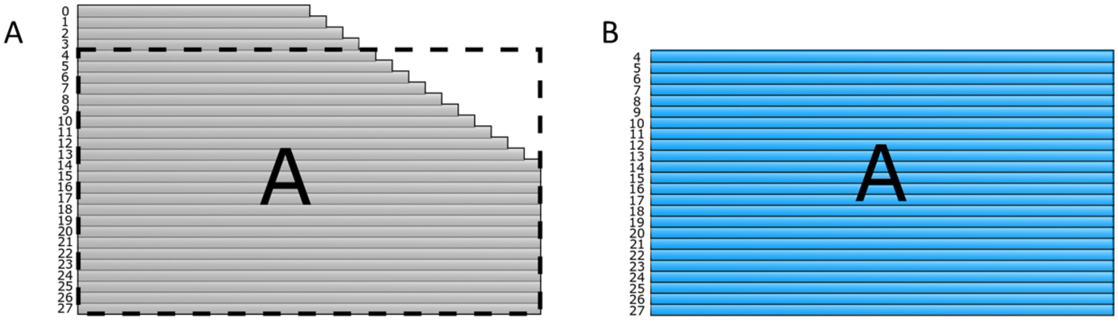

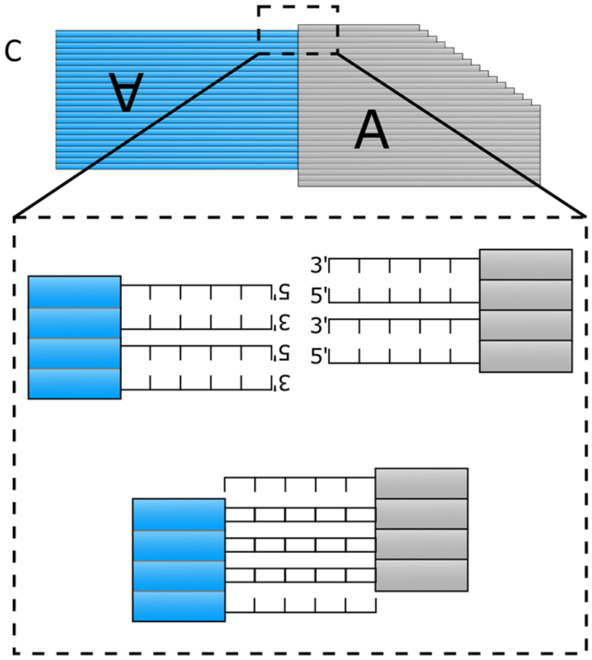

D

	V dimer		A dimer	
		T	A	0
1	N	nt	A	1
2	N	nt	B	2
3	M	nt	B	3
4	M	nt	C	4
5	L	nt	C	5
6	L	nt	D	6
7	K	nt	D	7
8	K	nt	E	8
9	J	nt	E	9
10	J	nt	F	10
11	I	nt	F	11
**12**	I	nt	G	**12**
**13**	H	nt	G	**13**
14	H	nt	H	14
15	G	nt	H	15
16	G	nt	I	16
17	F	nt	I	17
18	F	nt	J	18
19	E	nt	J	19
20	E	nt	K	20
21	D	nt	K	21
22	D	nt	L	22
23	C	nt	L	23
24	C	nt	M	24
25	B	T	M	25
26	B	T	N	26
27	A	T	N	27
	A	T		

Figure S5. Orientation of origami tiles when assembled into dimers, showing the naming structure/groups for the staple strands. Schematics of the origami used in this work, showing the number of helices in each. The dashed line in A) denotes the area of the origami design in B). C) Due to the underlying staple spacing, and as seen in the CanDo models, both tiles exhibit a curved, non-planar structure. - to minimise the spatial mismatch when creating dimers, one tile is rotated 180° labelled here as '∀'. This creates a helix offset between the two designs at the interface. D) The edges of the two tiles share the same edge staple sequences (sequences are included below. The letters A-N included in the name of each staple indicate the position along the edge of the origami).

Modified-staples

Name	Sequence
A-pT	**TTTT**ATAAATCCTCATTAAATGATATTCACAAACAA**TTTT**
B-pT	**TTTT**TATAAGTATAGCCCGGCCGTCGAGAGGGTTGA**TTTT**
C-pT	**TTTT**AACTTTCAACAGTTTCTGGGATTTTGCTAAAC**TTTT**
D-pT	**TTTT**GACAGCATCGGAACGAACCCTCAGCAGCGAAA**TTTT**
E-pT	**TTTT**TCATAAGGGAACCGAAAGGCGCAGACGGTCAA**TTTT**
F-pT	**TTTT**GGAAGAAAAATCTACGACCAGTCAGGACGTTG**TTTT**
G-pT	**TTTT**CATTGAATCCCCCTCAAATCGTCATAAATATT**TTTT**
H-pT	**TTTT**CTGTAGCTCAACATGTATTGCTGAATATAATG**TTTT**
I-pT	**TTTT**GGTTGTACCAAAAACAAGCATAAAGCTAAATC**TTTT**
J-pT	**TTTT**GATGAACGGTAATCGTAGCAAACAAGAGAATC**TTTT**
K-pT	**TTTT**GTAATGGGATAGGTCAAAACGGCGGATTGACC**TTTT**
L-pT	**TTTT**TGTAAAACGACGGCCATTCCCAGTCACGACGT**TTTT**
M-pT	**TTTT**CGGTTTGCGTATTGGGAACGCGCGGGGAGAGG**TTTT**
N-pT	**TTTT**CGATGGCCCACTACGTAAACCGTCTATCAGGG**TTTT**

Table S4. poly-T terminated edge staples to prevent blunt-end stacking

Sequence	Name		Sequence
GTGTCATAAATCCTCATTAAATGATATTCACAAACAATTTT	A-1	∀-1-2	GACACCGATGGCCCACTACGTAAACCGTCTATCAGGGAGGAA
CGTACTATAAGTATAGCCCGGCCGTCGAGAGGGTTGATTCCT	A-3-2	∀-3-4	GTACGCGGTTTGCGTATTGGGAACGCGCGGGGAGAGGACGAA
CAGGTAACTTTCAACAGTTTCTGGGATTTTGCTAAACTTCGT	A-5-4	∀-5-6	ACCTGTGTAAAACGACGGCCATTCCCAGTCACGACGTGGTTA
GTACCGACAGCATCGGAACGAACCCTCAGCAGCGAAATAACC	A-7-6	∀-7-8	GGTACGTAATGGGATAGGTCAAAACGGCGGATTGACCAACGA
CACACTCATAAGGGAACCGAAAGGCGCAGACGGTCAATCGTT	A-9-8	∀-9-10	GTGTGGATGAACGGTAATCGTAGCAAACAAGAGAATCATCTG
TGCACGGAAGAAAAATCTACGACCAGTCAGGACGTTGCAGAT	A-11-10	∀-11-12	GTGCAGGTTGTACCAAAAACAAGCATAAAGCTAAATCTCTTG
TCTCCCATTGAATCCCCCTCAAATCGTCATAAATATTCAAGA	A-13-12	∀-13-14	GGAGACTGTAGCTCAACATGTATTGCTGAATATAATGCTCGT
GTATGCTGTAGCTCAACATGTATTGCTGAATATAATGACGAG	A-15-14	∀-15-16	CATACCATTGAATCCCCCTCAAATCGTCATAAATATTTCCTC
CTACAGGTTGTACCAAAAACAAGCATAAAGCTAAATCGAGGA	A-17-16	∀-17-18	TGTAGGGAAGAAAAATCTACGACCAGTCAGGACGTTGAGGAC
TTAGGGATGAACGGTAATCGTAGCAAACAAGAGAATCGTCCT	A-19-18	∀-19-20	CCTAATCATAAGGGAACCGAAAGGCGCAGACGGTCAAAGTGG
AGACAGTAATGGGATAGGTCAAAACGGCGGATTGACCCCACT	A-21-20	∀-21-22	TGTCTGACAGCATCGGAACGAACCCTCAGCAGCGAAAAGACG
TAGAGTGTAAAACGACGGCCATTCCCAGTCACGACGTCGTCT	A-23-22	∀-23-24	CTCTAAACTTTCAACAGTTTCTGGGATTTTGCTAAACACGCA
CATGACGGTTTGCGTATTGGGAACGCGCGGGGAGAGGTGCGT	A-25-24	∀-25-26	TCATGTATAAGTATAGCCCGGCCGTCGAGAGGGTTGATACGG
CCATTCGATGGCCCACTACGTAAACCGTCTATCAGGGCCGTA	A-27-26	∀-27	AATGGATAAATCCTCATTAAATGATATTCACAAACAATTTT

Table S5. Modified (5' and 3') edge staples for sticky-end based linkages in dimer constructs. Complementary sticky-ends used to join the A and ∀ origami tiles have corresponding colour/formatting.

Sequence	Name		Sequence
**GTGTC**ATAAATCCTCATTAAATGATATTCACAAACAA**TTTT**	A-1	∀-1	**GACAC**CGATGGCCCACTACGTAAACCGTCTATCAGGG**TTTT**
**CGTAC**TATAAGTATAGCCCGGCCGTCGAGAGGGTTGA**TTTT**	A-3	∀-3	**GTACG**CGGTTTGCGTATTGGGAACGCGCGGGGAGAGG**TTTT**
**CAGGT**AACTTTCAACAGTTTCTGGGATTTTGCTAAAC**TTTT**	A-5	∀-5	**ACCTG**TGTAAAACGACGGCCATTCCCAGTCACGACGT**TTTT**
**GTACC**GACAGCATCGGAACGAACCCTCAGCAGCGAAA**TTTT**	A-7	∀-7	**GGTAC**GTAATGGGATAGGTCAAAACGGCGGATTGACC**TTTT**
**CACAC**TCATAAGGGAACCGAAAGGCGCAGACGGTCAA**TTTT**	A-9	∀-9	**GTGTG**GATGAACGGTAATCGTAGCAAACAAGAGAATC**TTTT**

*TGCAC*GGAAGAAAAATCTACGACCAG TCAGGACGTTG**TTTT**	A-11	∀-11	*GTGCA*GGTTGTACCAAAAACAAGCATAAA GCTAAATC**TTTT**
*TCTCC*CATTGAATCCCCCTCAAATCG TCATAAATATT**TTTT**	A-13	∀-13	*GGAGA*CTGTAGCTCAACATGTATTGCTGA ATATAATG**TTTT**
*GTATG*CTGTAGCTCAACATGTATTGC TGAATATAATG**TTTT**	A-15	∀-15	*CATAC*CATTGAATCCCCCTCAAATCGTCA TAAATATT**TTTT**
CTACAGGTTGTACCAAAAACAAGCAT AAAGCTAAATC**TTTT**	A-17	∀-17	TGTAGGGAAGAAAAATCTACGACCAGTCA GGACGTTG**TTTT**
**TTAGG**GATGAACGGTAATCGTAGCAA ACAAGAGAATC**TTTT**	A-19	∀-19	**CCTAA**TCATAAGGGAACCGAAAGGCGCAG ACGGTCAA**TTTT**
**AGACA**GTAATGGGATAGGTCAAAACG GCGGATTGACC**TTTT**	A-21	∀-21	**TGTCT**GACAGCATCGGAACGAACCCTCAG CAGCGAAA**TTTT**
**TAGAG**TGTAAAACGACGGCCATTCCC AGTCACGACGT**TTTT**	A-23	∀-23	**CTCTA**AACTTTCAACAGTTTCTGGGATTT TGCTAAAC**TTTT**
**CATGA**CGGTTTGCGTATTGGGAACGC GCGGGGAGAGG**TTTT**	A-25	∀-25	**TCATG**TATAAGTATAGCCCGGCCGTCGAG AGGGTTGA**TTTT**
**CCATT**CGATGGCCCACTACGTAAACC GTCTATCAGGG**TTTT**	A-27	∀-27	**AATGG**ATAAATCCTCATTAAATGATATTC ACAAACAA**TTTT**

Table S6. Modified (5' only) edge staples for sticky-end based linkages in dimer constructs. 3' ends are modified with poly-T to prevent blunt-end stacking. Complementary sticky-ends used to join the A and ∀ origami tiles have corresponding colour/formatting.

Sequence	Name		Sequence
**TTTTTATAAGTATAGCCCGGCCGTCG AGAGGGTTGA**TTCCT	A-2	∀-2	**TTTT**CGATGGCCCACTACGTAAACCGTC TATCAGGG**AGGAA**
**TTTTAACTTTCAACAGTTTCTGGGAT TTTGCTAAAC**TTCGT	A-4	∀-4	**TTTT**CGGTTTGCGTATTGGGAACGCGCG GGGAGAGG**ACGAA**
**TTTTGACAGCATCGGAACGAACCCTC AGCAGCGAAA**TAACC	A-6	∀-6	**TTTT**TGTAAAACGACGGCCATTCCCAGT CACGACGT**GGTTA**
**TTTTTCATAAGGGAACCGAAAGGCGC AGACGGTCAA**TCGTT	A-8	∀-8	**TTTT**GTAATGGGATAGGTCAAAACGGCG GATTGACC**AACGA**
**TTTTGGAAGAAAAATCTACGACCAGT CAGGACGTTG**CAGAT	A-10	∀-10	**TTTT**GATGAACGGTAATCGTAGCAAACA AGAGAATC**ATCTG**
**TTTTCATTGAATCCCCCTCAAATCGT CATAAATATT***CAAGA*	A-12	∀-12	**TTTT**GGTTGTACCAAAAACAAGCATAAA GCTAAATC***TCTTG***
**TTTTCTGTAGCTCAACATGTATTGCT GAATATAATG***ACGAG*	A-14	∀-14	**TTTT**CTGTAGCTCAACATGTATTGCTGA ATATAATG***CTCGT***
**TTTTGGTTGTACCAAAAACAAGCATA AAGCTAAATC**GAGGA	A-16	∀-16	**TTTT**CATTGAATCCCCCTCAAATCGTCA TAAATATT**TCCTC**
**TTTTGATGAACGGTAATCGTAGCAAA CAAGAGAATC**GTCCT	A-18	∀-18	**TTTT**GGAAGAAAAATCTACGACCAGTCA GGACGTTG**AGGAC**
**TTTTGTAATGGGATAGGTCAAAACGG CGGATTGACC**CCACT	A-20	∀-20	**TTTT**TCATAAGGGAACCGAAAGGCGCAG ACGGTCAA**AGTGG**
**TTTTTGTAAAACGACGGCCATTCCCA GTCACGACGT**CGTCT	A-22	∀-22	**TTTT**GACAGCATCGGAACGAACCCTCAG CAGCGAAA**AGACG**
**TTTTCGGTTTGCGTATTGGGAACGCG CGGGGAGAGG**TGCGT	A-24	∀-24	**TTTT**AACTTTCAACAGTTTCTGGGATTT TGCTAAAC**ACGCA**
**TTTTCGATGGCCCACTACGTAAACCG TCTATCAGGG**CCGTA	A-26	∀-26	**TTTT**TATAAGTATAGCCCGGCCGTCGAG AGGGTTGA**TACGG**

Table S7. Modified (3' only) edge staples for sticky-end based linkages in dimer constructs. 5' ends are modified with poly-T to prevent blunt-end stacking. Complementary sticky-ends used to join the A and ∀ origami tiles have corresponding colour/formatting.

Fluorous ODNs and staples

Capture staples (figure 2)

Name	Sequence
**Capt_seq1_A**	TTTTATAAATCCTCATTAAATGATATTCACAAACAA**GCAGGACATCACAAAT**
**Capt_seq1_B**	TTTTTATAAGTATAGCCCGGCCGTCGAGAGGGTTGA**GCAGGACATCACAAAT**
**Capt_seq1_C**	TTTTAACTTTCAACAGTTTCTGGGATTTTGCTAAAC**GCAGGACATCACAAAT**
**Capt_seq1_D**	TTTTGACAGCATCGGAACGAACCCTCAGCAGCGAAA**GCAGGACATCACAAAT**

**Capt_seq1_E**	TTTTTCATAAGGGAACCGAAAGGCGCAGACGGTCAA**GCAGGACATCACAAAT**
**Capt_seq1_F**	TTTTGGAAGAAAAATCTACGACCAGTCAGGACGTTG**GCAGGACATCACAAAT**
**Capt_seq1_G**	TTTTCATTGAATCCCCCTCAAATCGTCATAAATATT**GCAGGACATCACAAAT**
**Capt_seq1_H**	TTTTCTGTAGCTCAACATGTATTGCTGAATATAATG**GCAGGACATCACAAAT**
**Capt_seq1_I**	TTTTGGTTGTACCAAAAACAAGCATAAAGCTAAATC**GCAGGACATCACAAAT**
**Capt_seq1_J**	TTTTGATGAACGGTAATCGTAGCAAACAAGAGAATC**GCAGGACATCACAAAT**
**Capt_seq1_K**	TTTTGTAATGGGATAGGTCAAAACGGCGGATTGACC**GCAGGACATCACAAAT**
**Capt_seq1_L**	TTTTTGTAAAACGACGGCCATTCCCAGTCACGACGT**GCAGGACATCACAAAT**
**Capt_seq1_M**	TTTTCGGTTTGCGTATTGGGAACGCGCGGGGAGAGG**GCAGGACATCACAAAT**
**Capt_seq1_N**	TTTTCGATGGCCCACTACGTAAACCGTCTATCAGGG**GCAGGACATCACAAAT**

Table S8. Modified (3') edge strands for use in the capture method shown in figure 2. Underlined sequences are complementary to modified ODNS shown in table S10 below (sequence 1)

Name	Sequence
**Capt_seq2_A**	TTTTATAAATCCTCATTAAATGATATTCACAAACAA**CTGCTATCTATCTGCA**
**Capt_seq2_B**	TTTTTATAAGTATAGCCCGGCCGTCGAGAGGGTTGA**CTGCTATCTATCTGCA**
**Capt_seq2_C**	TTTTAACTTTCAACAGTTTCTGGGATTTTGCTAAAC**CTGCTATCTATCTGCA**
**Capt_seq2_D**	TTTTGACAGCATCGGAACGAACCCTCAGCAGCGAAA**CTGCTATCTATCTGCA**
**Capt_seq2_E**	TTTTTCATAAGGGAACCGAAAGGCGCAGACGGTCAA**CTGCTATCTATCTGCA**
**Capt_seq2_F**	TTTTGGAAGAAAAATCTACGACCAGTCAGGACGTTG**CTGCTATCTATCTGCA**
**Capt_seq2_G**	TTTTCATTGAATCCCCCTCAAATCGTCATAAATATT**CTGCTATCTATCTGCA**
**Capt_seq2_H**	TTTTCTGTAGCTCAACATGTATTGCTGAATATAATG**CTGCTATCTATCTGCA**
**Capt_seq2_I**	TTTTGGTTGTACCAAAAACAAGCATAAAGCTAAATC**CTGCTATCTATCTGCA**
**Capt_seq2_J**	TTTTGATGAACGGTAATCGTAGCAAACAAGAGAATC**CTGCTATCTATCTGCA**
**Capt_seq2_K**	TTTTGTAATGGGATAGGTCAAAACGGCGGATTGACC**CTGCTATCTATCTGCA**
**Capt_seq2_L**	TTTTTGTAAAACGACGGCCATTCCCAGTCACGACGT**CTGCTATCTATCTGCA**
**Capt_seq2_M**	TTTTCGGTTTGCGTATTGGGAACGCGCGGGGAGAGG**CTGCTATCTATCTGCA**
**Capt_seq2_N**	TTTTCGATGGCCCACTACGTAAACCGTCTATCAGGG**CTGCTATCTATCTGCA**

Table S9. Modified (3') edge strands for use in the capture method shown in figure 2. Underlined sequences are complementary to modified ODNS shown in table S10 below (sequence 2)

Captured strands (figure 2)

5′ mod	DNA sequence	
$C_{10}H_{21}$	TGCAGATAGATAGCAG	
R_{F4}	TGCAGATAGATAGCAG	Sequence 1
R_{F6}	TGCAGATAGATAGCAG	
R_{F8}	ATTTGTGATGTCCTGC	
$(R_{F8})_2$	ATTTGTGATGTCCTGC	Sequence 2
$(R_{F8})_4$	ATTTGTGATGTCCTGC	

Table S10. Alkyl and fluorous ODNs used in figure 2. These sequences are complementary to the staples shown in tables S8 & S9.

Integrated R_F strands (figure 3, 4, 5)

Name	5′ mod	DNA sequence
**Int_(RF$_8$)$_2$_A**		ATAAATCCTCATTAAATGATATTCACAAACAATTTT
**Int_(RF$_8$)$_2$_B**		TATAAGTATAGCCCGGCCGTCGAGAGGGTTGATTTT
**Int_(RF$_8$)$_2$_C**		AACTTTCAACAGTTTCTGGGATTTTGCTAAACTTTT
**Int_(RF$_8$)$_2$_D**		GACAGCATCGGAACGAACCCTCAGCAGCGAAATTTT
**Int_(RF$_8$)$_2$_E**	$(R_{F8})_2$	TCATAAGGGAACCGAAAGGCGCAGACGGTCAATTTT
**Int_(RF$_8$)$_2$_F**		GGAAGAAAAATCTACGACCAGTCAGGACGTTGTTTT
**Int_(RF$_8$)$_2$_G**		CATTGAATCCCCCTCAAATCGTCATAAATATTTTTT
**Int_(RF$_8$)$_2$_H**		CTGTAGCTCAACATGTATTGCTGAATATAATGTTTT

**Int_$(RF_8)_2$_I**		GGTTGTACCAAAAACAAGCATAAAGCTAAATCTTTT
**Int_$(RF_8)_2$_J**		GATGAACGGTAATCGTAGCAAACAAGAGAATCTTTT
**Int_$(RF_8)_2$_K**		GTAATGGGATAGGTCAAAACGGCGGATTGACCTTTT
**Int_$(RF_8)_2$_L**		TGTAAAACGACGGCCATTCCCAGTCACGACGTTTTT
**Int_$(RF_8)_2$_M**		CGGTTTGCGTATTGGGAACGCGCGGGGAGAGGTTTT
**Int_$(RF_8)_2$_N**		CGATGGCCCACTACGTAAACCGTCTATCAGGGTTTT

Table S11. Fluorous modified (5') edge strands for use in the integrated method. 3' poly-T ends are used to prevent blunt-end stacking

**Name**	5′ mod	DNA sequence
**Int_ALKYL_A**		ATAAATCCTCATTAAATGATATTCACAAACAATTTT
**Int_ALKYL_B**		TATAAGTATAGCCCGGCCGTCGAGAGGGTTGATTTT
**Int_ALKYL_C**		AACTTTCAACAGTTTCTGGGATTTTGCTAAACTTTT
**Int_ALKYL_D**		GACAGCATCGGAACGAACCCTCAGCAGCGAAATTTT
**Int_ALKYL_E**		TCATAAGGGAACCGAAAGGCGCAGACGGTCAATTTT
**Int_ALKYL_F**		GGAAGAAAAATCTACGACCAGTCAGGACGTTGTTTT
**Int_ALKYL_G**		CATTGAATCCCCCTCAAATCGTCATAAATATTTTTT
**Int_ALKYL_H**	$C_{10}H_{21}$	CTGTAGCTCAACATGTATTGCTGAATATAATGTTTT
**Int_ALKYL_I**		GGTTGTACCAAAAACAAGCATAAAGCTAAATCTTTT
**Int_ALKYL_J**		GATGAACGGTAATCGTAGCAAACAAGAGAATCTTTT
**Int_ALKYL_K**		GTAATGGGATAGGTCAAAACGGCGGATTGACCTTTT
**Int_ALKYL_L**		TGTAAAACGACGGCCATTCCCAGTCACGACGTTTTT
**Int_ALKYL_M**		CGGTTTGCGTATTGGGAACGCGCGGGGAGAGGTTTT
**Int_ALKYL_N**		CGATGGCCCACTACGTAAACCGTCTATCAGGGTTTT

Table S12. Alkyl-modified (5') edge strands for use in the integrated method. 3' poly-T ends are used to prevent blunt-end stacking.

Additional AGE images

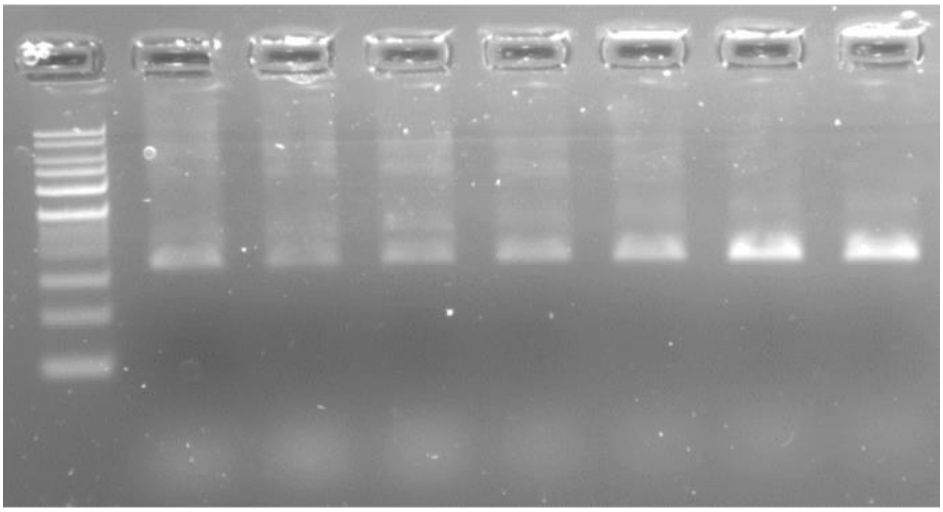

Figure S6. AGE image of truncated-origami modified to capture R_{F8}-ODNs. Lane 1 – ladder, Lanes 2-8 increasing R_{F8} strands (2, 4, 6, 8, 10, 12, 14, respectively).

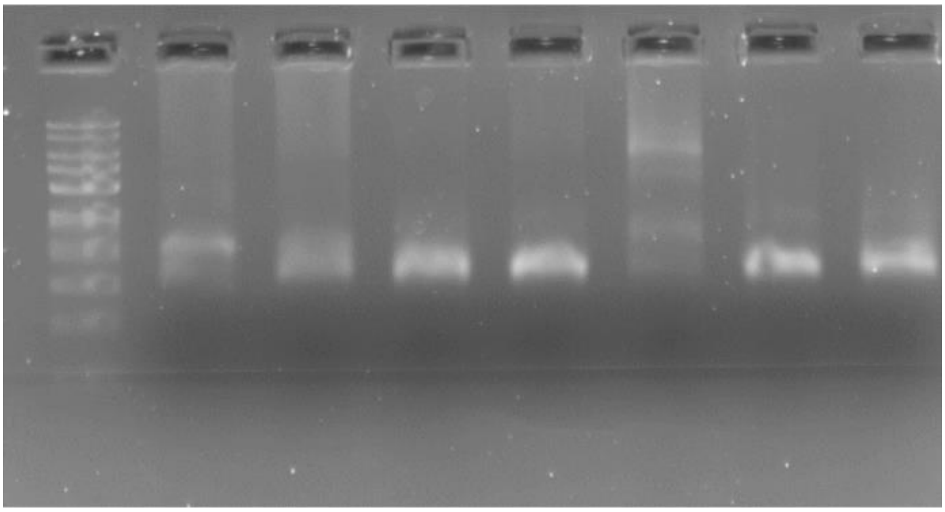

Figure S7. AGE image of truncated rectangle-origami modified to capture $(R_{F8})_4$-ODNs. Lane 1 – ladder, Lanes 2-8 increasing $(R_{F8})_4$ strands (2, 4, 6, 8, 10, 12, 14, respectively).

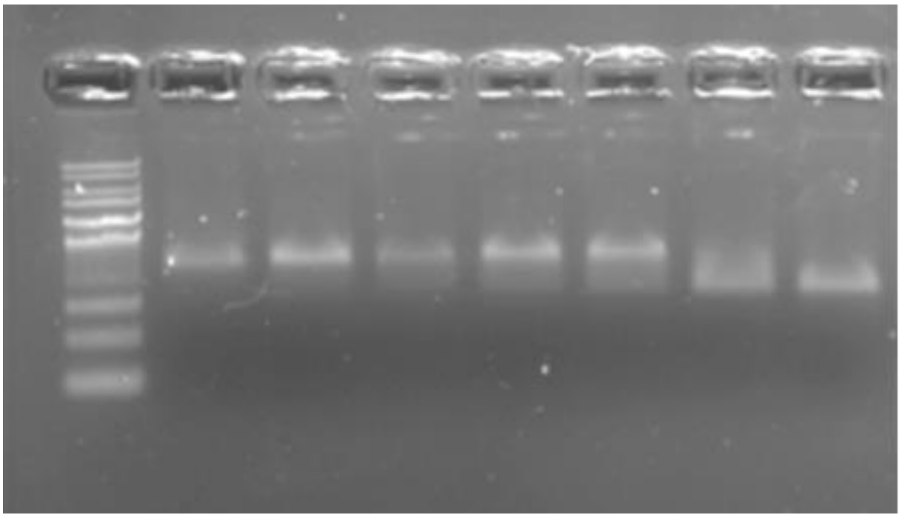

Figure S8. AGE image of heterodimer constructs with decreasing DNA overhangs. Lane 1 – ladder, Lanes 2-8 decreasing DNA overhangs (24, 20, 16, 12, 8, 4, 0, respectively).

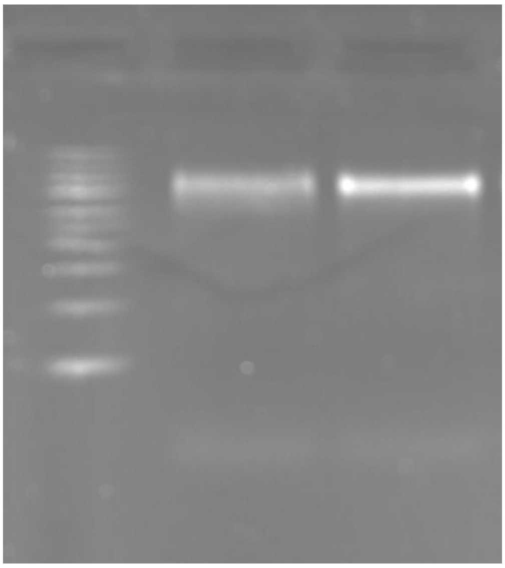

Figure S9. AGE image of truncated rectangle origami with edge modifications. Lane 1 – ladder, Lane 2 – 14 alkyl tags, Lane 3 – unmodified origami.

AFM images

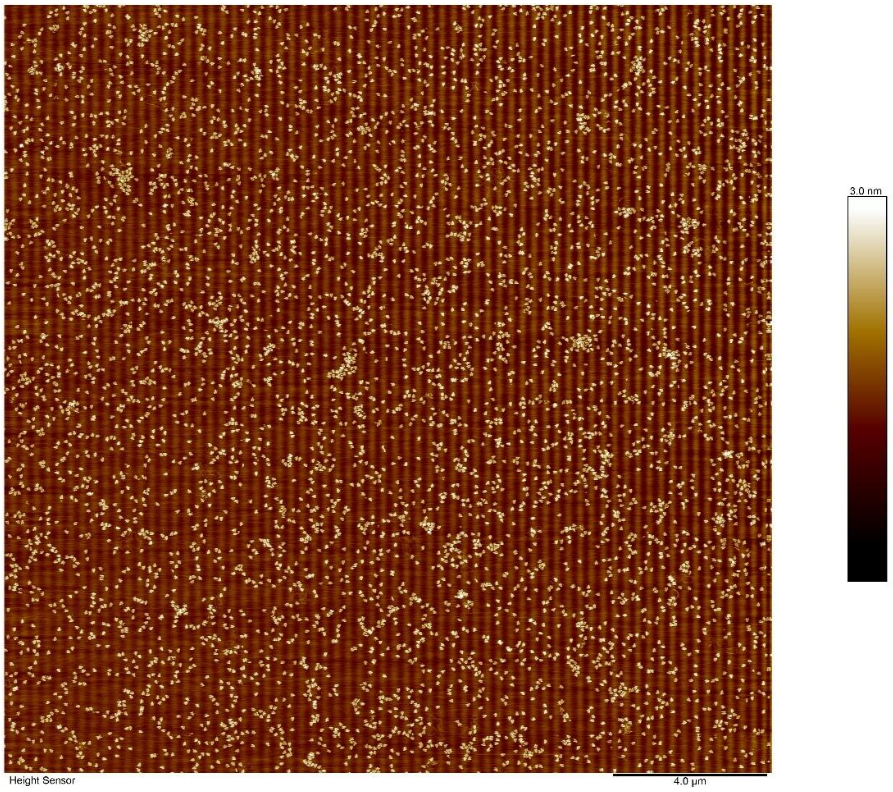

Figure S10. Truncated rectangle origami with 14 capture strands, capturing unmodified ODN

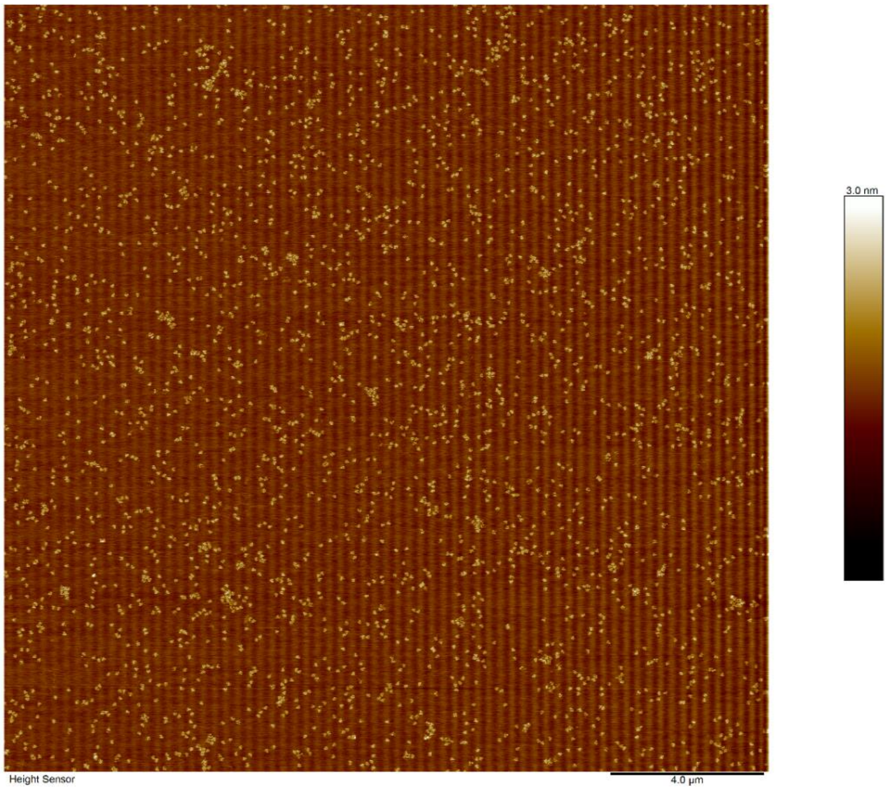

Figure S11. Truncated rectangle origami with 14 capture strands, capturing alkyl-ODN

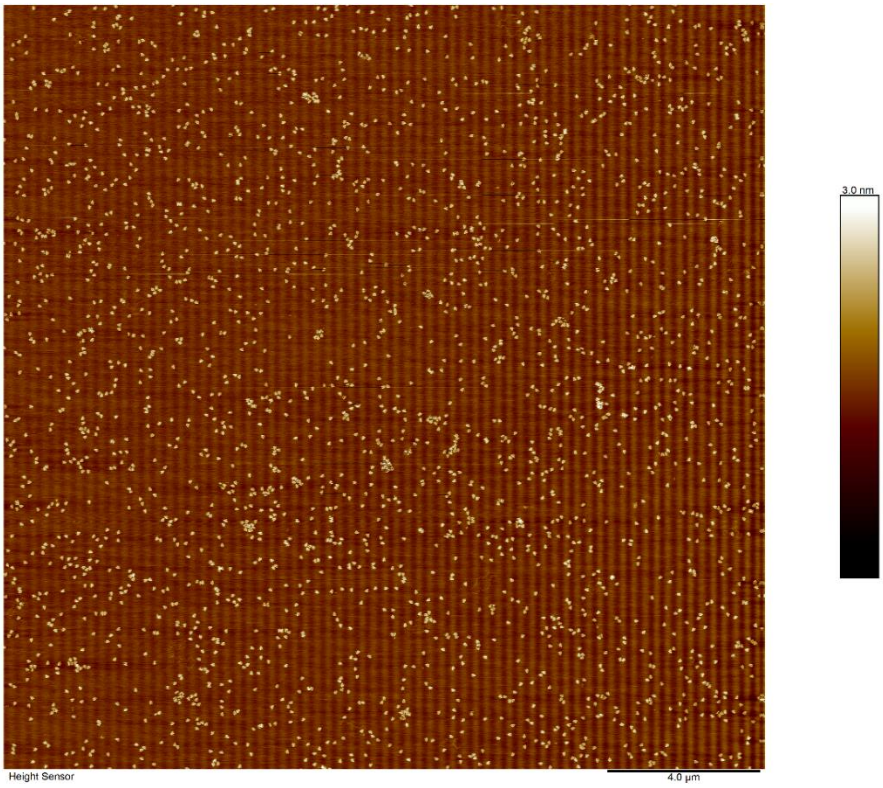

Figure S12. Truncated rectangle origami with 14 capture strands, capturing R_{F4}-ODNs

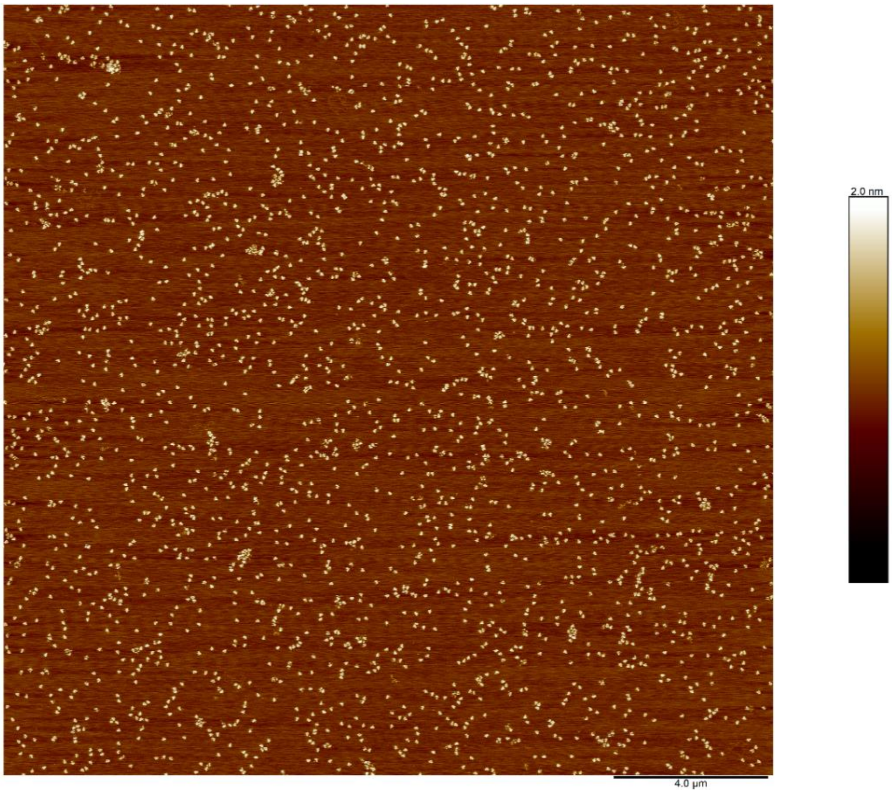

Figure S13. Truncated rectangle origami with 14 capture strands, capturing R_{F6}-ODNs

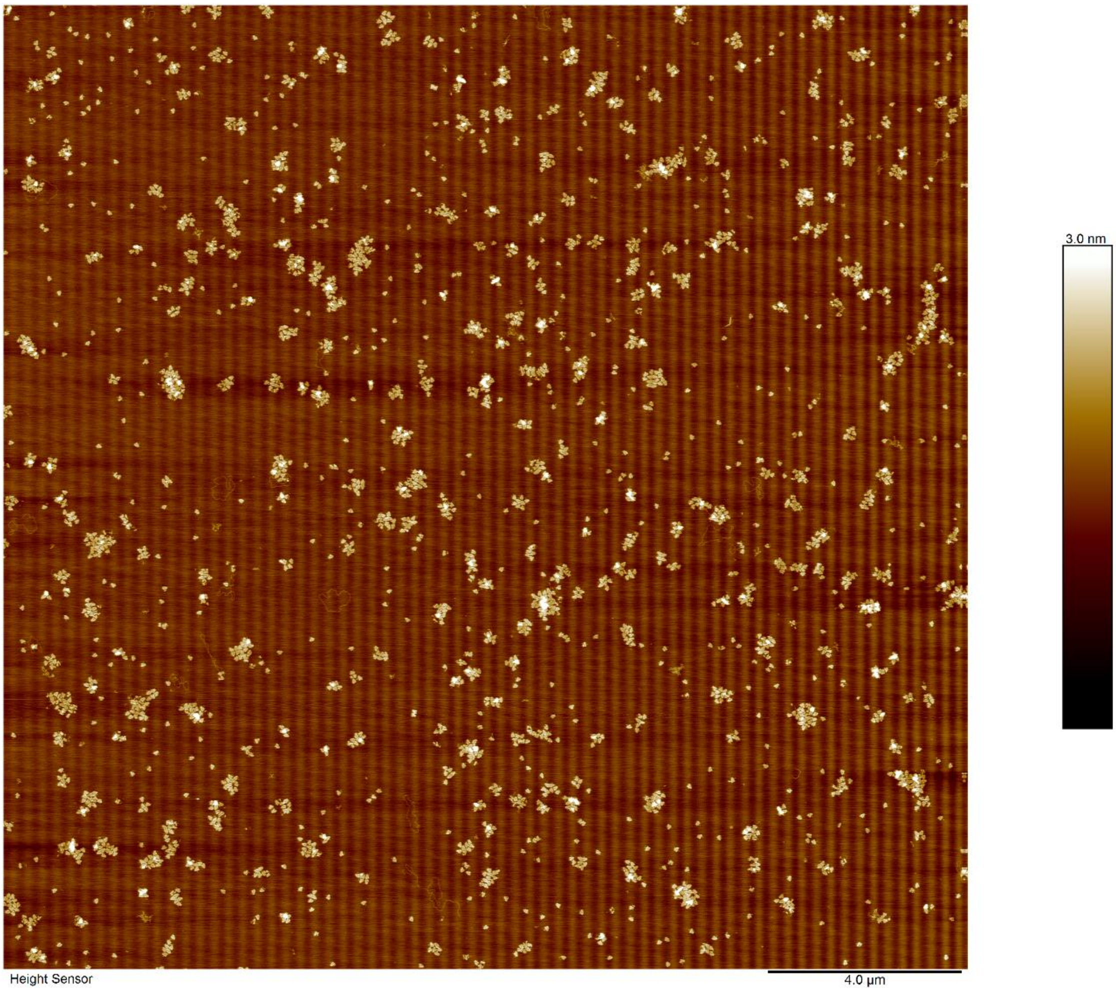

Figure S14. Truncated rectangle origami with 14 capture strands, capturing R_{F8}-ODNs

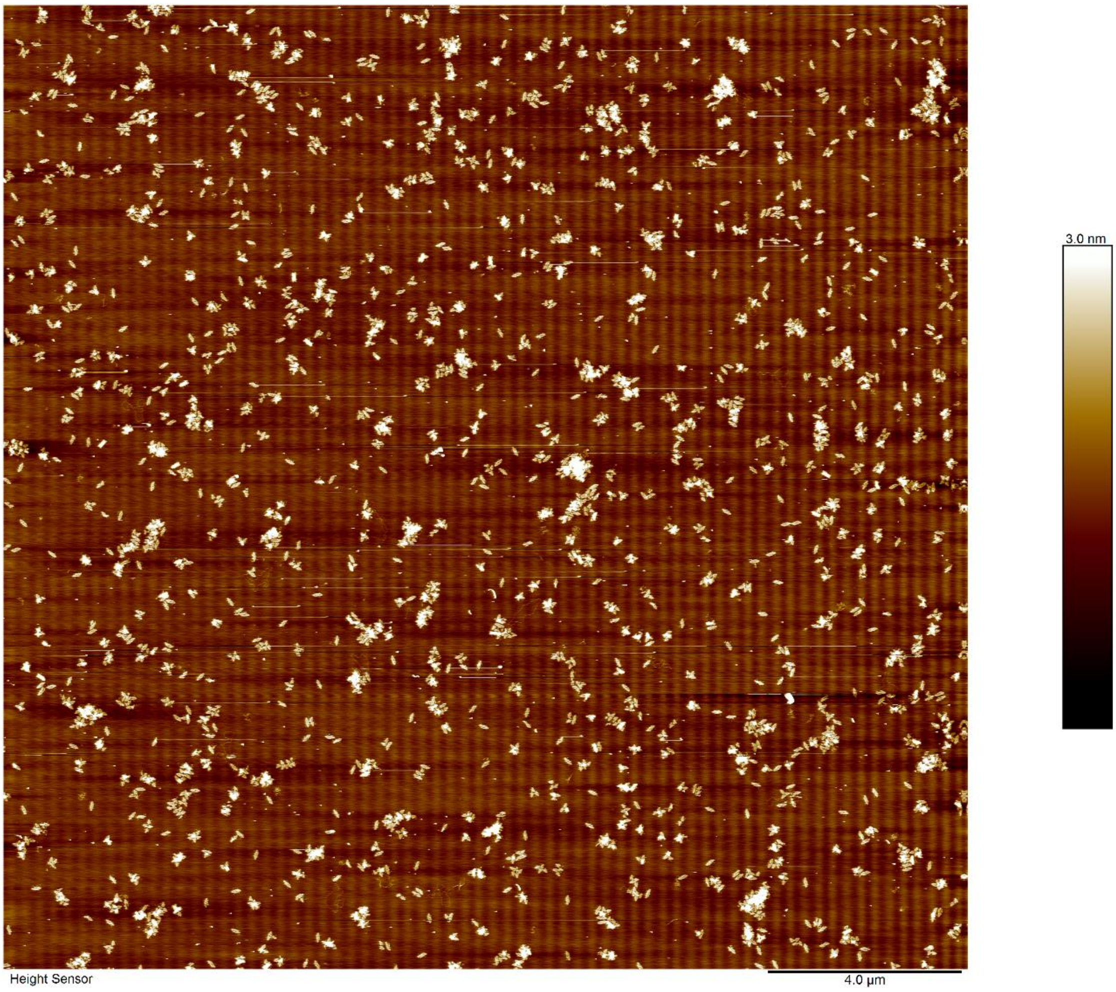

Figure S15. Truncated rectangle origami with 14 capture strands, capturing $(R_{F8})_2$-ODNs

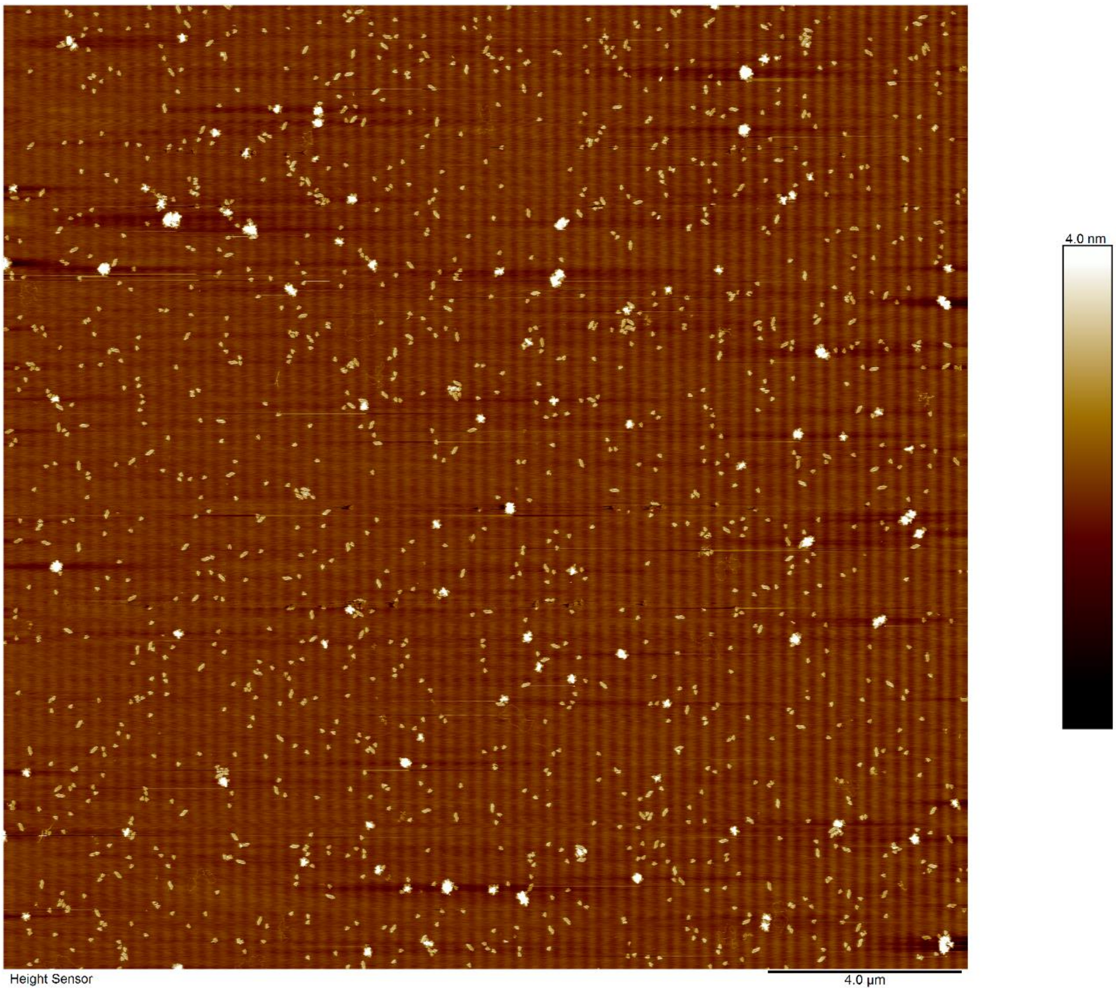

Figure S16. Truncated rectangle origami with 14 capture strands, capturing $(R_{F8})_4$-ODNs

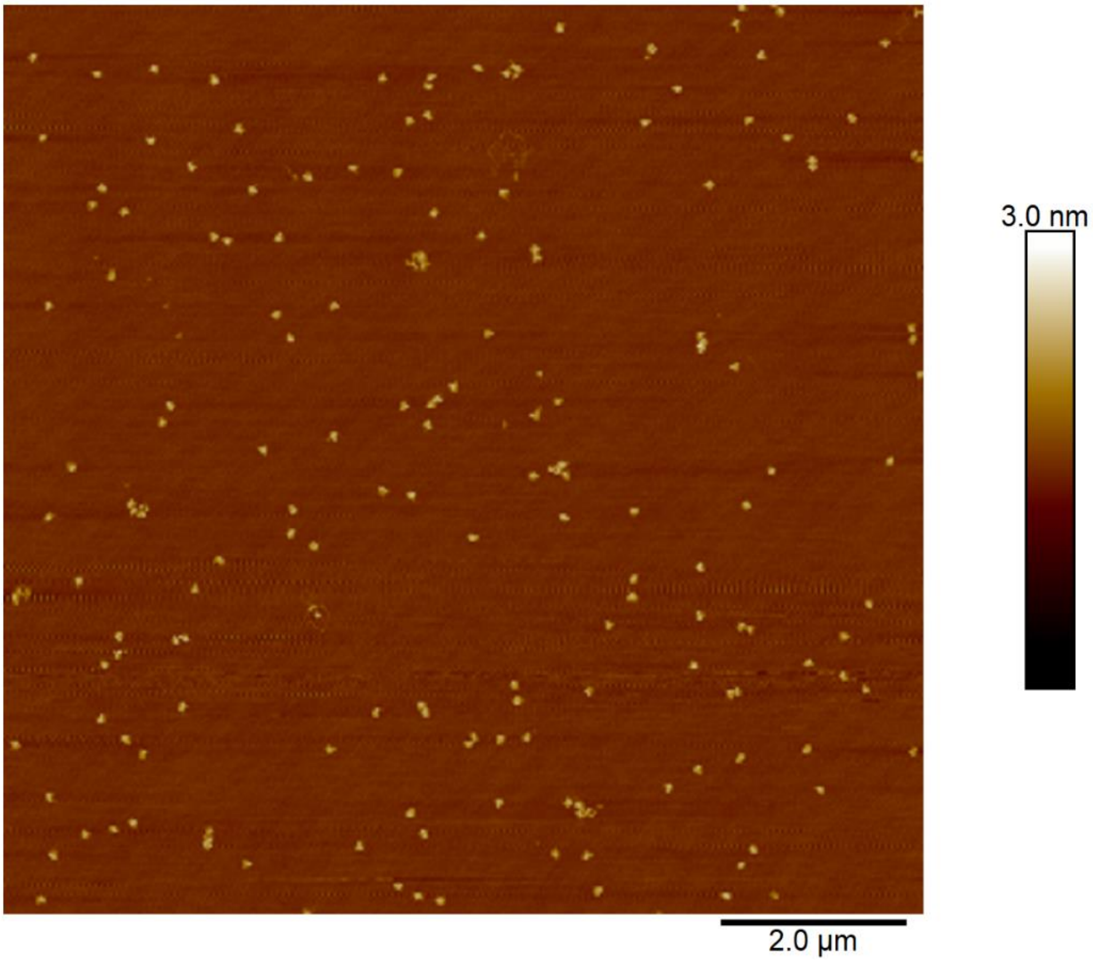

Figure S17. Truncated rectangle origami with 14 integrated alkyl-ODN tags

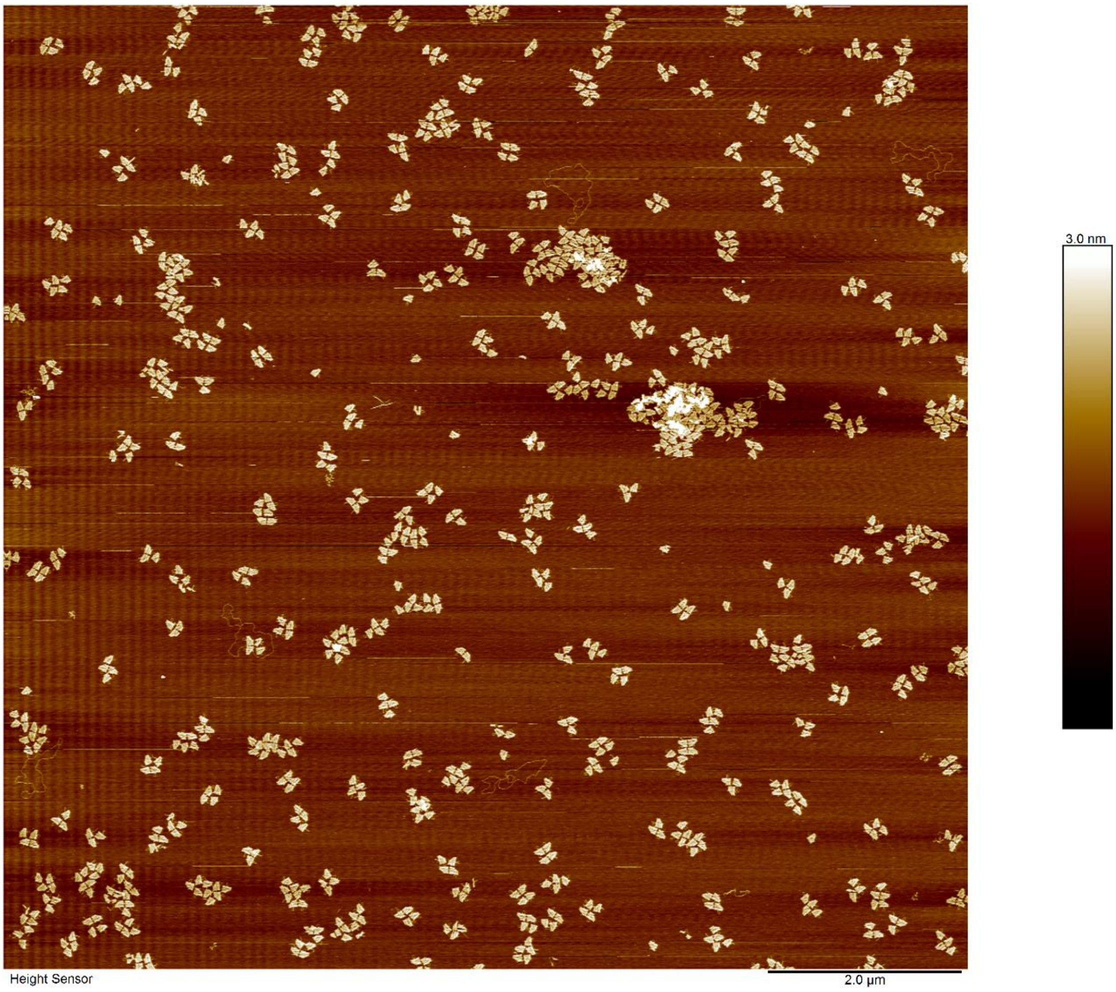

Figure S18. Truncated rectangle origami with 2 capture strands, capturing $(R_{F8})_2$-ODNs

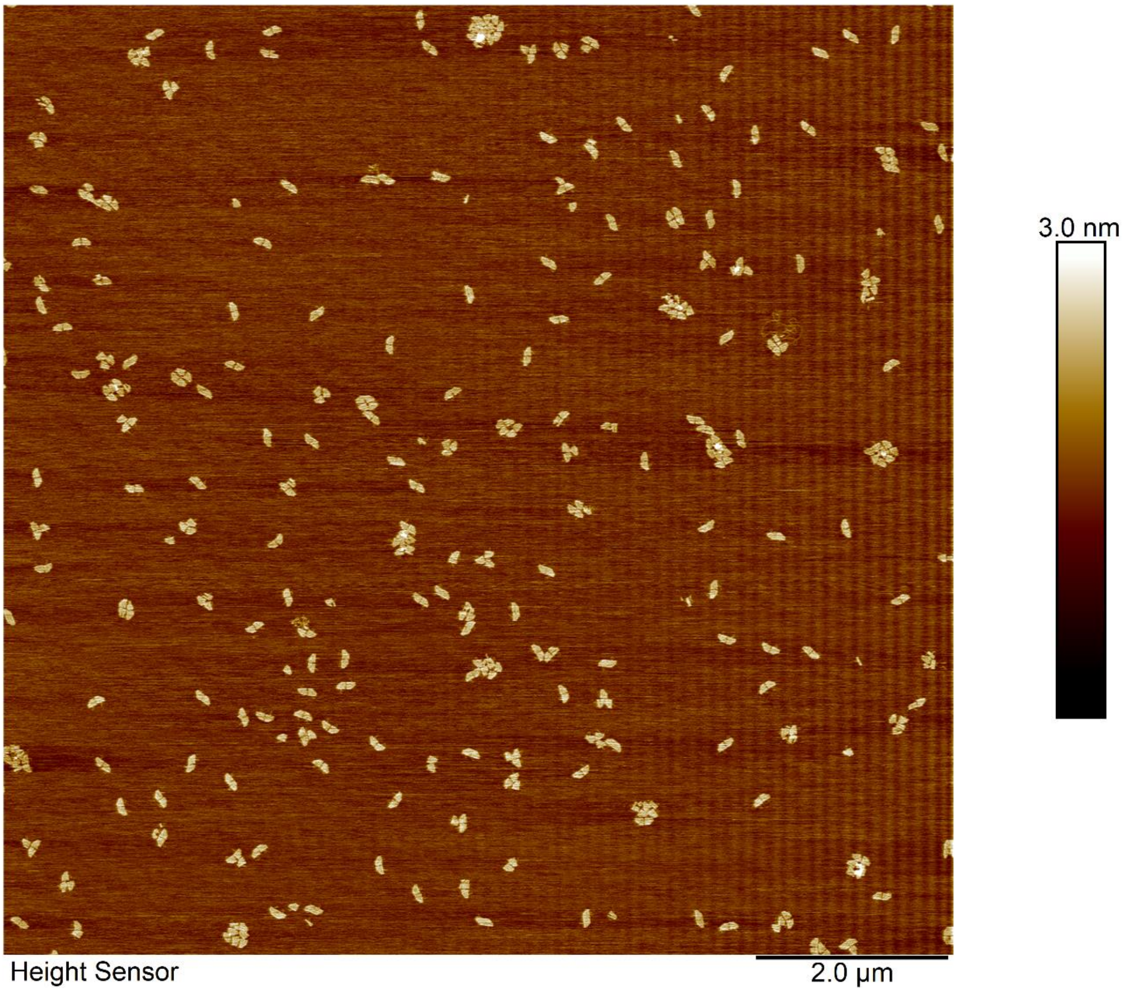

Figure S19. Truncated rectangle origami with 4 capture strands, capturing $(R_{F8})_2$-ODNs

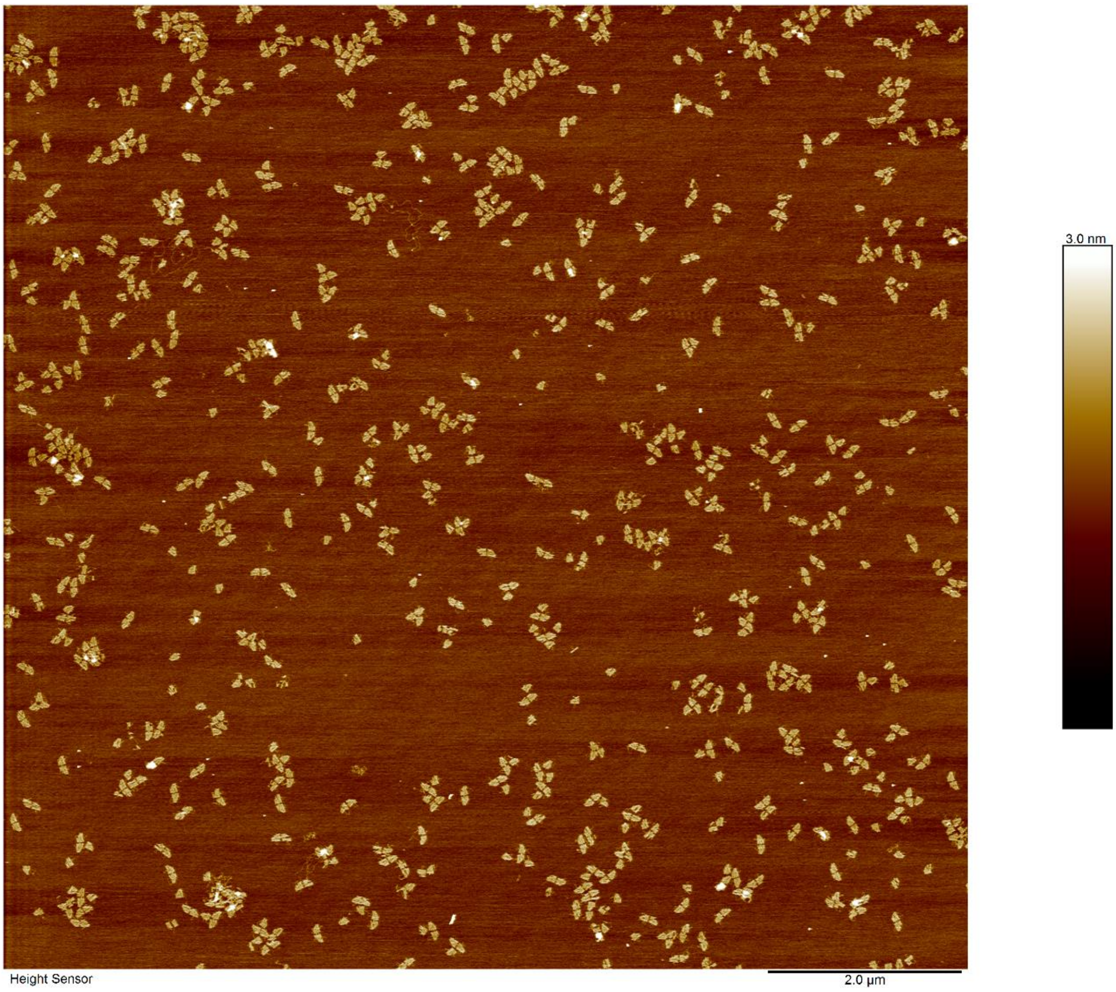

Figure S20. Truncated rectangle origami with 6 capture strands, capturing $(R_{F8})_2$-ODNs

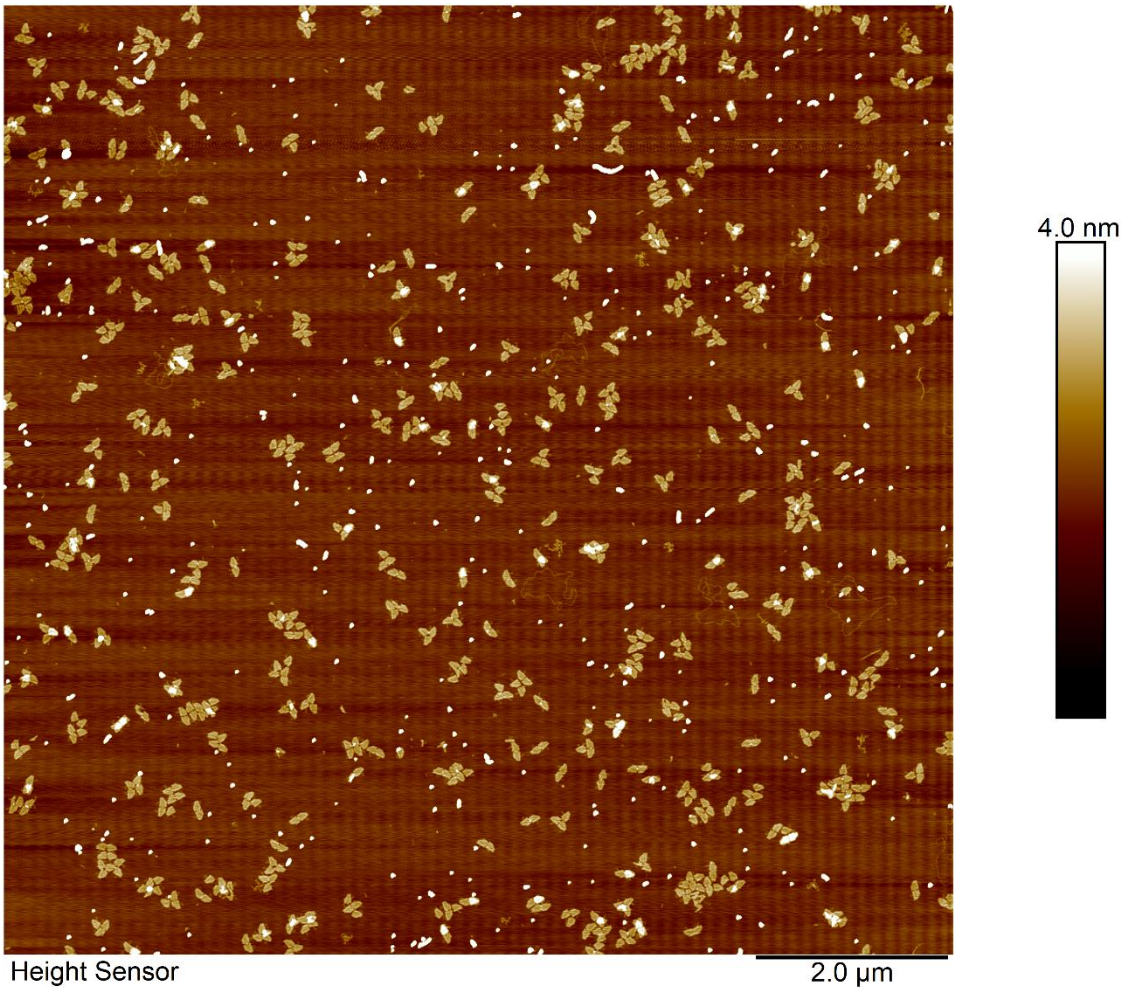

Figure S21. Truncated rectangle origami with 8 capture strands, capturing $(R_{F8})_2$-ODNs

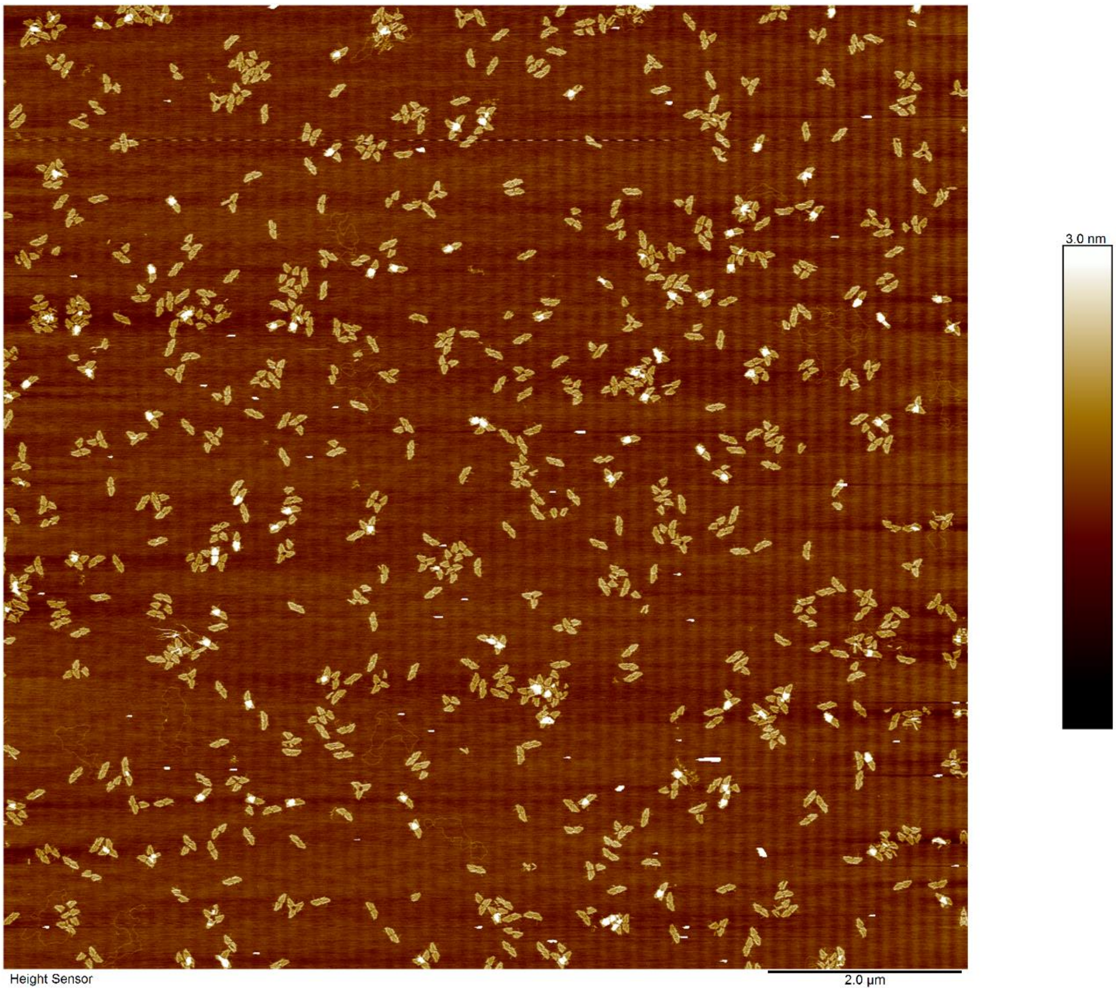

Figure S22. Truncated rectangle origami with 10 capture strands, capturing $(R_{F8})_2$-ODNs

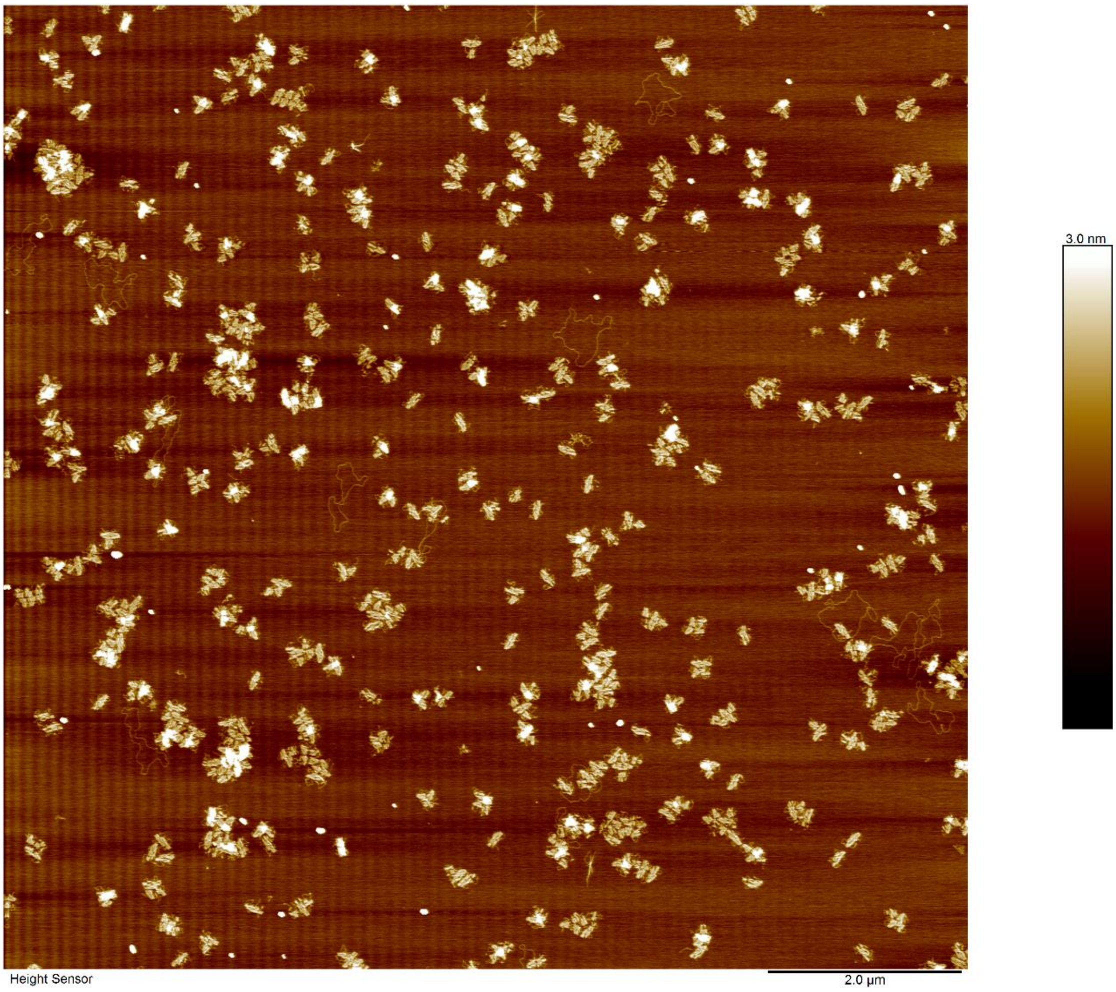

Figure S23. Truncated rectangle origami with 12 capture strands, capturing $(R_{F8})_2$-ODNs

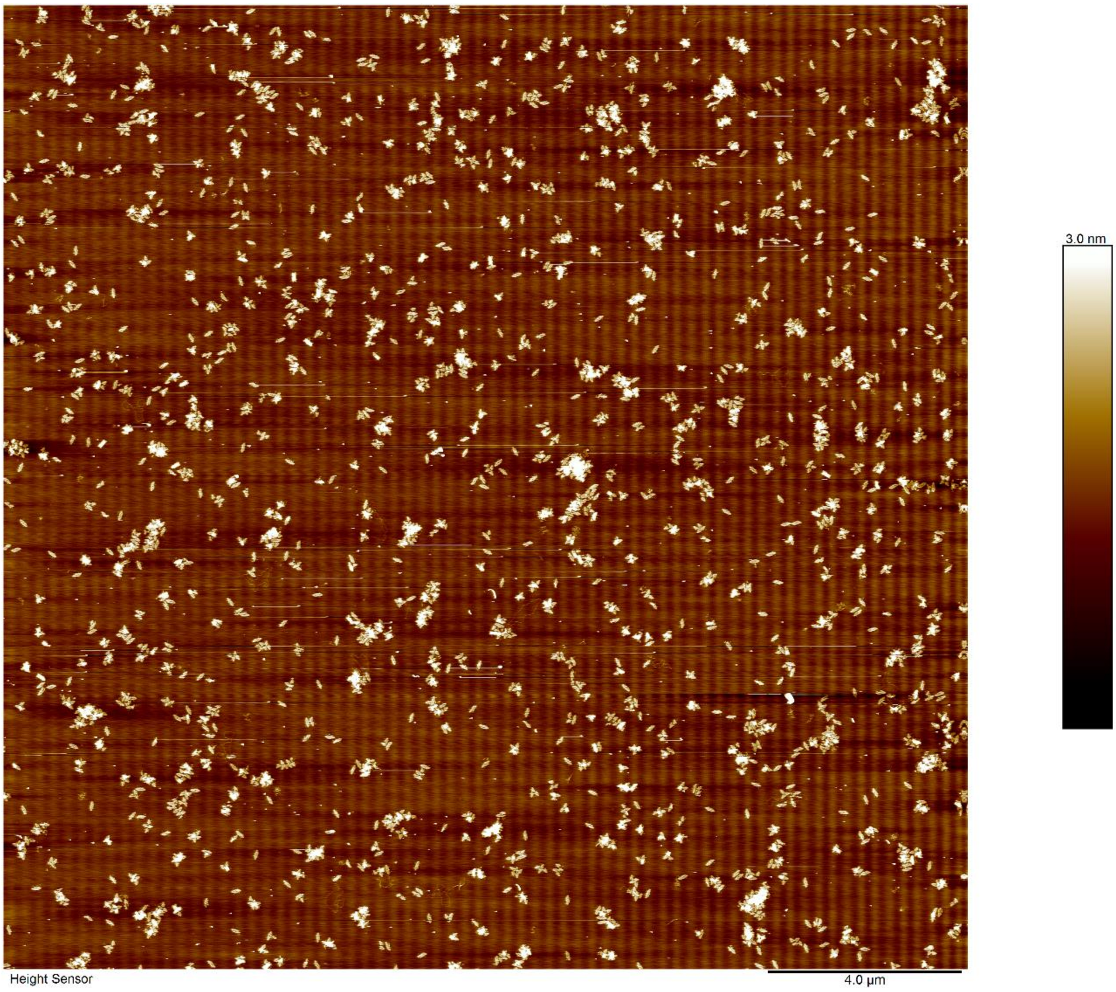

Figure S24. Truncated rectangle origami with 14 capture strands, capturing $(R_{F8})_2$-ODNs

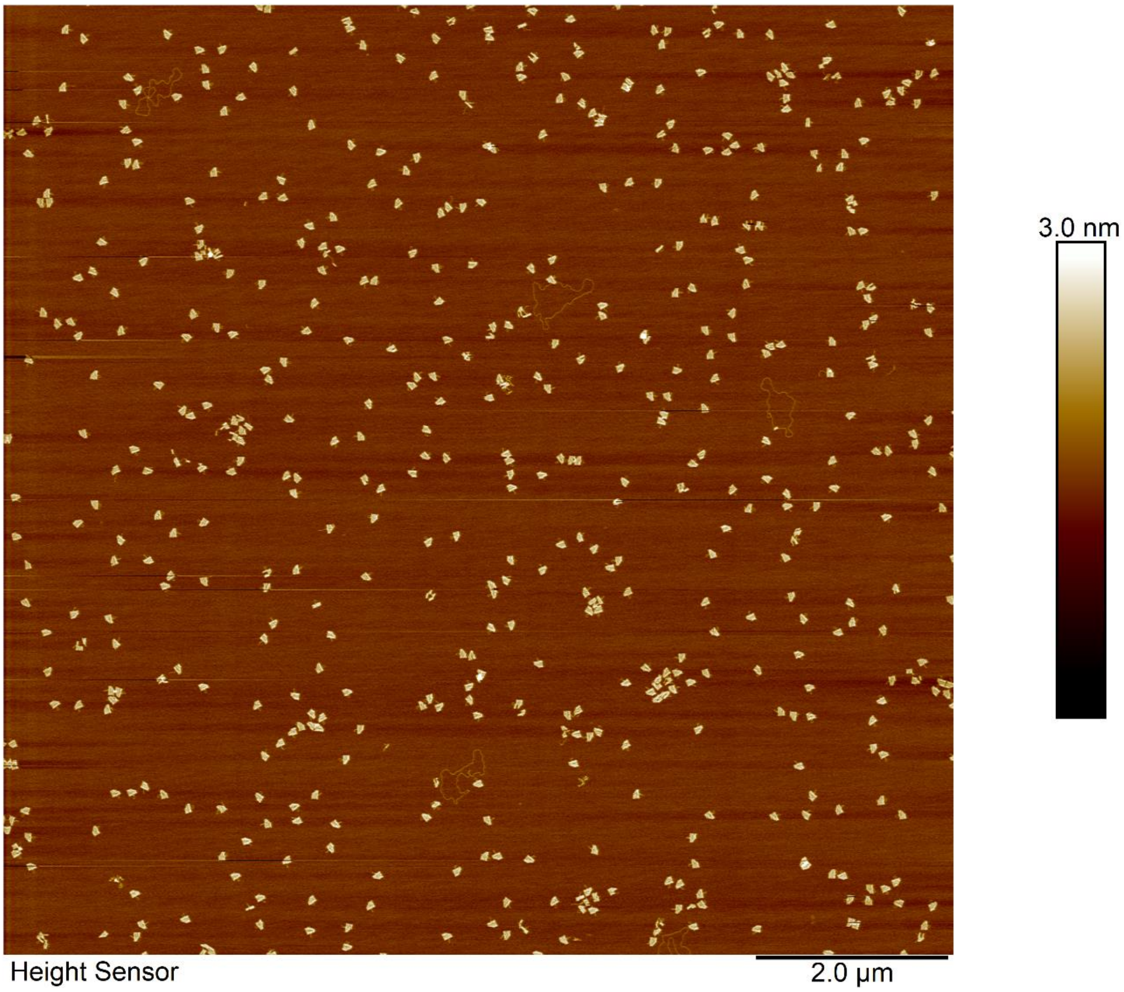

Figure S25. Truncated rectangle origami with integrated strands, 2× $(R_{F8})_2$-ODNs

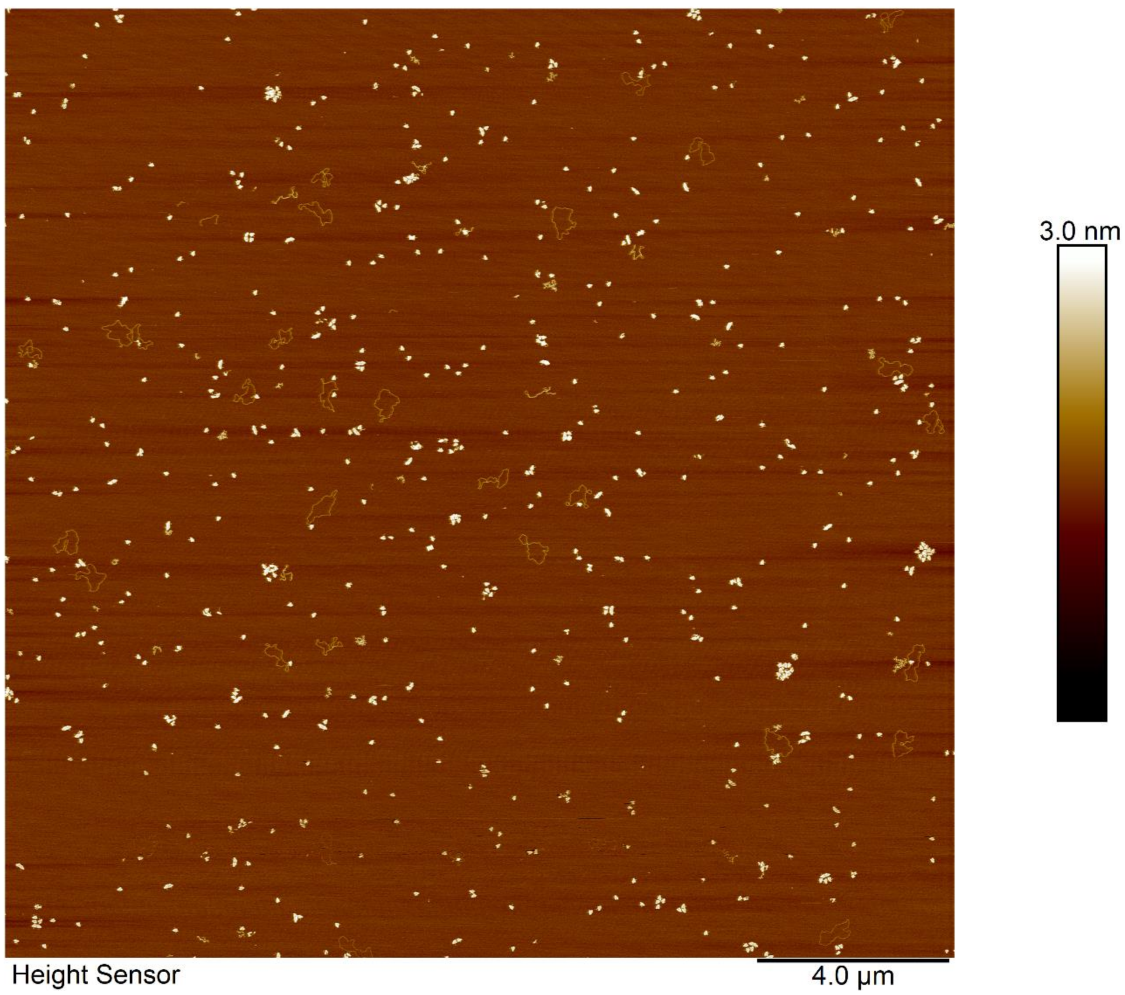

Figure S26. Truncated rectangle origami with integrated strands, 4× $(R_{F8})_2$-ODNs

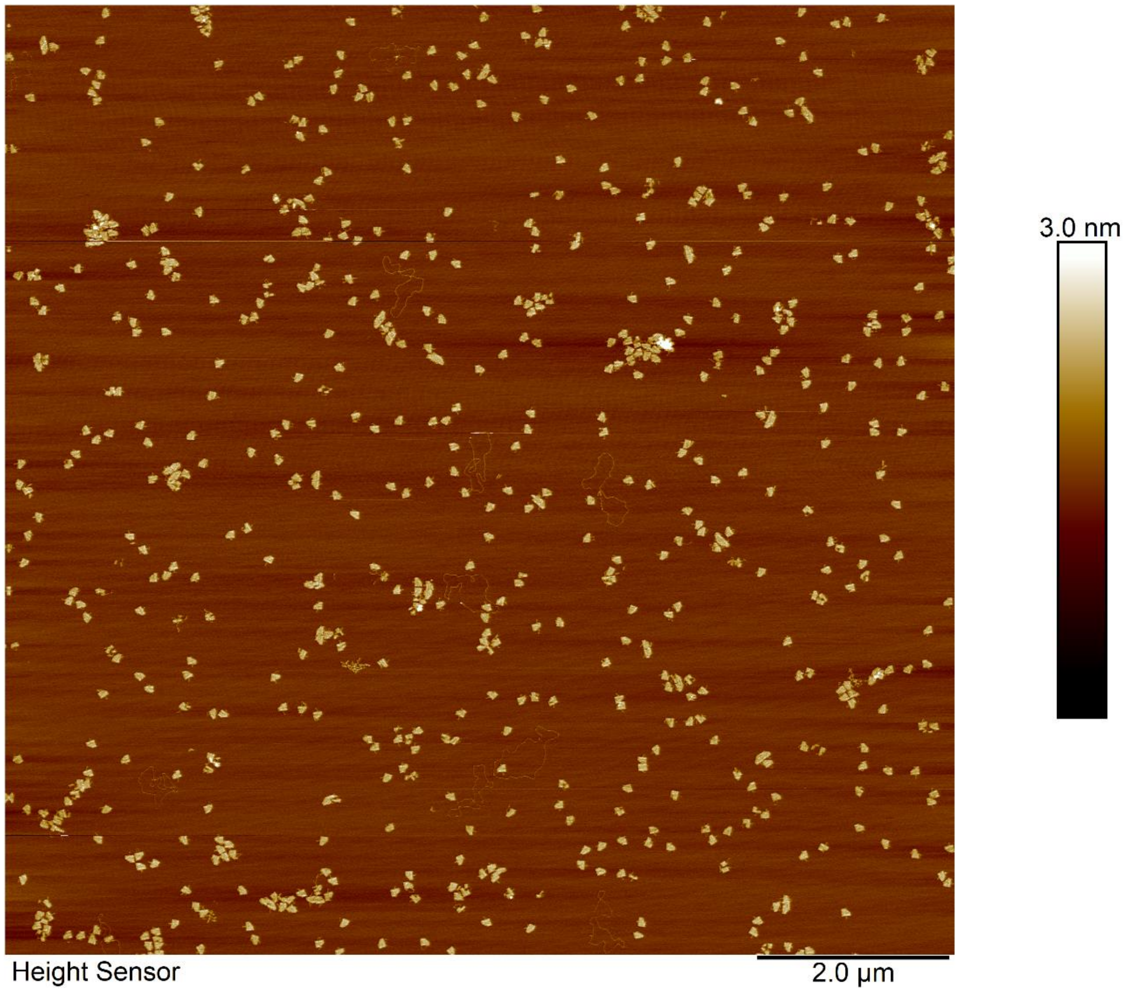

Figure S27. Truncated rectangle origami with integrated strands, 6× $(R_{F8})_2$-ODNs

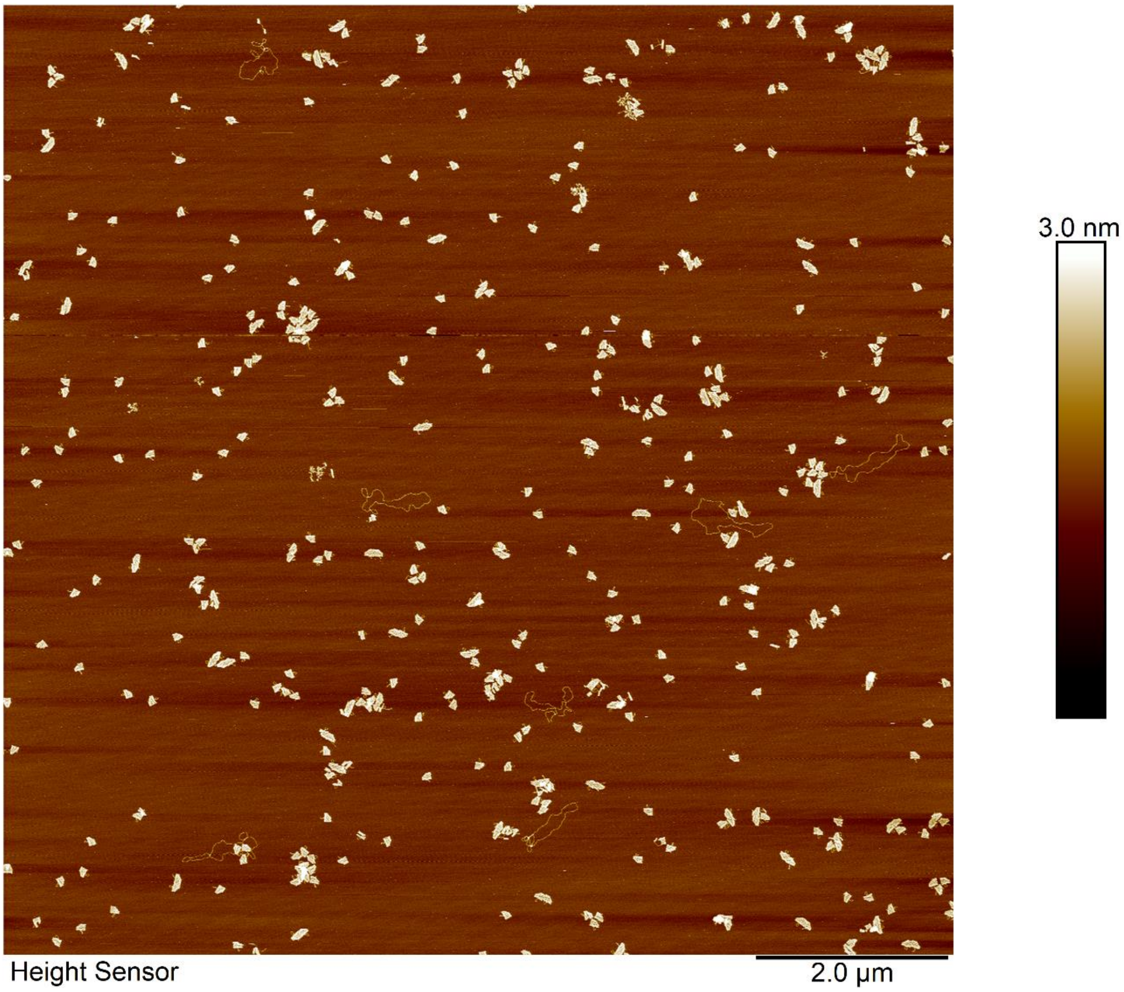

Figure S28. Truncated rectangle origami with integrated strands, 8× $(R_{F8})_2$-ODNs

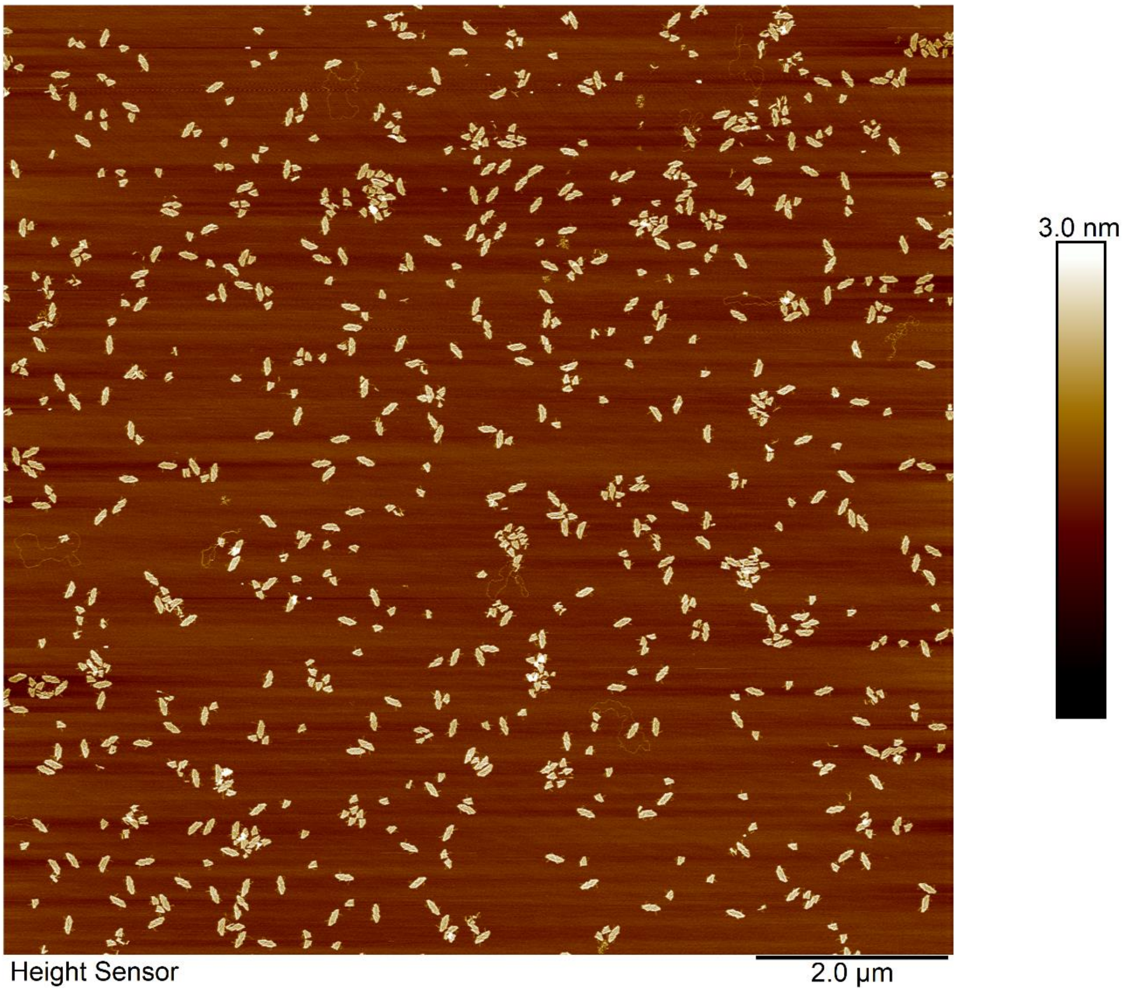

Figure S29. Truncated rectangle origami with integrated strands, 10× $(R_{F8})_2$-ODNs

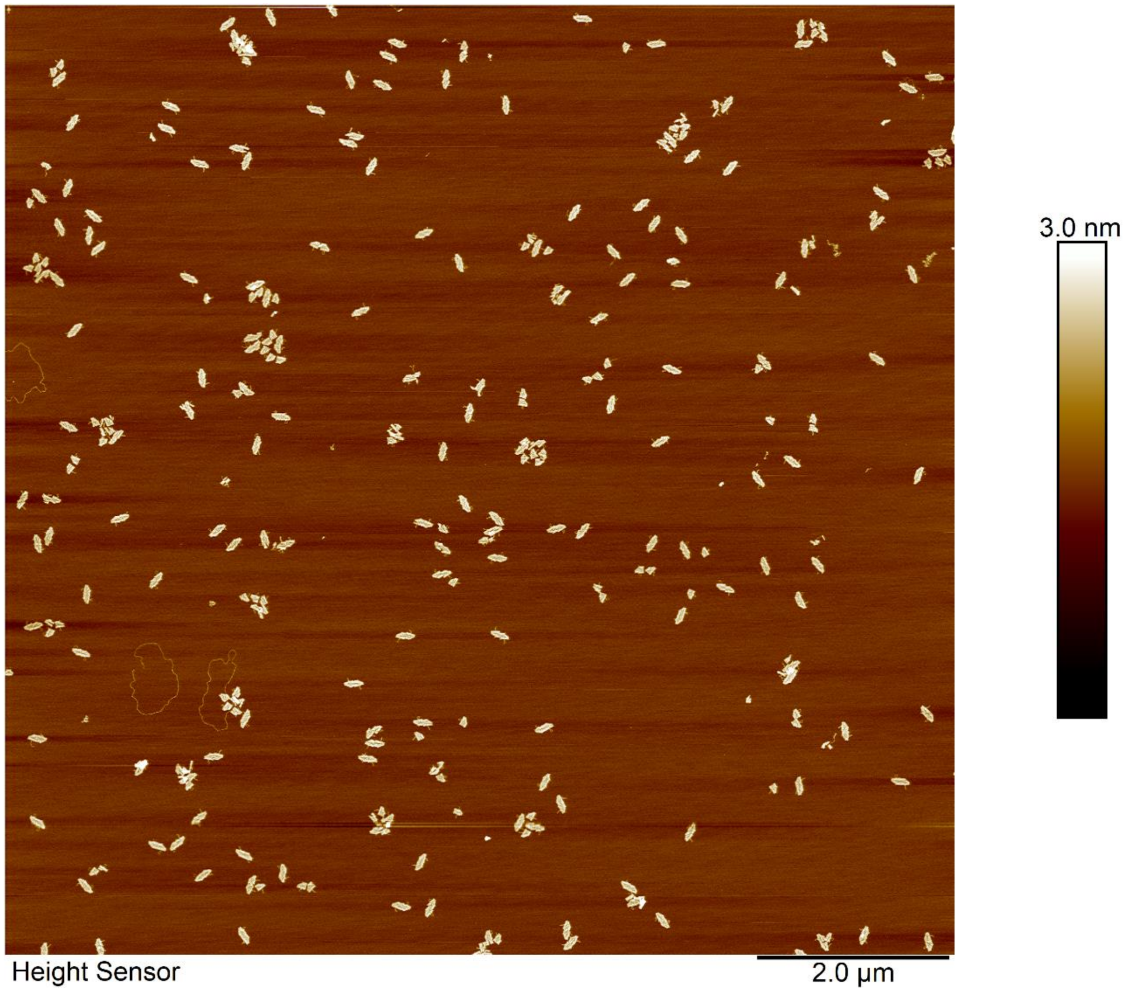

Figure S30. Truncated rectangle origami with integrated strands, 12× $(R_{F8})_2$-ODNs

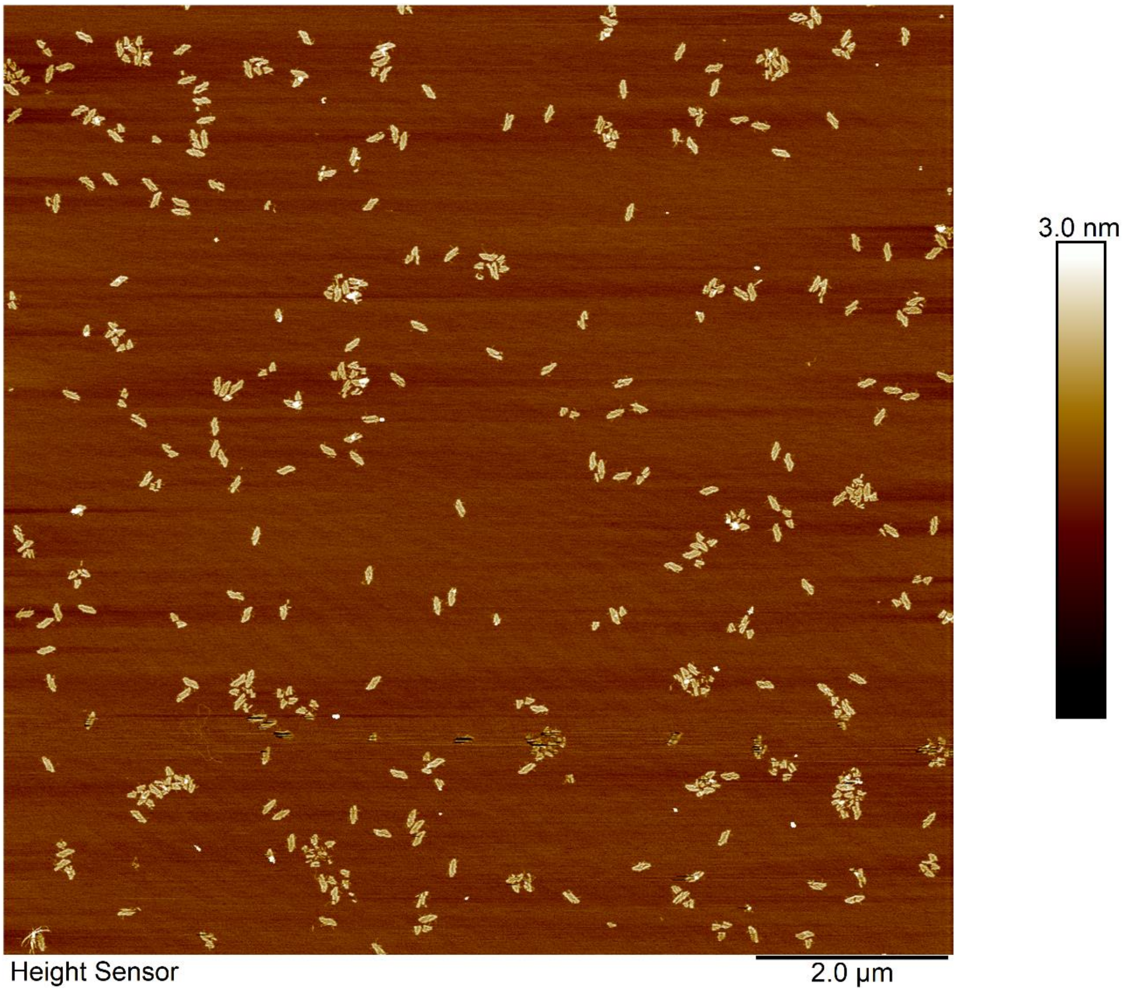

Figure S31. Truncated rectangle origami with integrated strands, 14× $(R_{F8})_2$-ODNs

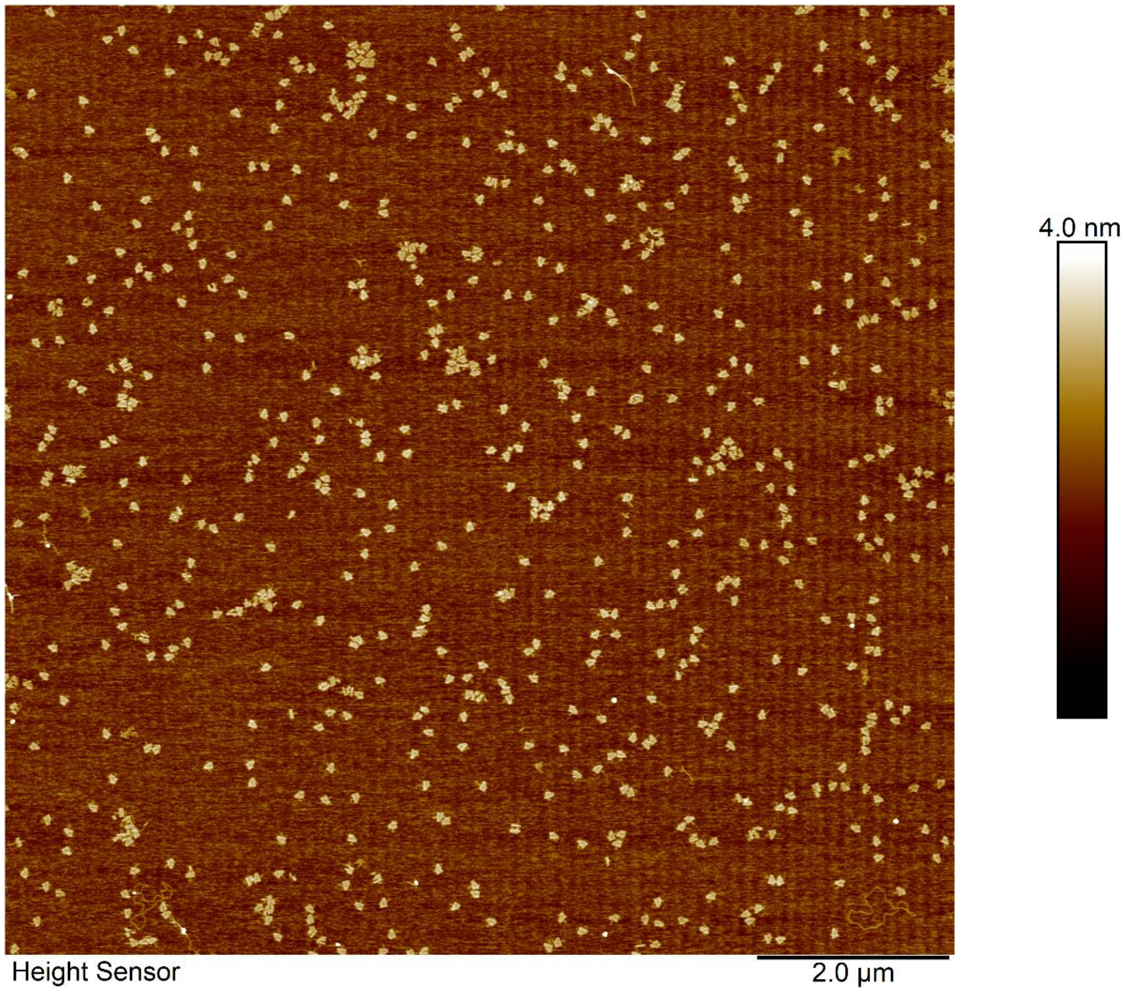

Figure S32. Truncated rectangle origami with integrated strands, 2× 5nt sticky ends.

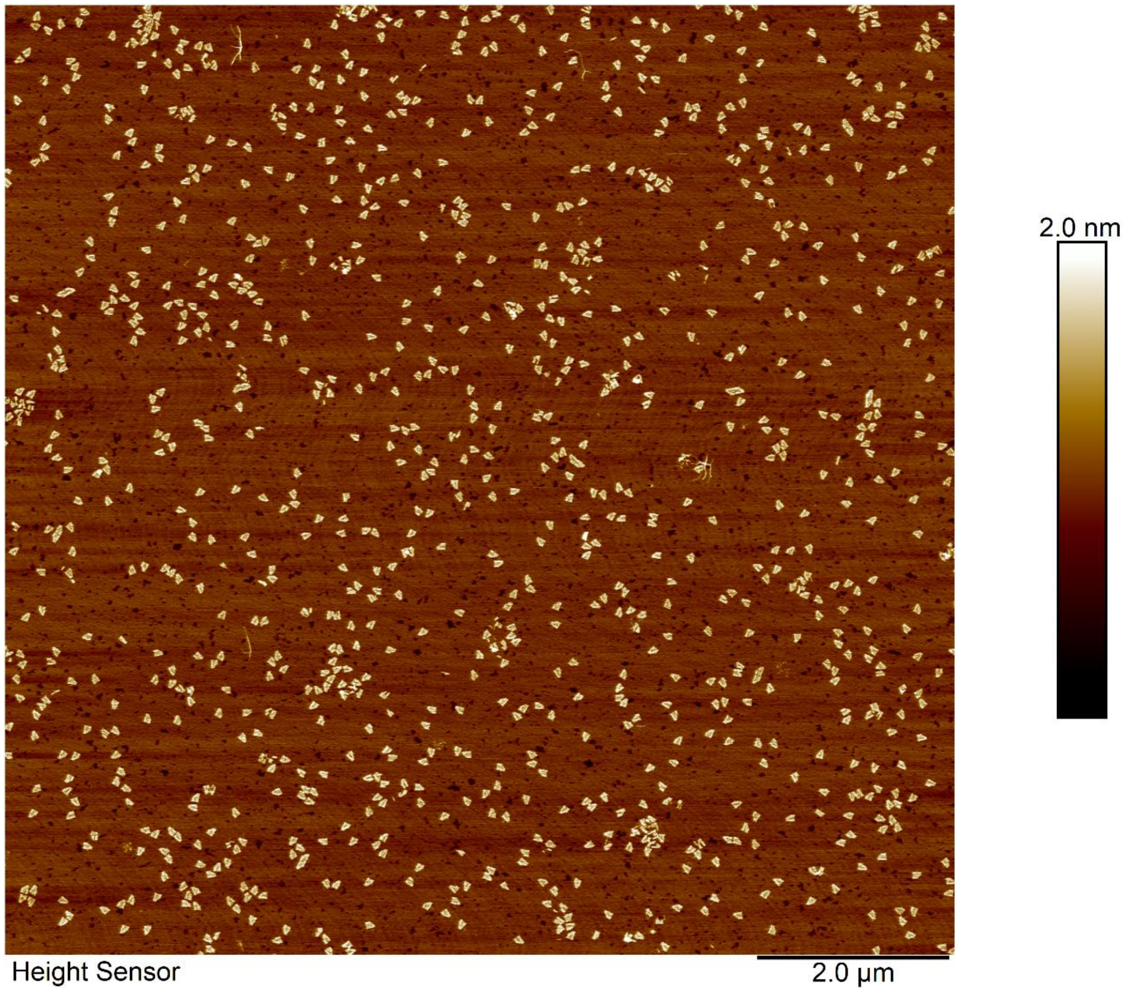

Figure S33. Truncated rectangle origami with integrated strands, 4× 5nt sticky ends.

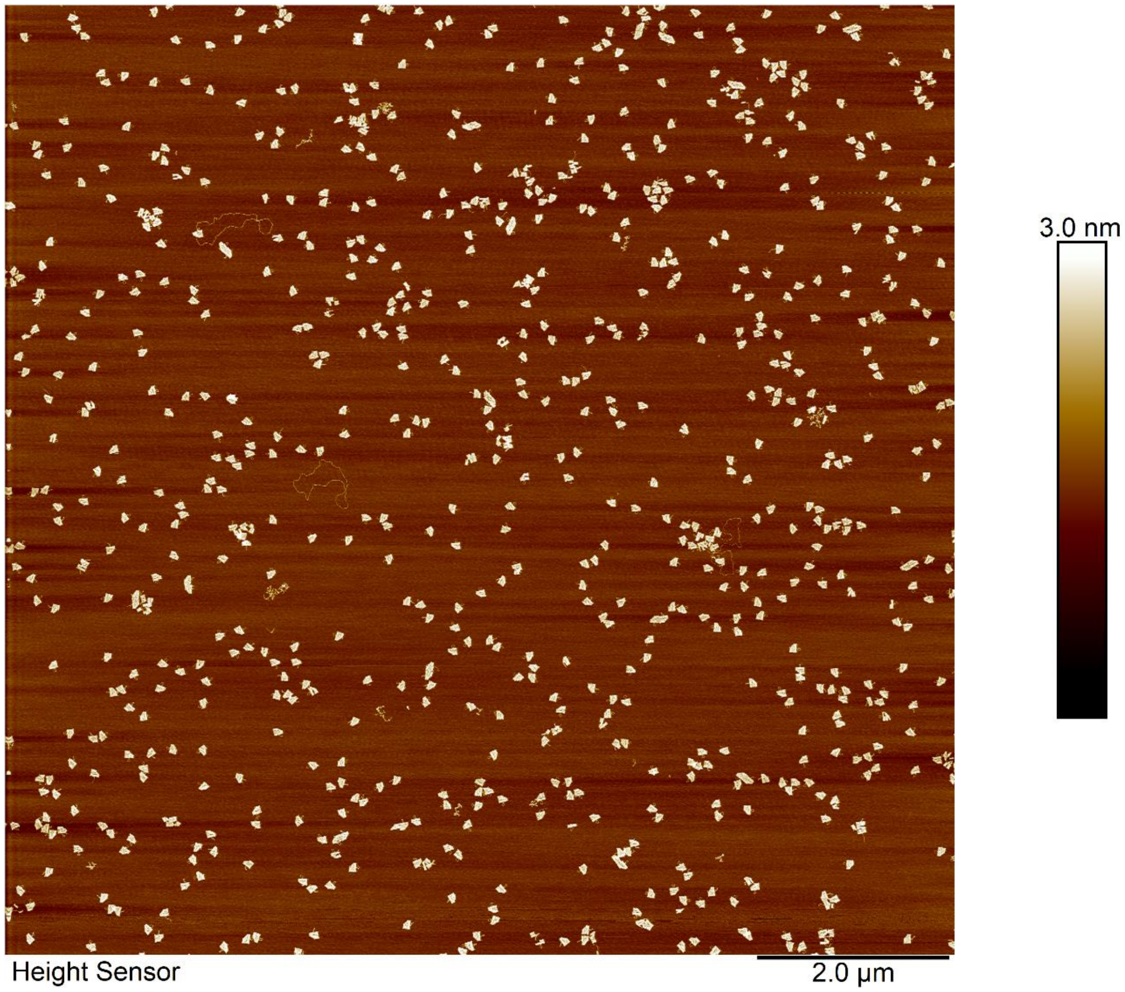

Figure S34. Truncated rectangle origami with integrated strands, 6× 5nt sticky ends.

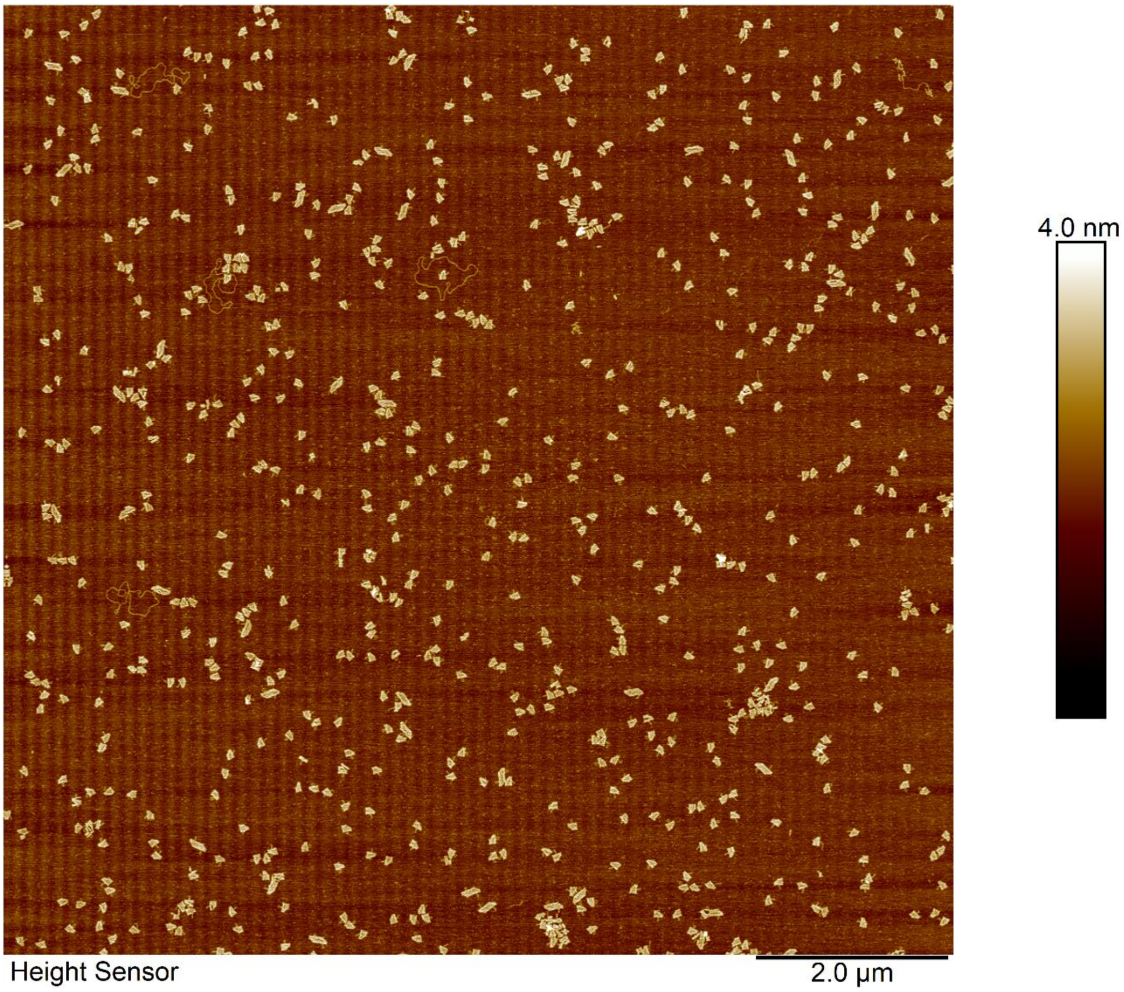

Figure S35. Truncated rectangle origami with integrated strands, 8× 5nt sticky ends.

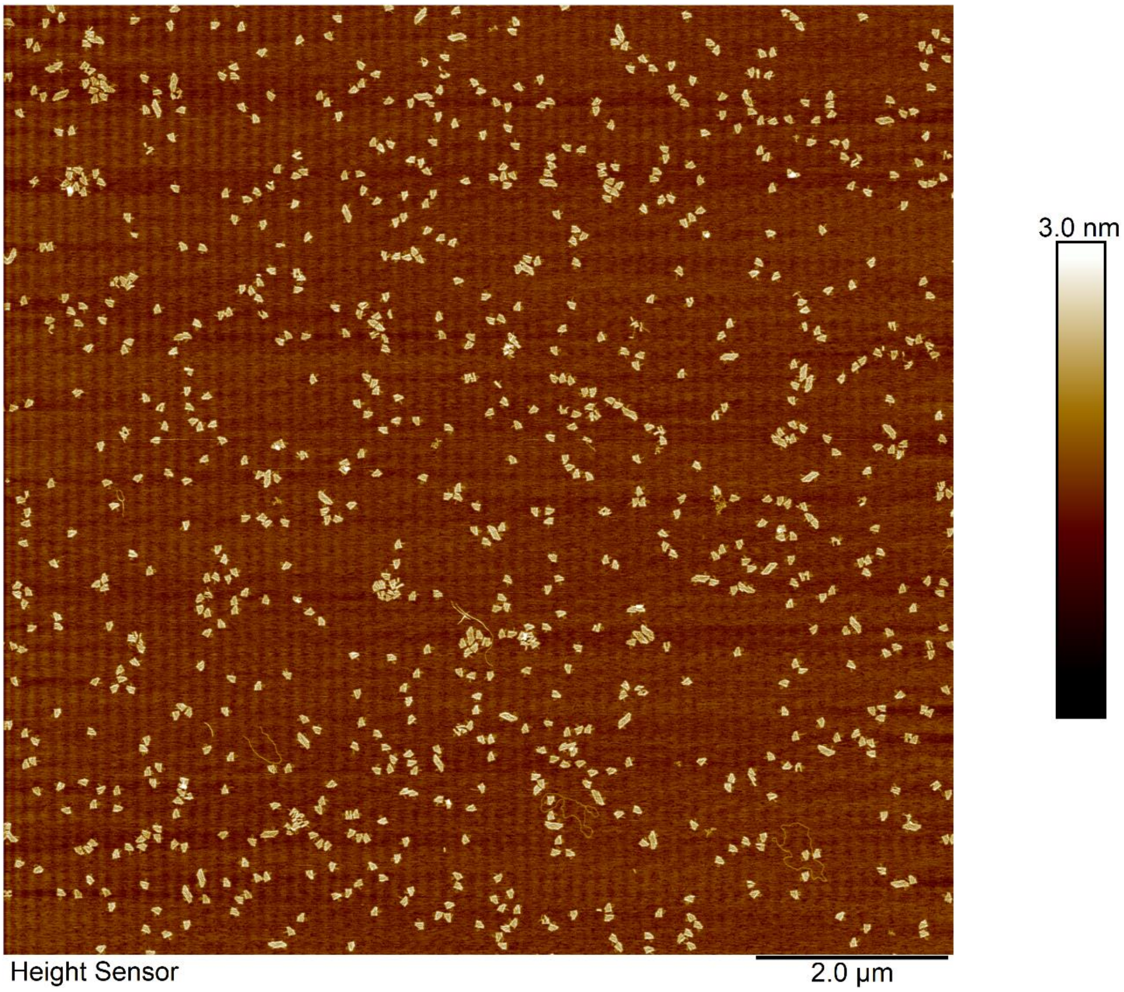

Figure S36. Truncated rectangle origami with integrated strands, 10× 5nt sticky ends.

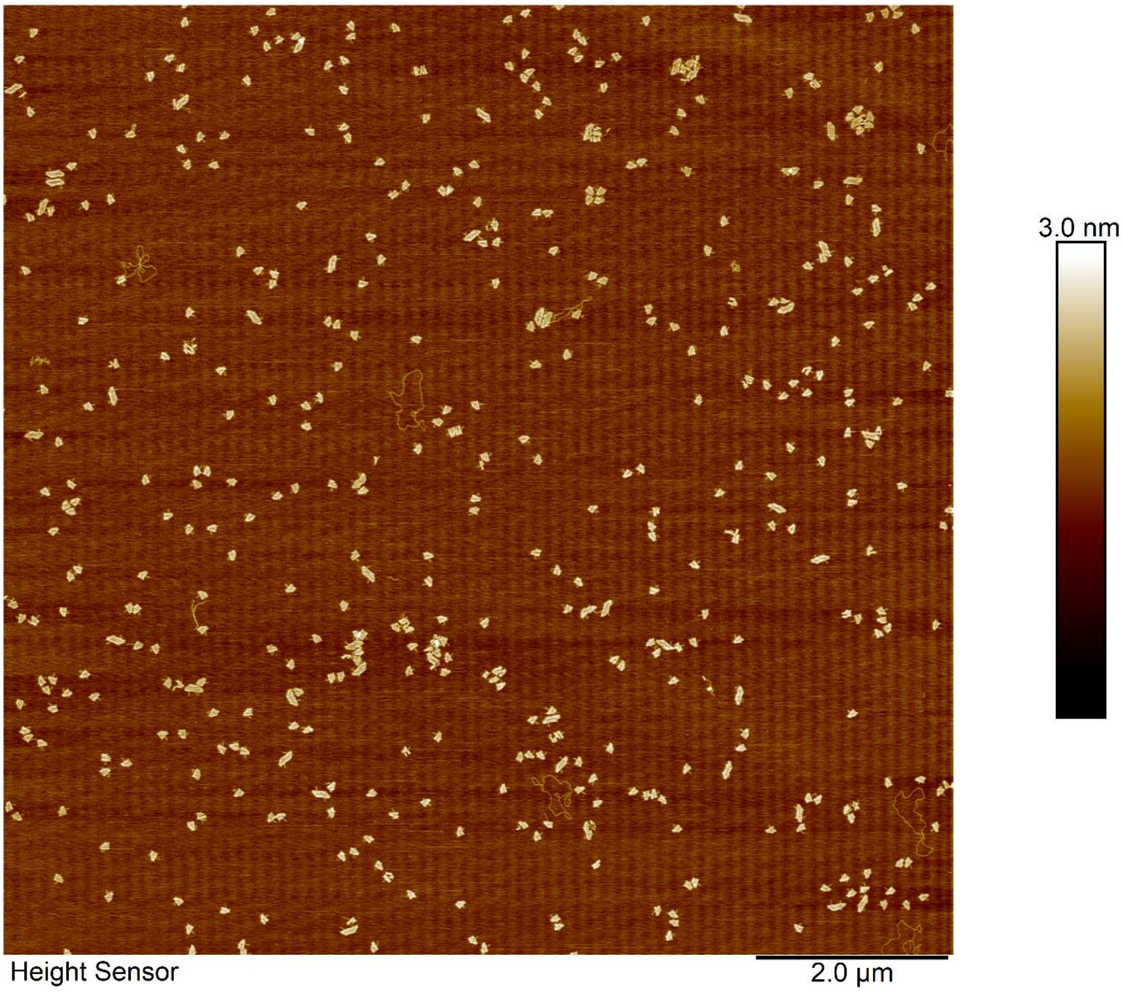

Figure S37. Truncated rectangle origami with integrated strands, 12× 5nt sticky ends.

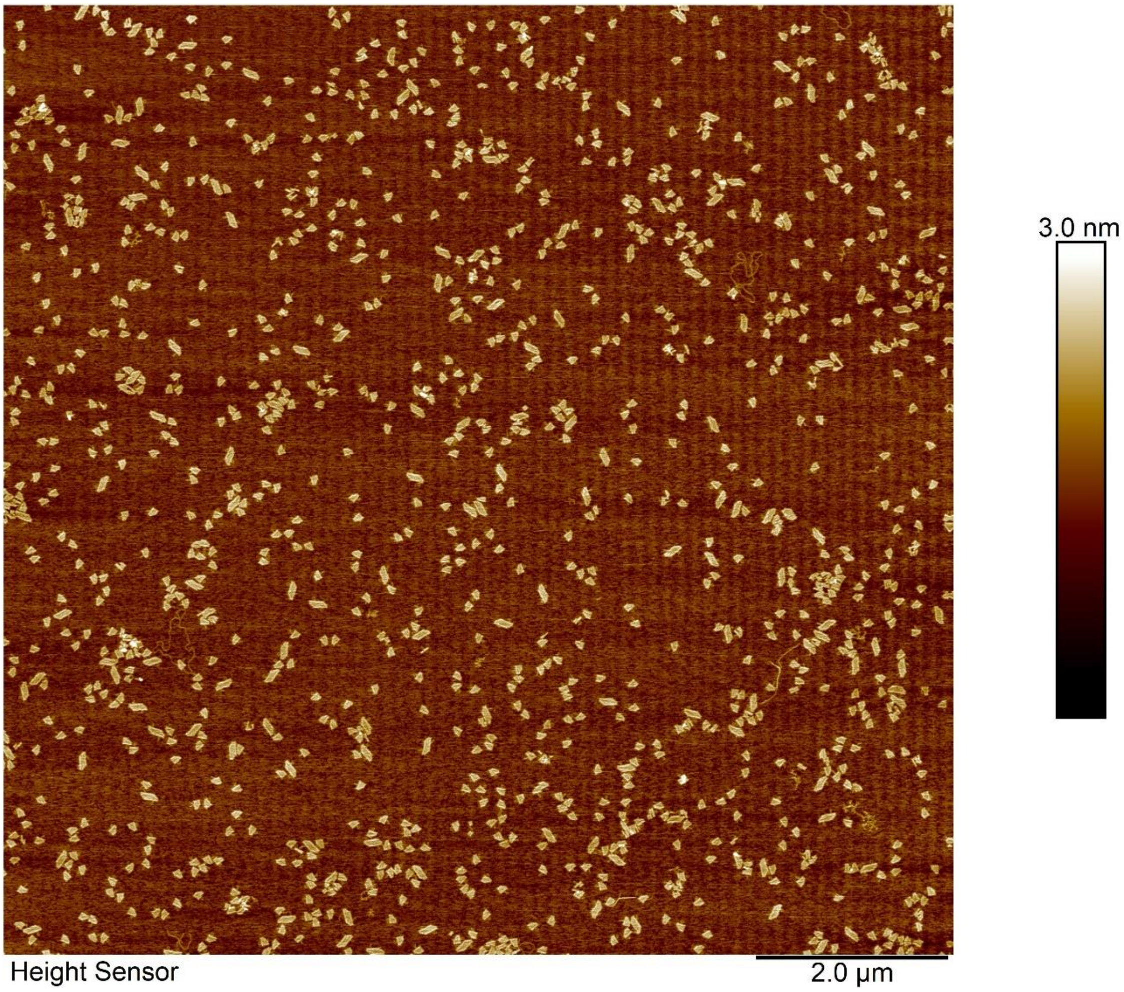

Figure S38. Truncated rectangle origami with integrated strands, 14× 5nt sticky ends.

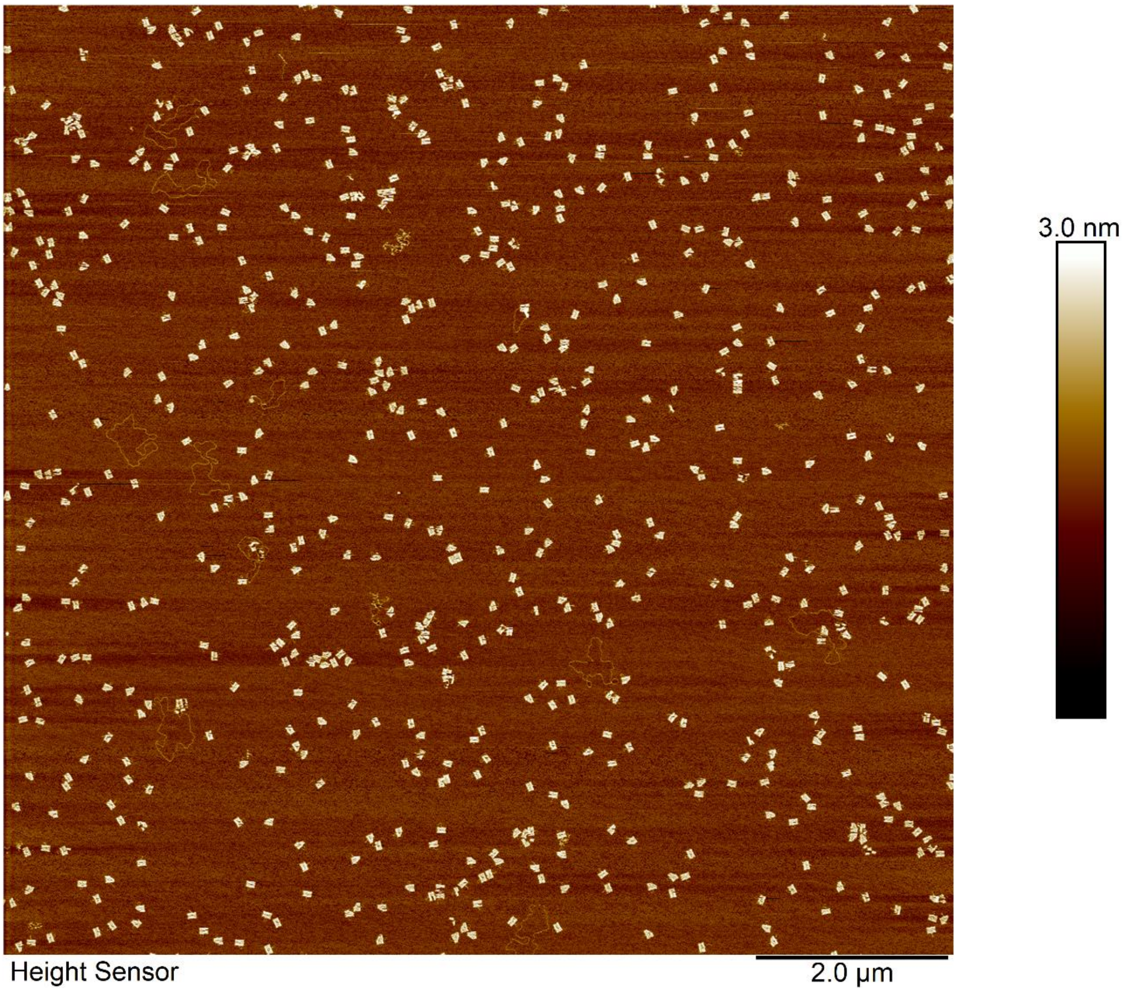

Figure S39. Truncated rectangle and rectangle origami tiles with integrated strands, each with 2× $(R_{F8})_2$.

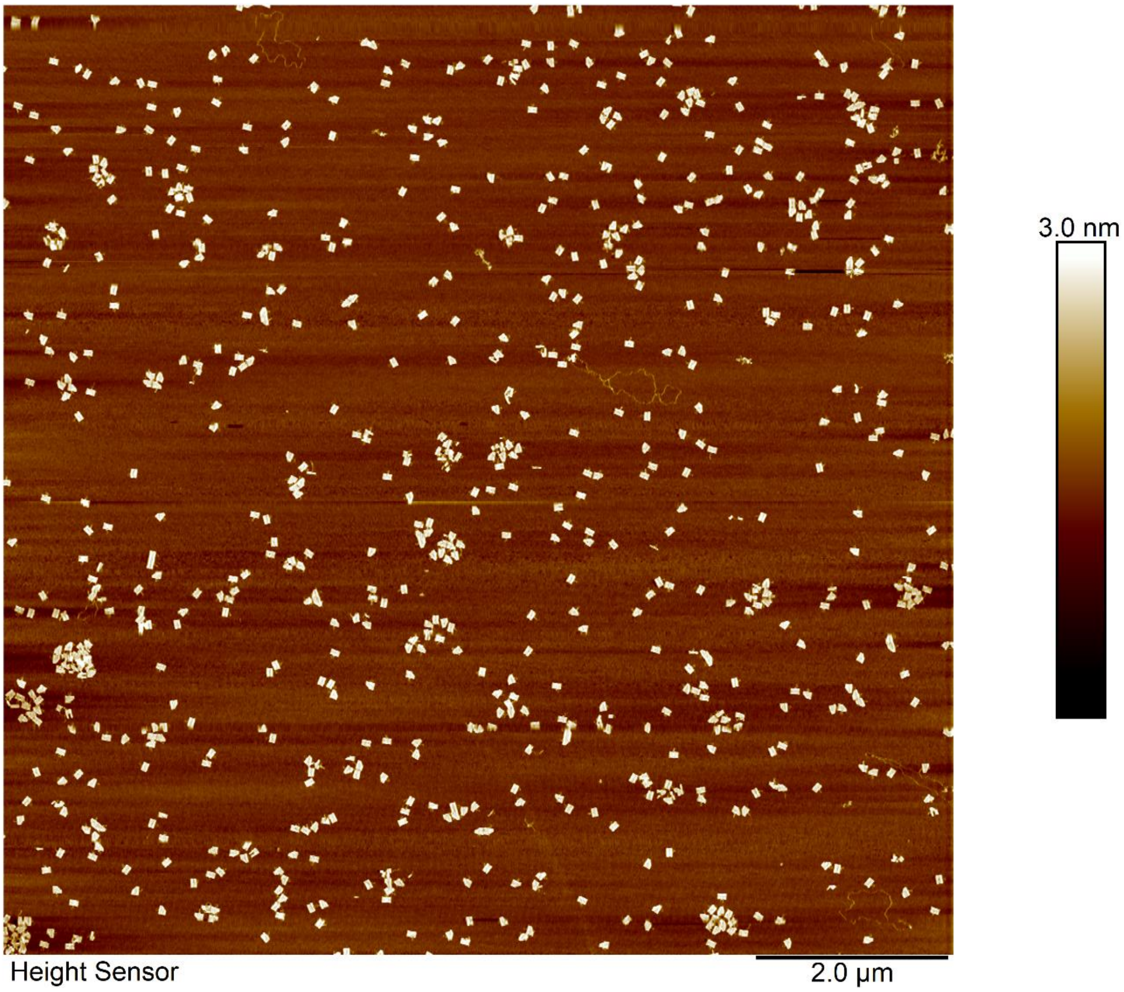

Figure S40. Truncated rectangle and rectangle origami tiles with integrated strands, each with 4× $(R_{F8})_2$.

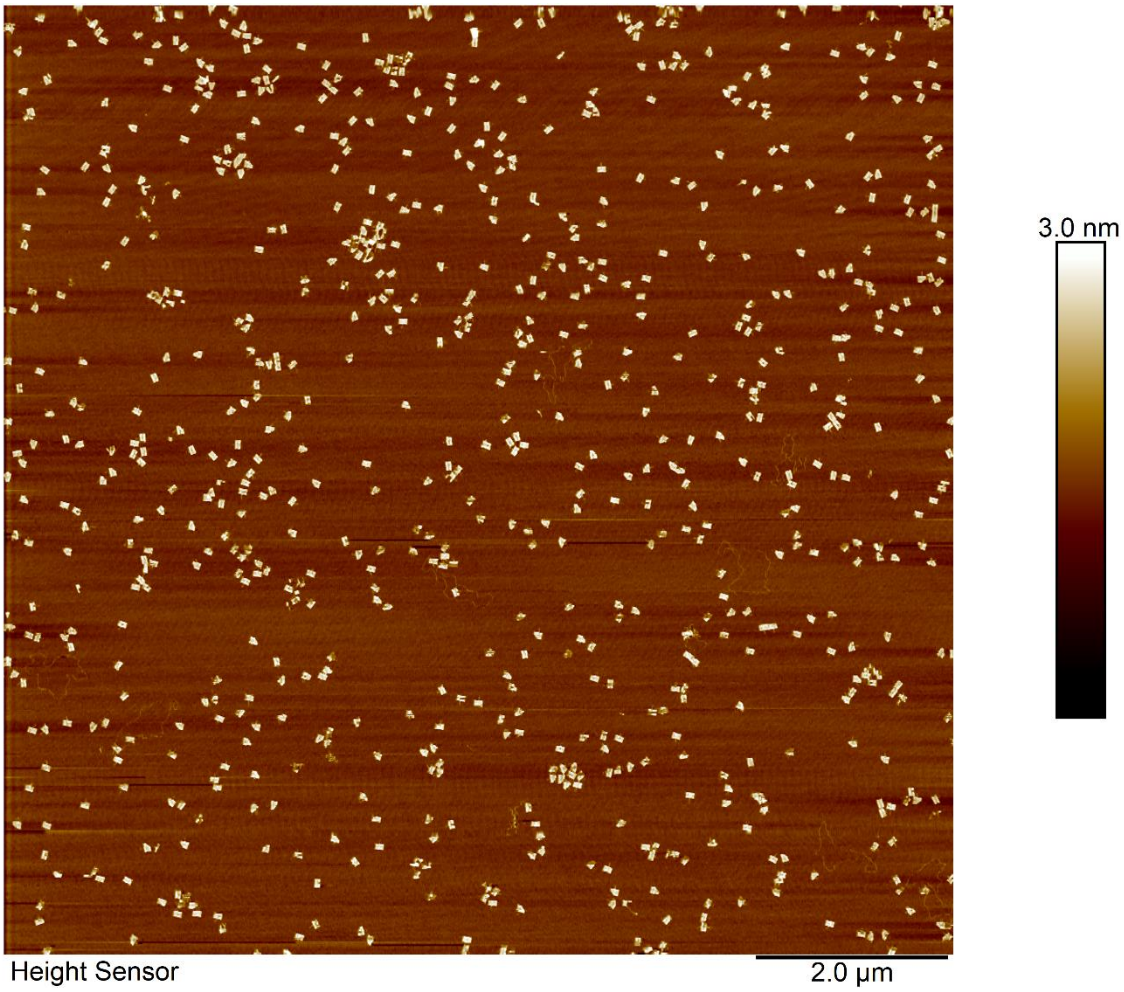

Figure S41. Truncated rectangle and rectangle origami tiles with integrated strands, each with 6× $(R_{F8})_2$.

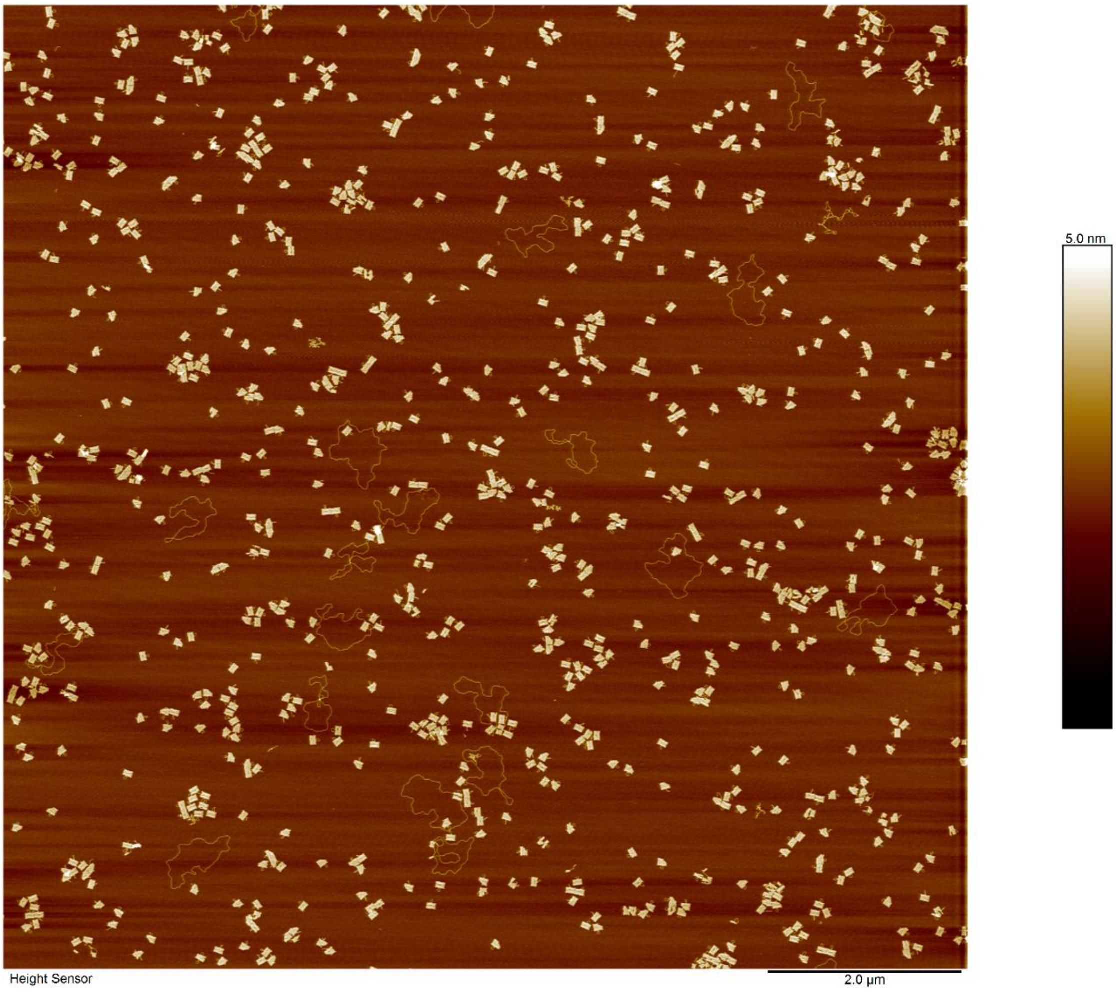

Figure S42. Truncated rectangle and rectangle origami tiles with integrated strands, each with 8× $(R_{F8})_2$.

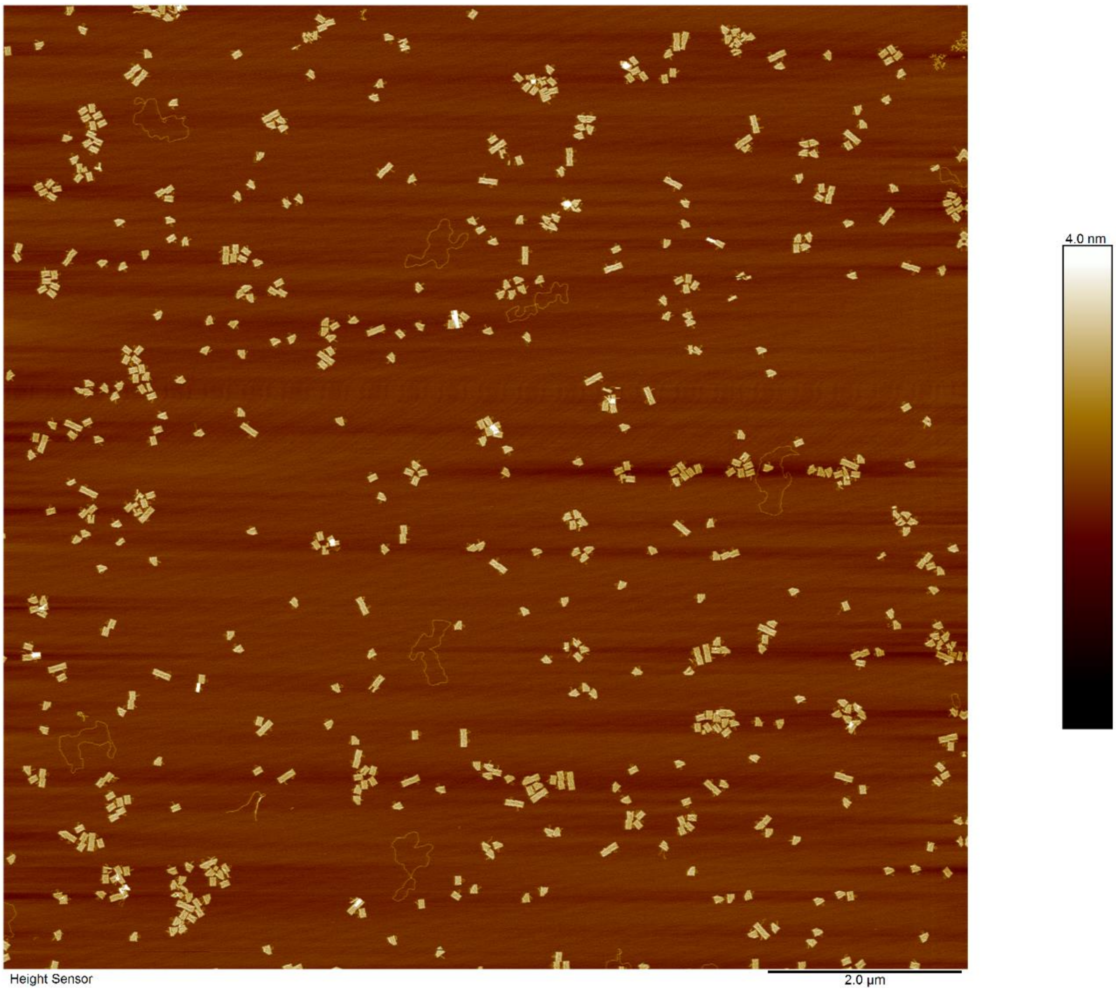

Figure S43. Truncated rectangle and rectangle origami tiles with integrated strands, each with 10× $(R_{F8})_2$.

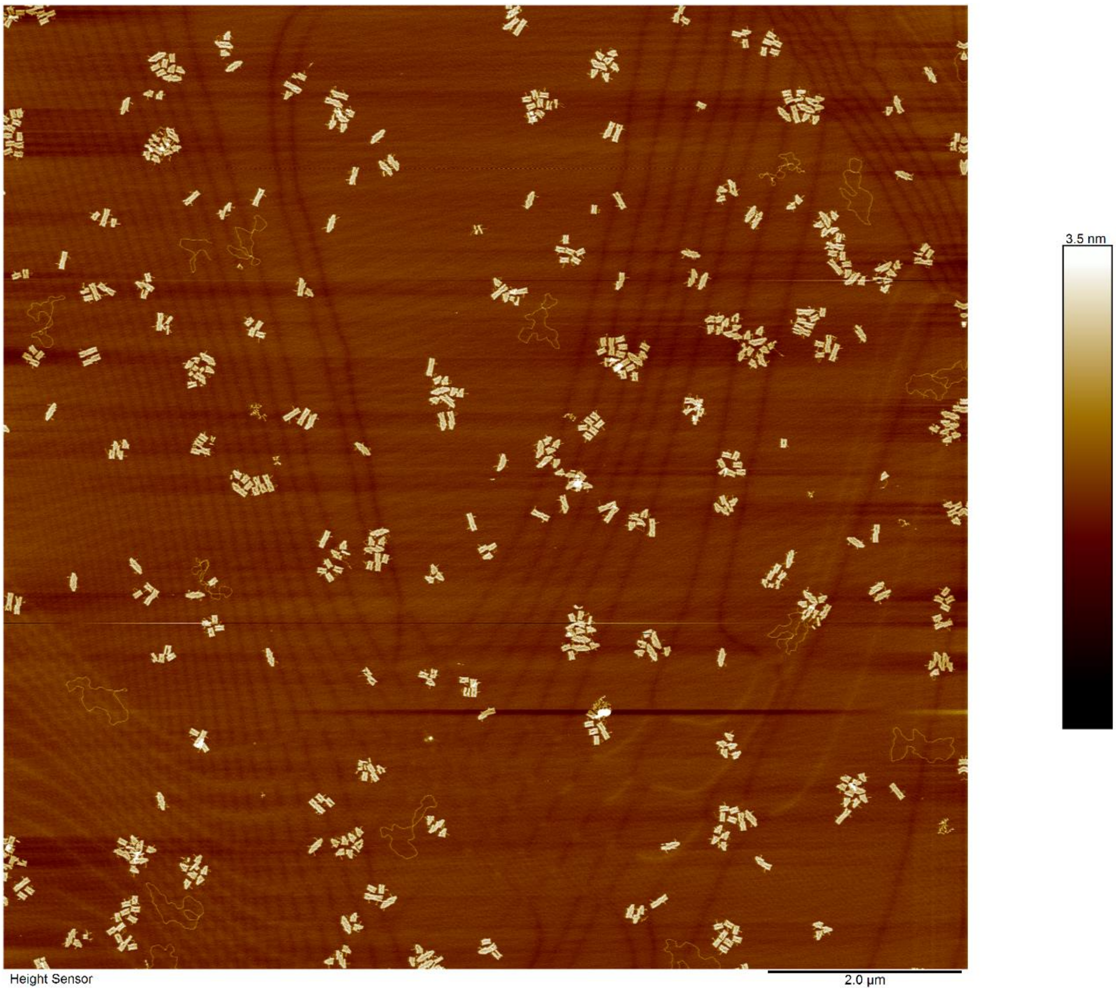

Figure S44. Truncated rectangle and rectangle origami tiles with integrated strands, each with 12× $(R_{F8})_2$.

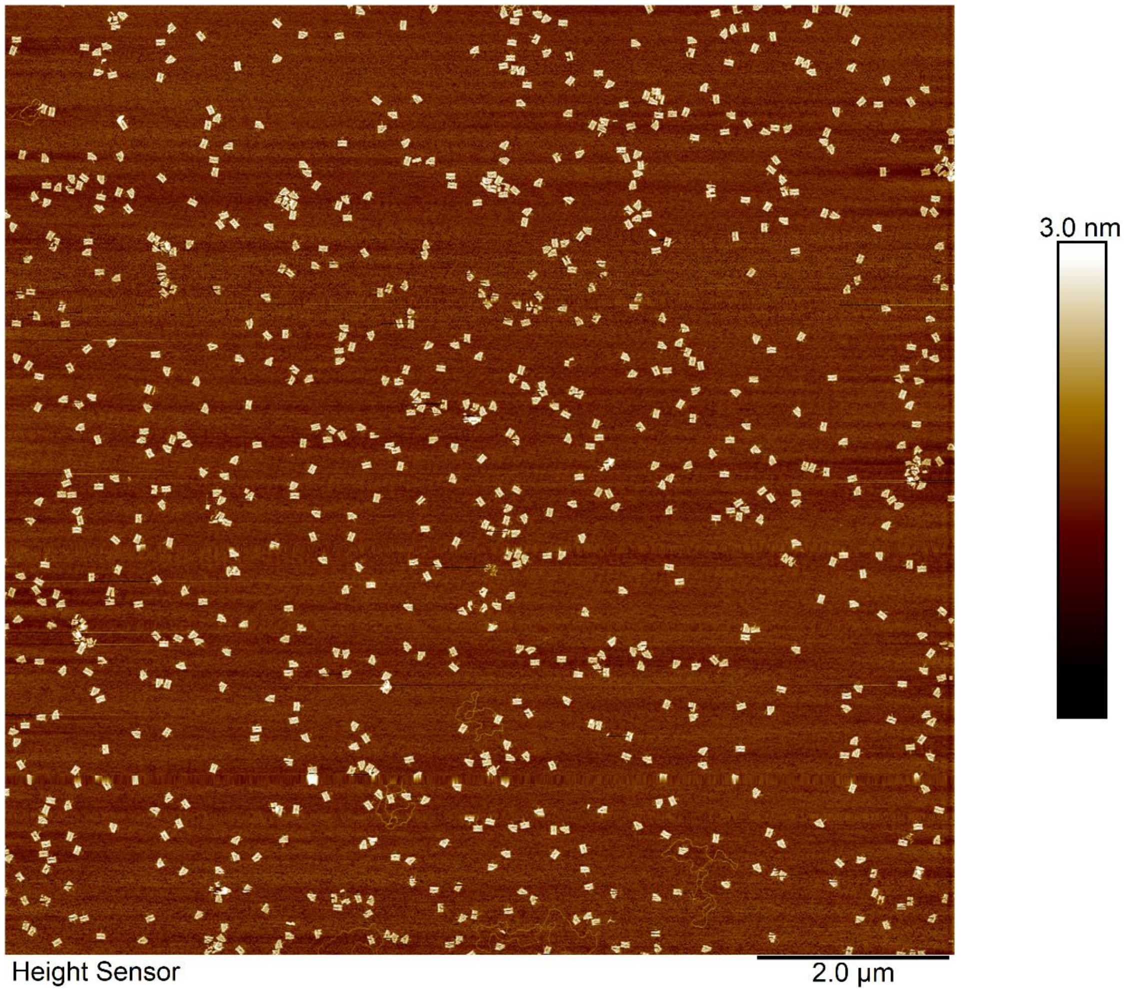

Figure S45. Truncated rectangle and rectangle origami tiles with integrated strands, each with no recognition tags.

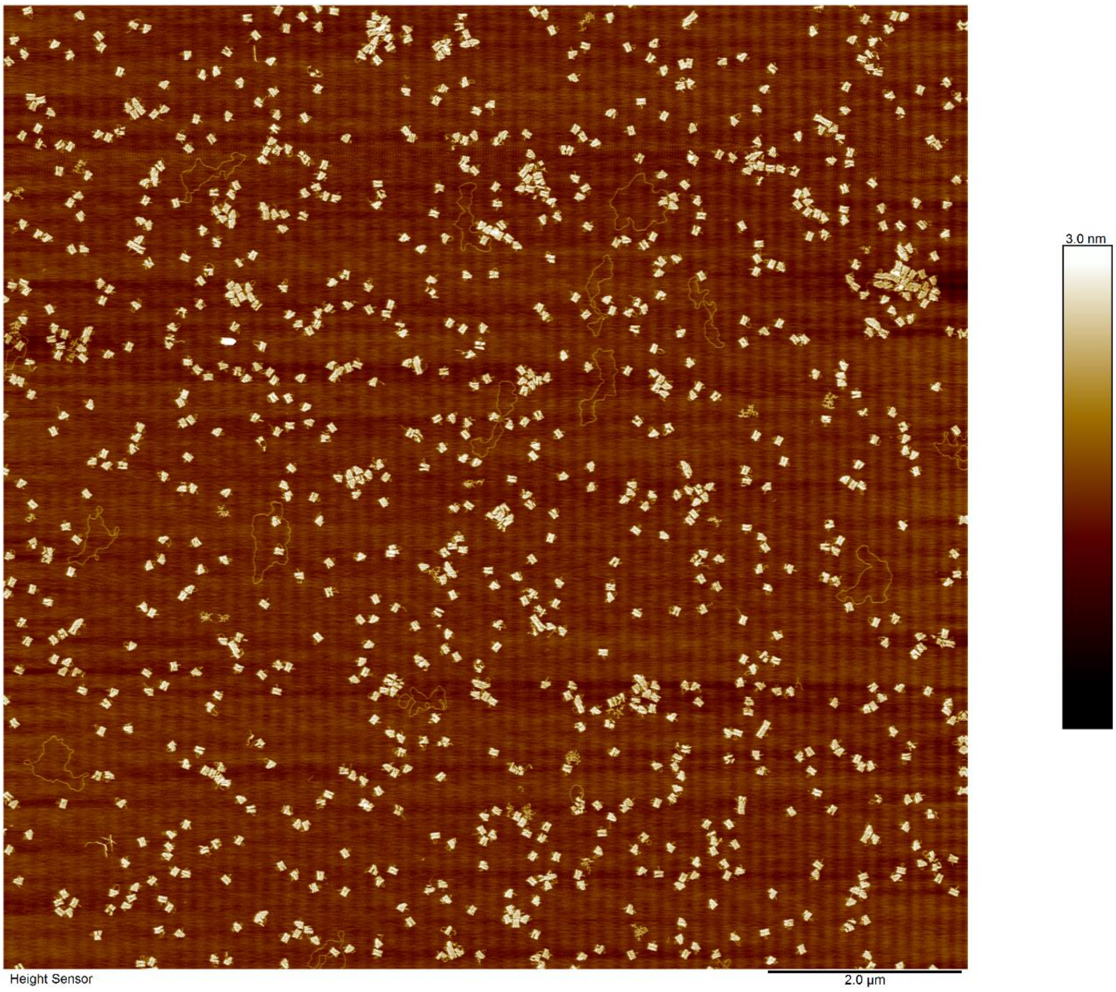

Figure S46. Truncated rectangle and rectangle origami tiles with integrated strands, each with 4× $(R_{F8})_2$ and 4× 5nt sticky ends.

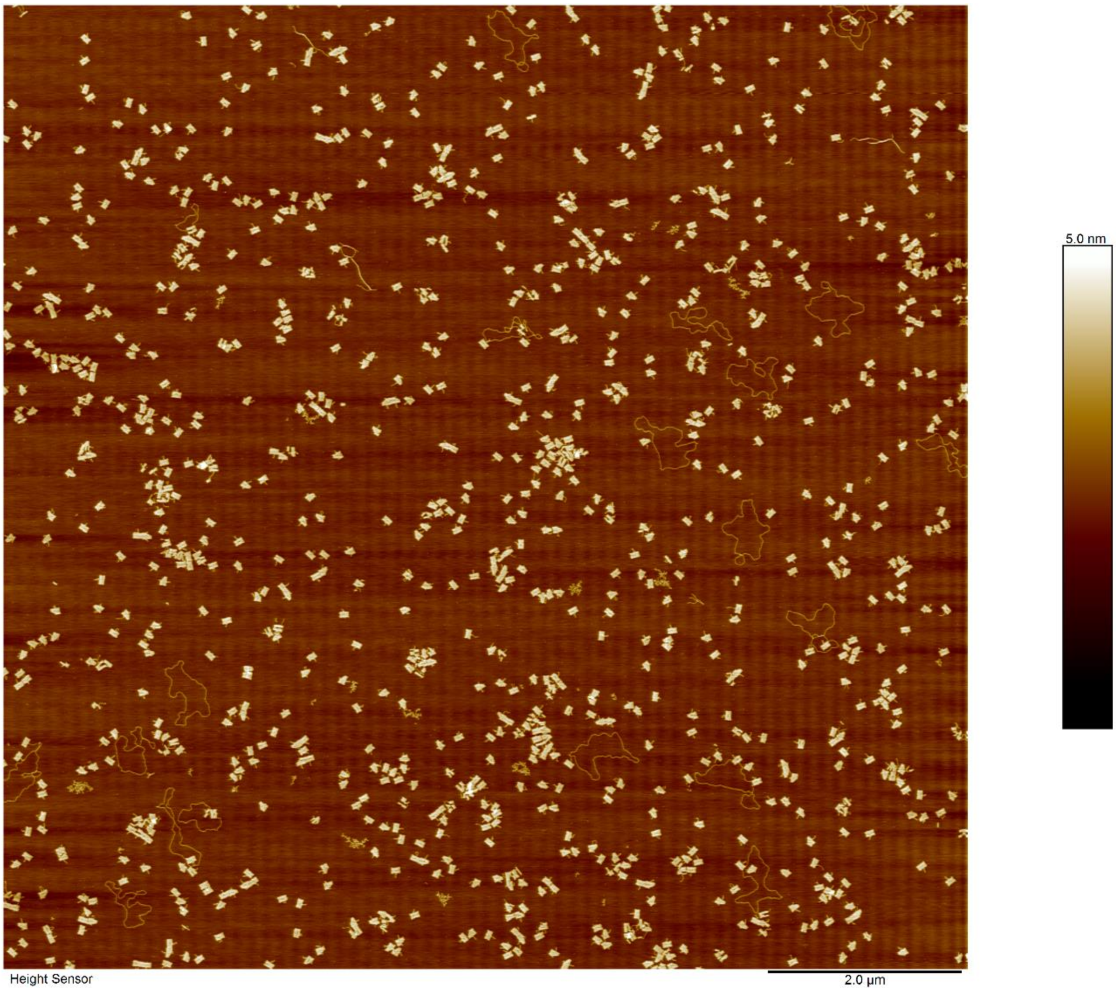

Figure S47. Truncated rectangle and rectangle origami tiles with integrated strands, each with 4× $(R_{F8})_2$ and 6× 5nt sticky ends.

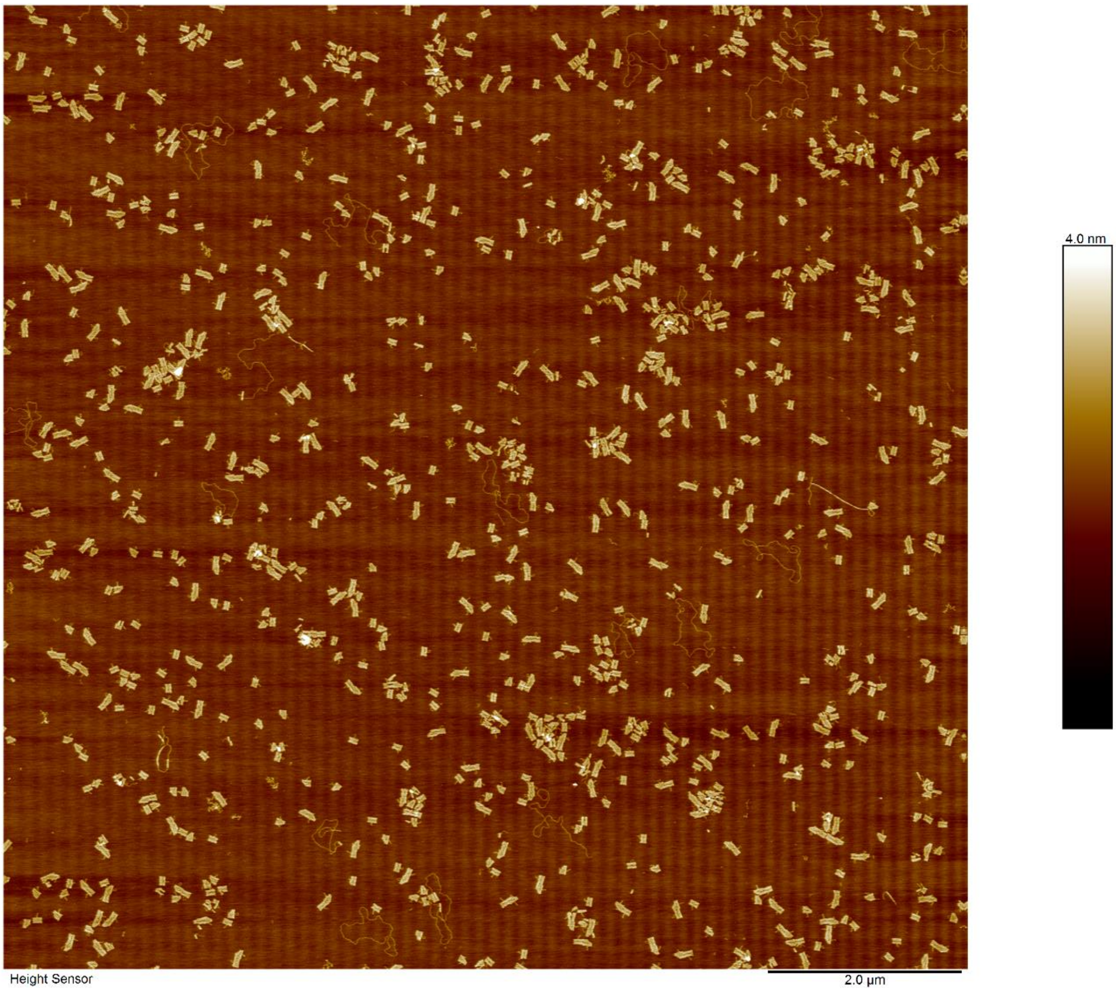

Figure S48. Truncated rectangle and rectangle origami tiles with integrated strands, each with 4× $(R_{F8})_2$ and 8× 5nt sticky ends.

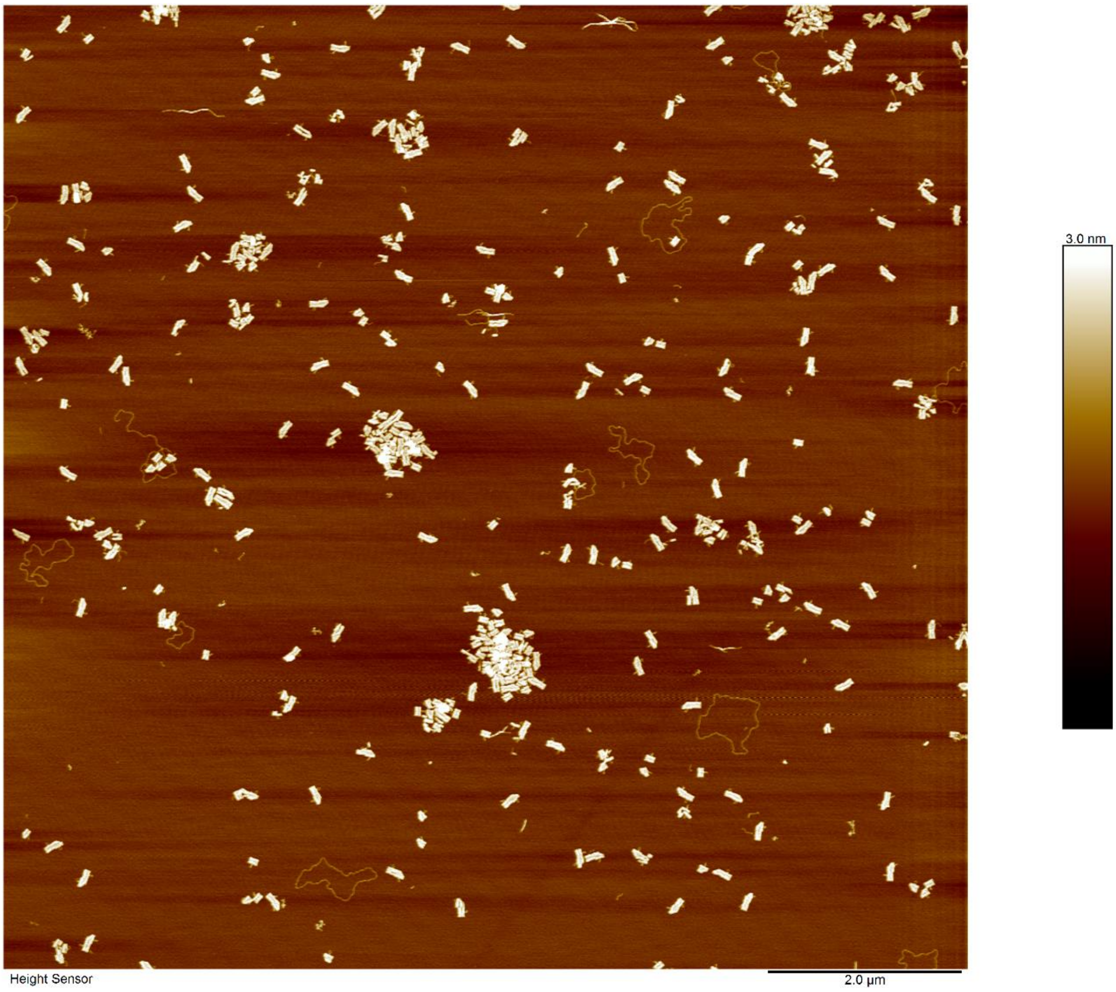

Figure S49. Truncated rectangle and rectangle origami tiles with integrated strands, each with 4× $(R_{F8})_2$ and 10× 5nt sticky ends.

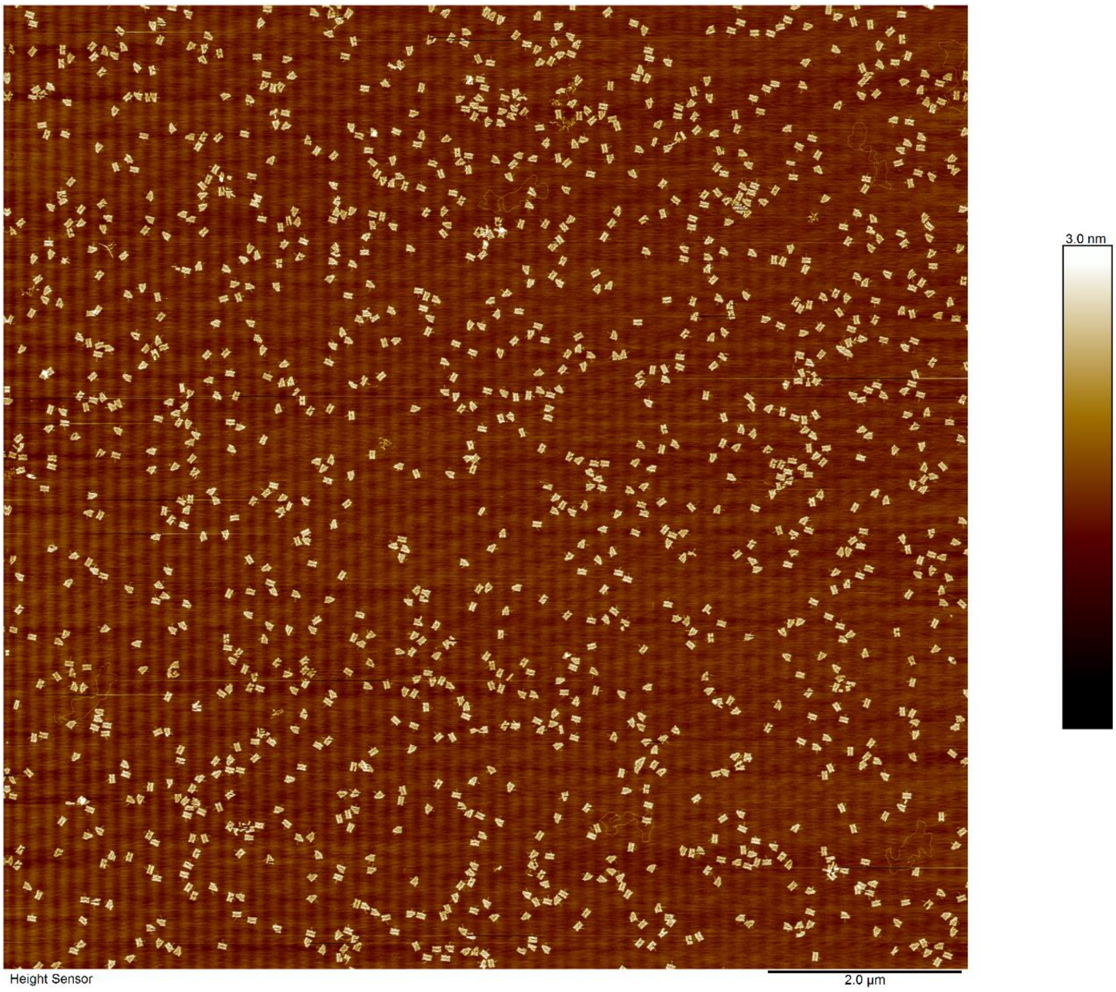

Figure S50. Truncated rectangle and rectangle origami tiles with integrated strands, each with 4× 5nt sticky ends (figure 5).

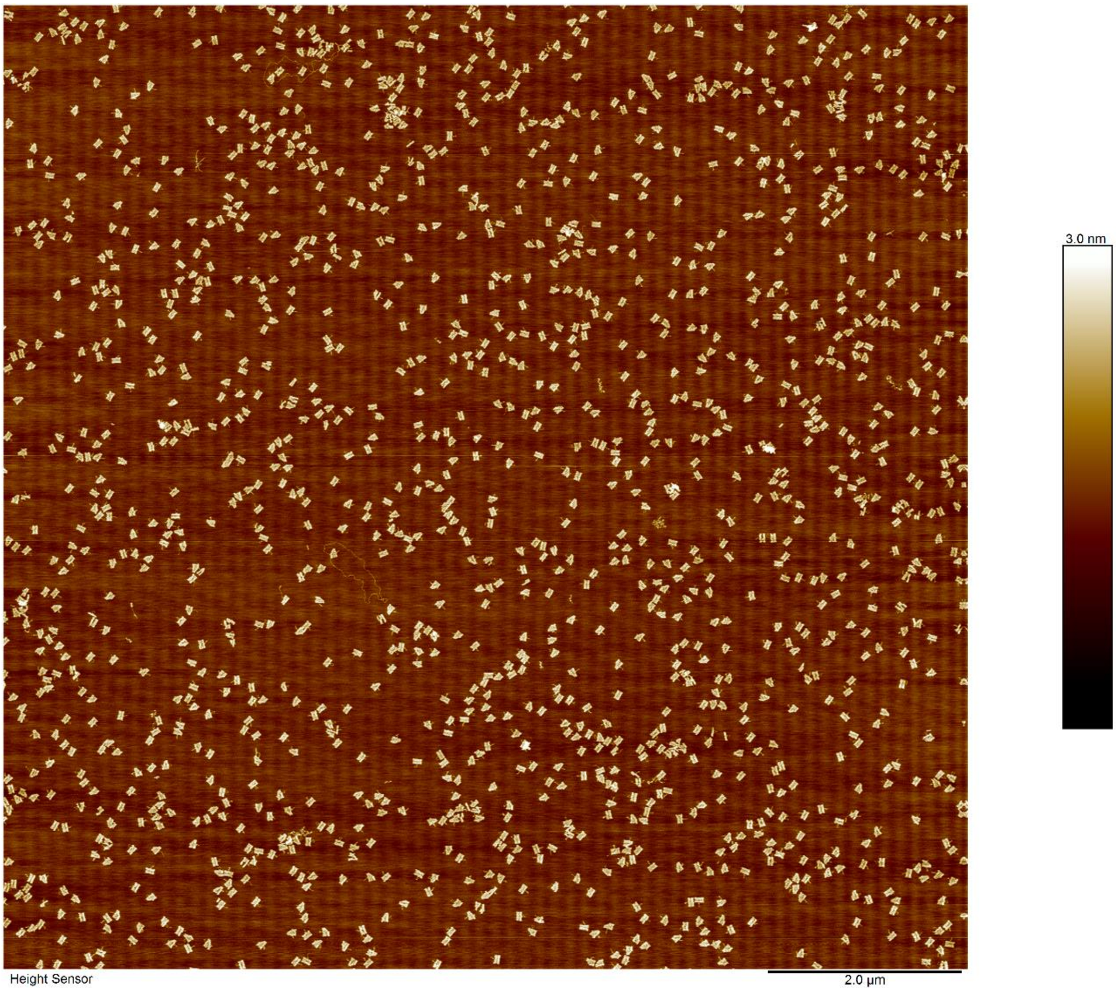

Figure S51. Truncated rectangle and rectangle origami tiles with integrated strands, each with 6× 5nt sticky ends (figure 5).

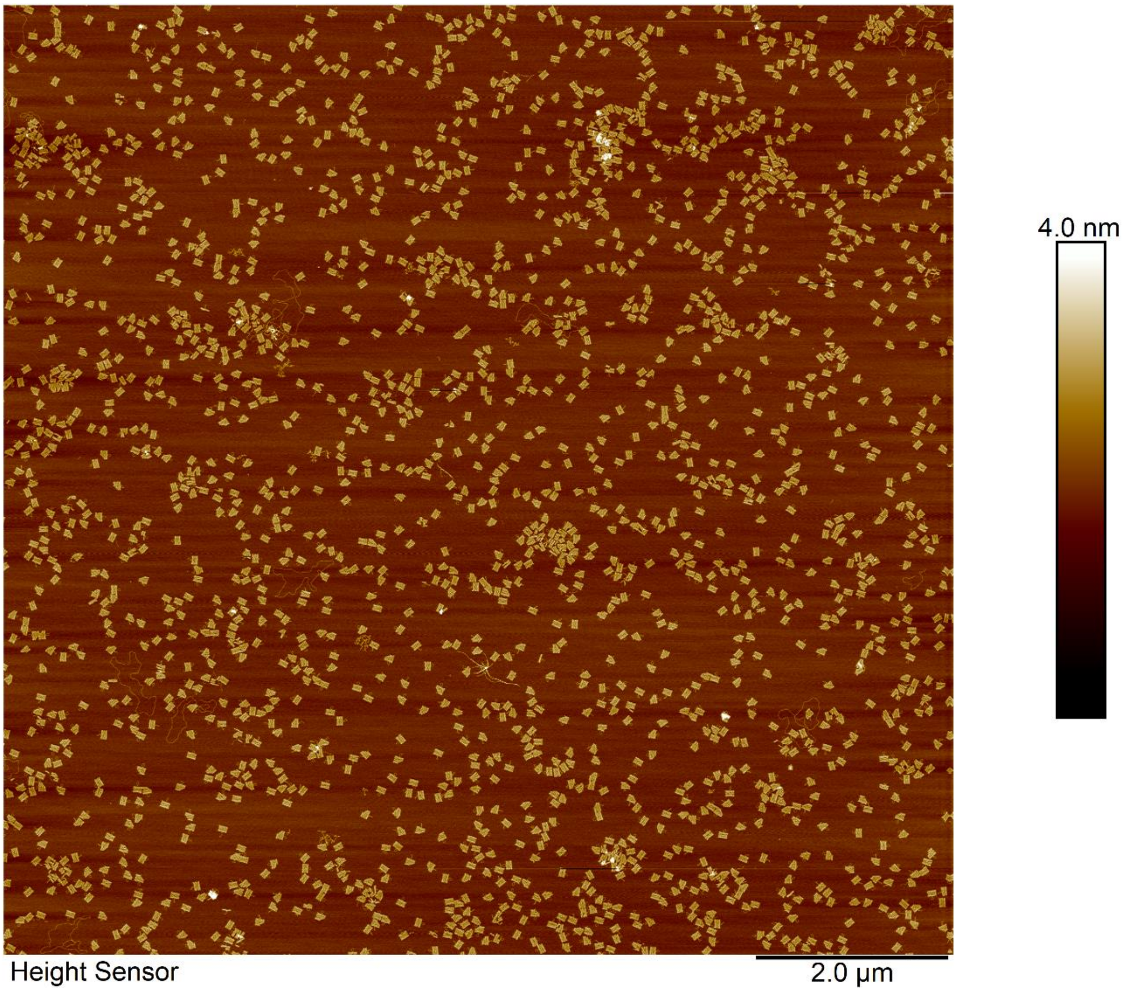

Figure S52. Truncated rectangle and rectangle origami tiles with integrated strands, each with 8× 5nt sticky ends (figure 5).

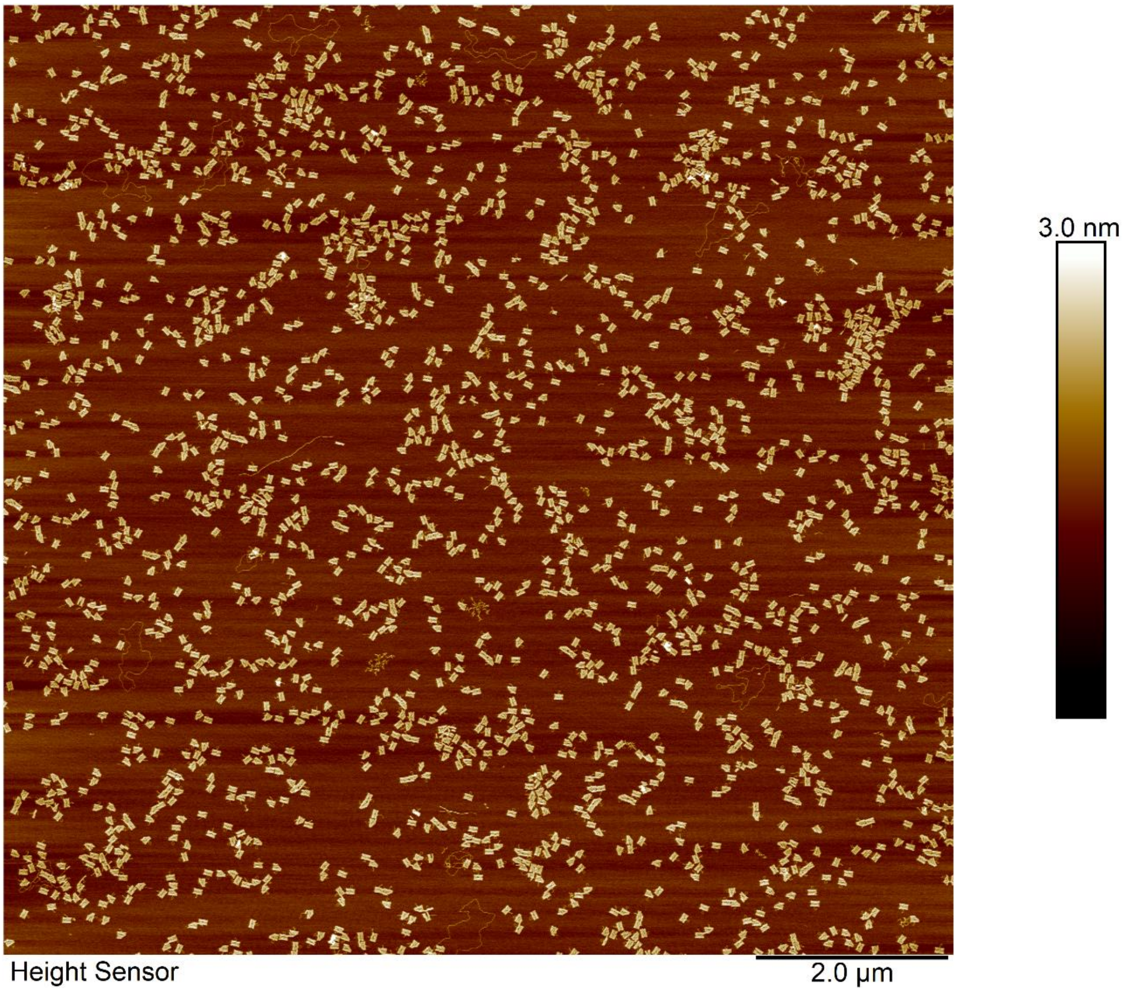

Figure S53. Truncated rectangle and rectangle origami tiles with integrated strands, each with 10× 5nt sticky ends (figure 5).

HPLC of Modified ODNs

Sample Name : ATS100A
Sample ID : ATS100A
Data Filename : origami staples21032022_ATS100A_02.lcd
Method Filename : GSH_Assay_Biozen_in_progress.lcm
Batch Filename : origami staples21032022.lcb

Vial # : 1-2
Injection Volume : 10 uL
Date Acquired : 21/03/2022 19:30:54
Date Processed : 21/03/2022 19:45:26

Sample Type : Unknown
Acquired by : Shimadzu
Processed by : Shimadzu

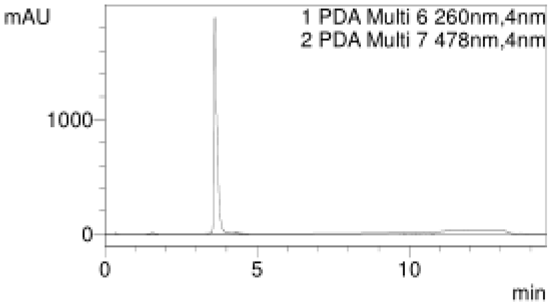

PDA Ch6 260nm

Peak#	Ret. Time	Area	Height	ID#
1	1.400	3259	970	
2	1.466	5614	2550	
3	1.578	39781	10741	
4	1.688	7067	1721	
5	3.613	13558968	1892426	
6	4.324	38279	6157	
Total		13652968	1914565	

PDA Ch7 478nm

Peak#	Ret. Time	Area	Height	ID#
Total				

PDA

Figure S54. Analytical HPLC trace of Int_ R_{F8}_A

Sample Name : ATS101B
Sample ID : ATS101B
Data Filename : origami staples21032022_ATS101B_03.lcd
Method Filename : GSH_Assay_Biozen_in_progress.lcm
Batch Filename : origami staples21032022.lcb
Vial # : 1-3
Injection Volume : 10 uL
Date Acquired : 21/03/2022 19:45:54
Date Processed : 21/03/2022 20:00:26

Sample Type : Unknown
Acquired by : Shimadzu
Processed by : Shimadzu

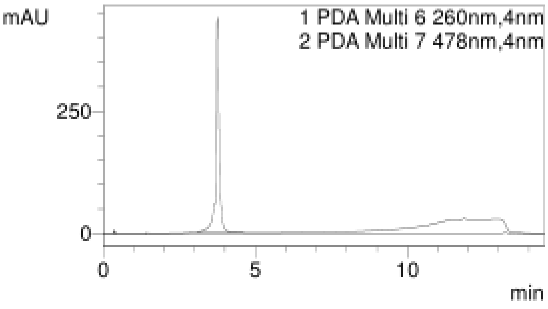

PDA Ch6 260nm

Peak#	Ret. Time	Area	Height	ID#
1	1.415	1690	858	
2	3.754	3288944	435092	
3	4.323	14558	1756	
Total		3305192	437707	

PDA Ch7 478nm

Peak#	Ret. Time	Area	Height	ID#
Total				

PDA

Figure S55. Analytical HPLC trace of Int_ R_{F8}_B

Sample Name : ATS102C
Sample ID : ATS102C
Data Filename : origami staples21032022_ATS102C_04.lcd
Method Filename : GSH_Assay_Biozen_in_progress.lcm
Batch Filename : origami staples21032022.lcb
Vial # : 1-4 Sample Type : Unknown
Injection Volume : 10 uL
Date Acquired : 21/03/2022 20:00:53 Acquired by : Shimadzu
Date Processed : 21/03/2022 20:15:26 Processed by : Shimadzu

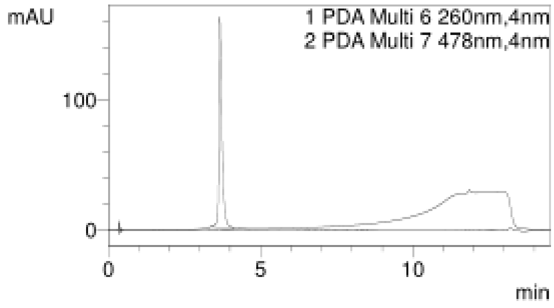

PDA Ch6 260nm

Peak#	Ret. Time	Area	Height	ID#
1	3.666	1089205	160153	
Total		1089205	160153	

PDA Ch7 478nm

Peak#	Ret. Time	Area	Height	ID#
Total				

PDA

Figure S56. Analytical HPLC trace of Int_ R_{F8}_C

Sample Name : ATS103D
Sample ID : ATS103D
Data Filename : origami staples21032022_ATS103D_05.lcd
Method Filename : GSH_Assay_Biozen_in_progress.lcm
Batch Filename : origami staples21032022.lcb
Vial # : 1-5 Sample Type : Unknown
Injection Volume : 10 uL

Date Acquired : 21/03/2022 20:15:53 Acquired by : Shimadzu
Date Processed : 21/03/2022 20:30:25 Processed by : Shimadzu

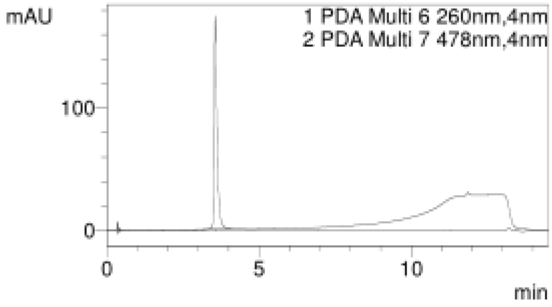

PDA Ch6 260nm

Peak#	Ret. Time	Area	Height	ID#
1	3.561	1111747	171936	
Total		1111747	171936	

PDA Ch7 478nm

Peak#	Ret. Time	Area	Height	ID#
Total				

PDA

Figure S57. Analytical HPLC trace of Int_ R_{F8}_D

Sample Name : ATS104E
Sample ID : ATS104E
Data Filename : origami staples21032022_ATS104E_06.lcd
Method Filename : GSH_Assay_Biozen_in_progress.lcm
Batch Filename : origami staples21032022.lcb
Vial # : 1-6 Sample Type : Unknown
Injection Volume : 10 uL
Date Acquired : 21/03/2022 20:30:52 Acquired by : Shimadzu
Date Processed : 21/03/2022 20:45:24 Processed by : Shimadzu

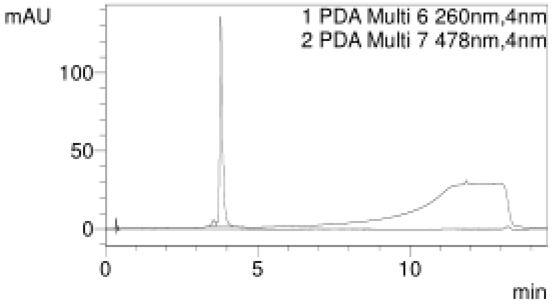

PDA Ch6 260nm

Peak#	Ret. Time	Area	Height	ID#
1	3.552	27958	3777	
2	3.792	870436	132830	
Total		898394	136608	

PDA Ch7 478nm

Peak#	Ret. Time	Area	Height	ID#
Total				

PDA

Figure S58. Analytical HPLC trace of Int_ R_{F8}_E

Sample Name : ATS105F
Sample ID : ATS105F
Data Filename : origami staples21032022_ATS105F_07.lcd
Method Filename : GSH_Assay_Biozen_in_progress.lcm
Batch Filename : origami staples21032022.lcb
Vial # : 1-7 Sample Type : Unknown
Injection Volume : 10 uL
Date Acquired : 21/03/2022 20:45:52 Acquired by : Shimadzu
Date Processed : 21/03/2022 21:00:24 Processed by : Shimadzu

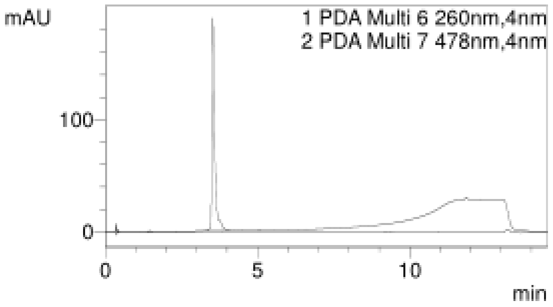

PDA Ch6 260nm

Peak#	Ret. Time	Area	Height	ID#
1	1.463	2585	645	
2	3.535	1206436	188103	
Total		1209021	188748	

PDA Ch7 478nm

Peak#	Ret. Time	Area	Height	ID#
Total				

PDA

Figure S59. Analytical HPLC trace of Int_ R_{F8}_F

Sample Name : ATS106G
Sample ID : ATS106G
Data Filename : origami staples21032022_ATS106G_08.lcd
Method Filename : GSH_Assay_Biozen_in_progress.lcm
Batch Filename : origami staples21032022.lcb
Vial # : 1-8 Sample Type : Unknown
Injection Volume : 10 uL
Date Acquired : 21/03/2022 21:00:52 Acquired by : Shimadzu

Date Processed : 21/03/2022 21:15:24 Processed by : Shimadzu

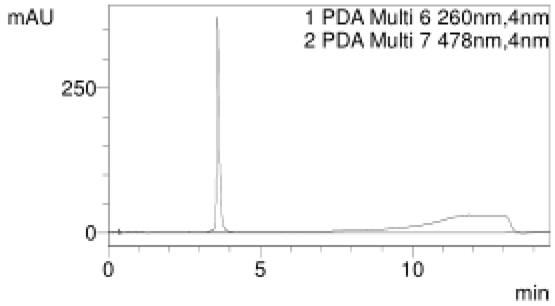

PDA Ch6 260nm

Peak#	Ret. Time	Area	Height	ID#
1	3.598	2285279	369458	
Total		2285279	369458	

PDA Ch7 478nm

Peak#	Ret. Time	Area	Height	ID#
Total				

PDA

Figure S60. Analytical HPLC trace of Int_ R_{F8}_G

Sample Name : ATS107H
Sample ID : ATS107H
Data Filename : origami staples21032022_ATS107H_09.lcd
Method Filename : GSH_Assay_Biozen_in_progress.lcm
Batch Filename : origami staples21032022.lcb
Vial # : 1-9 Sample Type : Unknown
Injection Volume : 10 uL
Date Acquired : 21/03/2022 21:15:53 Acquired by : Shimadzu
Date Processed : 21/03/2022 21:30:25 Processed by : Shimadzu

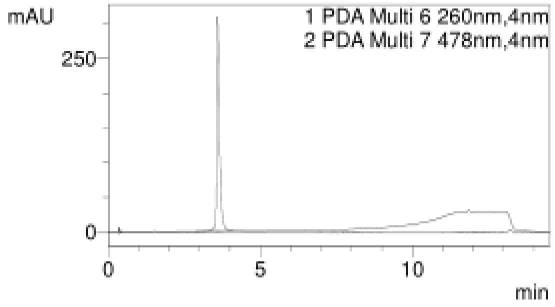

PDA Ch6 260nm

Peak#	Ret. Time	Area	Height	ID#
1	3.600	1932217	306388	
Total		1932217	306388	

PDA Ch7 478nm

Peak#	Ret. Time	Area	Height	ID#
Total				

PDA

Figure S61. Analytical HPLC trace of Int_ R_{F8}_H

Sample Name : ATS108I
Sample ID : ATS108I
Data Filename : origami staples21032022_ATS108I_10.lcd
Method Filename : GSH_Assay_Biozen_in_progress.lcm
Batch Filename : origami staples21032022.lcb
Vial # : 1-10 Sample Type : Unknown
Injection Volume : 10 uL
Date Acquired : 21/03/2022 21:30:54 Acquired by : Shimadzu
Date Processed : 21/03/2022 21:45:26 Processed by : Shimadzu

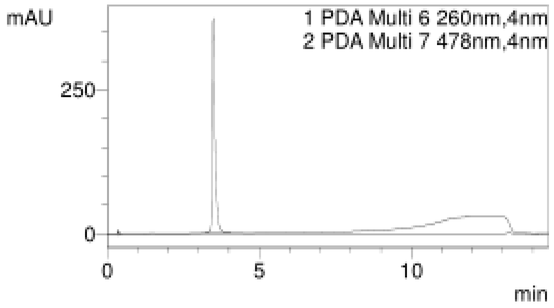

PDA Ch6 260nm

Peak#	Ret. Time	Area	Height	ID#
1	3.485	2325005	371295	
Total		2325005	371295	

PDA Ch7 478nm

Peak#	Ret. Time	Area	Height	ID#
Total				

PDA

Figure S62. Analytical HPLC trace of Int_ R_{F8}_I

Sample Name : ATS109J
Sample ID : ATS109J
Data Filename : origami staples21032022_ATS109J_11.lcd
Method Filename : GSH_Assay_Biozen_in_progress.lcm
Batch Filename : origami staples21032022.lcb
Vial # : 1-11   Sample Type : Unknown
Injection Volume : 10 uL
Date Acquired : 21/03/2022 21:45:55   Acquired by : Shimadzu

Date Processed : 21/03/2022 22:00:27   Processed by : Shimadzu

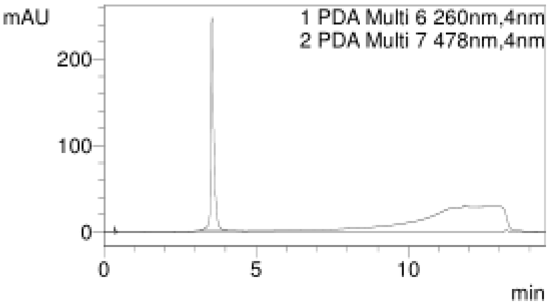

PDA Ch6 260nm

Peak#	Ret. Time	Area	Height	ID#
1	3.551	1657836	245437	
Total		1657836	245437	

PDA Ch7 478nm

Peak#	Ret. Time	Area	Height	ID#
Total				

PDA

Figure S63. Analytical HPLC trace of Int_ R_{F8}_J

Sample Name : ATS110K
Sample ID : ATS110K
Data Filename : origami staples21032022_ATS110K_12.lcd
Method Filename : GSH_Assay_Biozen_in_progress.lcm
Batch Filename : origami staples21032022.lcb
Vial # : 1-12   Sample Type : Unknown
Injection Volume : 10 uL
Date Acquired : 21/03/2022 22:00:55   Acquired by : Shimadzu
Date Processed : 21/03/2022 22:15:27   Processed by : Shimadzu

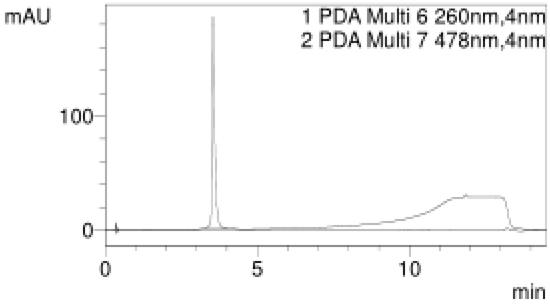

PDA Ch6 260nm

Peak#	Ret. Time	Area	Height	ID#
1	3.544	1131029	183368	
Total		1131029	183368	

PDA Ch7 478nm

Peak#	Ret. Time	Area	Height	ID#
Total				

PDA

Figure S64. Analytical HPLC trace of Int_ R_{F8}_K

Sample Name : ATS111L
Sample ID : ATS111L
Data Filename : origami staples21032022_ATS111L_13.lcd
Method Filename : GSH_Assay_Biozen_in_progress.lcm
Batch Filename : origami staples21032022.lcb
Vial # : 1-13　　Sample Type : Unknown
Injection Volume : 10 uL
Date Acquired : 21/03/2022 22:15:56　　Acquired by : Shimadzu
Date Processed : 21/03/2022 22:30:28　　Processed by : Shimadzu

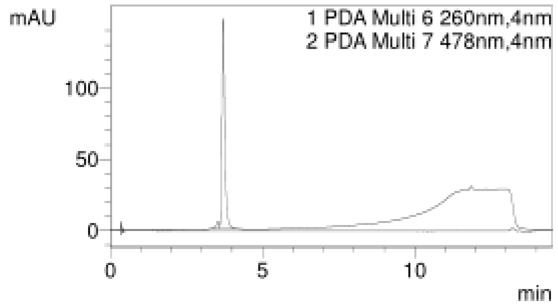

PDA Ch6 260nm

Peak#	Ret. Time	Area	Height	ID#
1	3.543	31447	4829	
2	3.706	879222	146034	
Total		910669	150863	

PDA Ch7 478nm

Peak#	Ret. Time	Area	Height	ID#
Total				

PDA

Figure S65. Analytical HPLC trace of Int_ R_{F8}_L

Sample Name : ATS112M
Sample ID : ATS112M
Data Filename : origami staples21032022_ATS112M_14.lcd
Method Filename : GSH_Assay_Biozen_in_progress.lcm
Batch Filename : origami staples21032022.lcb
Vial # : 1-14　　Sample Type : Unknown
Injection Volume : 10 uL
Date Acquired : 21/03/2022 22:30:57　　Acquired by : Shimadzu

Date Processed : 21/03/2022 22:45:29　　Processed by : Shimadzu

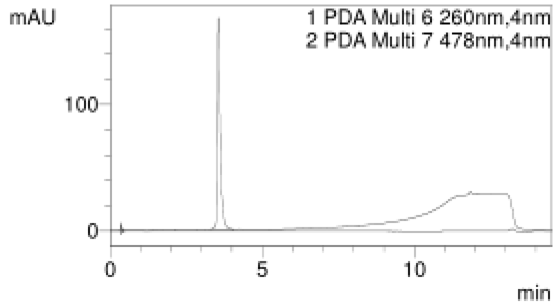

PDA Ch6 260nm

Peak#	Ret. Time	Area	Height	ID#
1	3.558	1104405	167234	
Total		1104405	167234	

PDA Ch7 478nm

Peak#	Ret. Time	Area	Height	ID#
Total				

PDA

Figure S66. Analytical HPLC trace of Int_ R_{F8}_M

Sample Name : ATS113N
Sample ID : ATS113N
Data Filename : origami staples21032022_ATS113N_15.lcd
Method Filename : GSH_Assay_Biozen_in_progress.lcm
Batch Filename : origami staples21032022.lcb
Vial # : 1-15
Sample Type : Unknown
Injection Volume : 10 uL
Date Acquired : 21/03/2022 22:45:58
Acquired by : Shimadzu
Date Processed : 21/03/2022 23:00:31
Processed by : Shimadzu

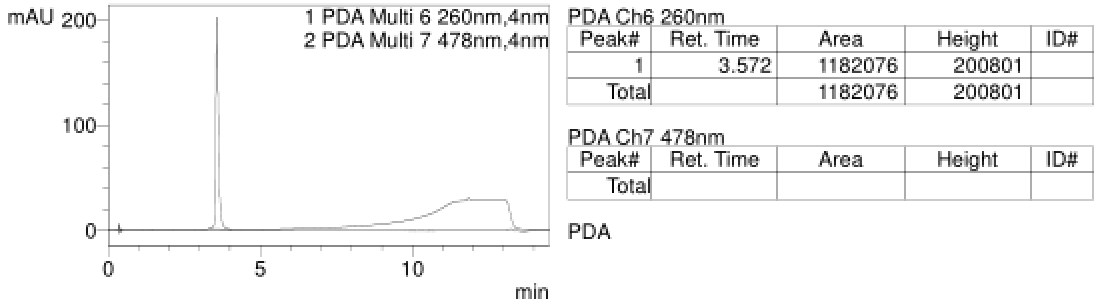

PDA Ch6 260nm

Peak#	Ret. Time	Area	Height	ID#
1	3.572	1182076	200801	
Total		1182076	200801	

PDA Ch7 478nm

Peak#	Ret. Time	Area	Height	ID#
Total				

Figure S67. Analytical HPLC trace of Int_R_{F8}_N

Sample Name : ATS114A
Sample ID : ATS114A
Data Filename : origami staples21032022_ATS114A_17.lcd
Method Filename : GSH_Assay_Biozen_in_progress.lcm
Batch Filename : origami staples21032022.lcb
Vial # : 1-16
Sample Type : Unknown
Injection Volume : 10 uL
Date Acquired : 21/03/2022 23:15:59
Acquired by : Shimadzu

Date Processed : 21/03/2022 23:30:31
Processed by : Shimadzu

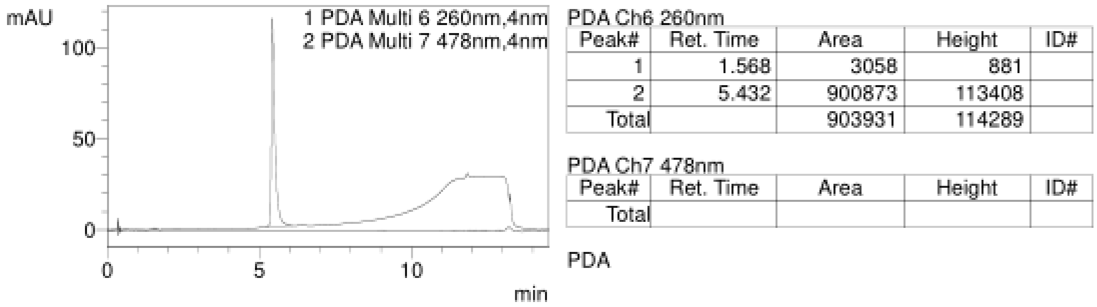

PDA Ch6 260nm

Peak#	Ret. Time	Area	Height	ID#
1	1.568	3058	881	
2	5.432	900873	113408	
Total		903931	114289	

PDA Ch7 478nm

Peak#	Ret. Time	Area	Height	ID#
Total				

Figure S68. Analytical HPLC trace of Int_$(R_{F8})_2$_A

Sample Name : ATS115B
Sample ID : ATS115B
Data Filename : origami staples21032022_ATS115B_18.lcd
Method Filename : GSH_Assay_Biozen_in_progress.lcm
Batch Filename : origami staples21032022.lcb
Vial # : 1-17
Injection Volume : 10 uL
Date Acquired : 21/03/2022 23:31:00
Date Processed : 21/03/2022 23:45:32

Sample Type : Unknown
Acquired by : Shimadzu
Processed by : Shimadzu

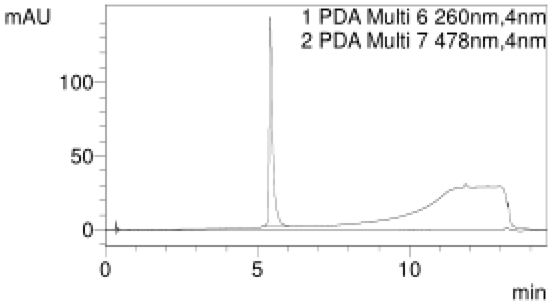

PDA Ch6 260nm

Peak#	Ret. Time	Area	Height	ID#
1	5.431	1091643	140678	
Total		1091643	140678	

PDA Ch7 478nm

Peak#	Ret. Time	Area	Height	ID#
Total				

PDA

Figure S69. Analytical HPLC trace of Int_(R_{F8})$_2$_B

Sample Name : ATS116C
Sample ID : ATS116C
Data Filename : origami staples21032022_ATS116C_19.lcd
Method Filename : GSH_Assay_Biozen_in_progress.lcm
Batch Filename : origami staples21032022.lcb
Vial # : 1-18
Injection Volume : 10 uL
Date Acquired : 21/03/2022 23:45:58
Date Processed : 22/03/2022 00:00:30

Sample Type : Unknown
Acquired by : Shimadzu
Processed by : Shimadzu

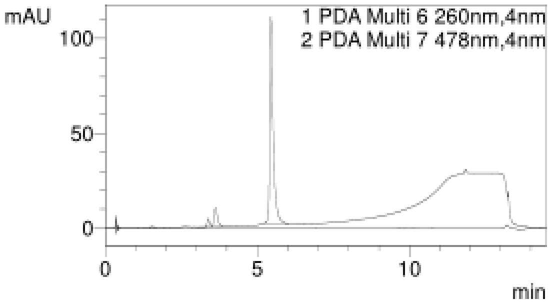

PDA Ch6 260nm

Peak#	Ret. Time	Area	Height	ID#
1	1.572	4563	793	
2	3.386	24534	4323	
3	3.625	74315	9588	
4	5.447	832337	108474	
Total		935748	123178	

PDA Ch7 478nm

Peak#	Ret. Time	Area	Height	ID#
Total				

PDA

Figure S70. Analytical HPLC trace of Int_(R_{F8})$_2$_C

Sample Name : ATS117D
Sample ID : ATS117D
Data Filename : origami staples21032022_ATS117D_20.lcd
Method Filename : GSH_Assay_Biozen_in_progress.lcm
Batch Filename : origami staples21032022.lcb
Vial # : 1-19
Injection Volume : 10 uL
Date Acquired : 22/03/2022 00:00:57
Date Processed : 22/03/2022 00:15:29

Sample Type : Unknown
Acquired by : Shimadzu
Processed by : Shimadzu

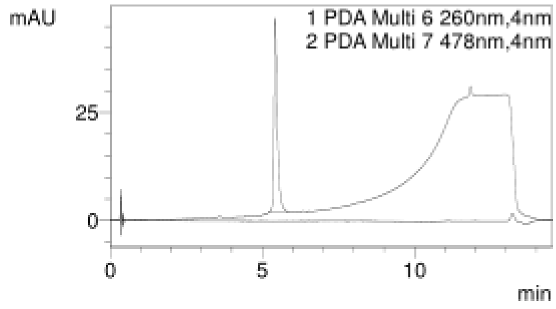

PDA Ch6 260nm

Peak#	Ret. Time	Area	Height	ID#
1	5.425	337839	44679	
Total		337839	44679	

PDA Ch7 478nm

Peak#	Ret. Time	Area	Height	ID#
Total				

PDA

Figure S71. Analytical HPLC trace of Int_(R_{F8})$_2$_D

Sample Name : ATS118E
Sample ID : ATS118E
Data Filename : origami staples21032022_ATS118E_21.lcd
Method Filename : GSH_Assay_Biozen_in_progress.lcm
Batch Filename : origami staples21032022.lcb
Vial # : 1-20
Sample Type : Unknown
Injection Volume : 10 uL
Date Acquired : 22/03/2022 00:15:56
Acquired by : Shimadzu
Date Processed : 22/03/2022 00:30:27
Processed by : Shimadzu

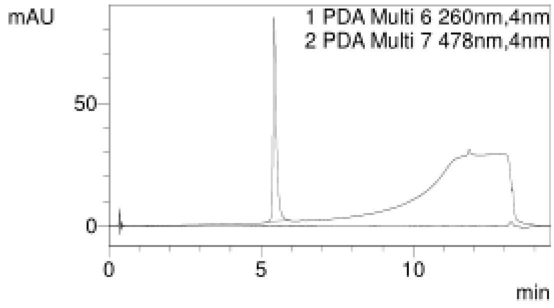

PDA Ch6 260nm

Peak#	Ret. Time	Area	Height	ID#
1	5.432	617254	82527	
Total		617254	82527	

PDA Ch7 478nm

Peak#	Ret. Time	Area	Height	ID#
Total				

PDA

Figure S72. Analytical HPLC trace of Int_(R_{F8})$_2$_E

Sample Name : ATS119F
Sample ID : ATS119F
Data Filename : origami staples21032022_ATS119F_22.lcd
Method Filename : GSH_Assay_Biozen_in_progress.lcm
Batch Filename : origami staples21032022.lcb
Vial # : 1-21
Sample Type : Unknown
Injection Volume : 10 uL
Date Acquired : 22/03/2022 00:30:55
Acquired by : Shimadzu
Date Processed : 22/03/2022 00:45:27
Processed by : Shimadzu

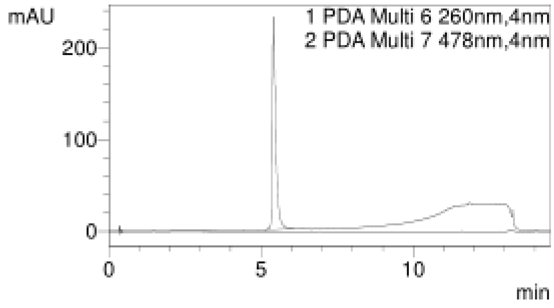

PDA Ch6 260nm

Peak#	Ret. Time	Area	Height	ID#
1	5.408	1783148	229824	
Total		1783148	229824	

PDA Ch7 478nm

Peak#	Ret. Time	Area	Height	ID#
Total				

PDA

Figure S73. Analytical HPLC trace of Int_(R_{F8})$_2$_F

Sample Name : ATS120G
Sample ID : ATS120G
Data Filename : origami staples21032022_ATS120G_23.lcd
Method Filename : GSH_Assay_Biozen_in_progress.lcm
Batch Filename : origami staples21032022.lcb
Vial # : 1-22 Sample Type : Unknown
Injection Volume : 10 uL
Date Acquired : 22/03/2022 00:45:54 Acquired by : Shimadzu
Date Processed : 22/03/2022 01:00:27 Processed by : Shimadzu

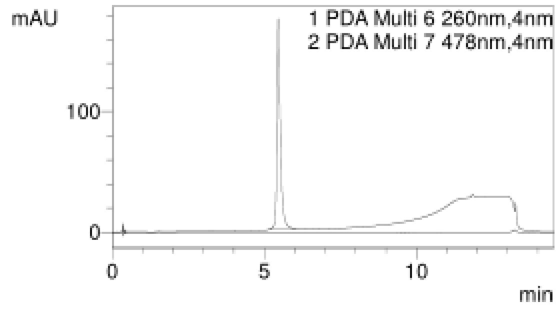

PDA Ch6 260nm

Peak#	Ret. Time	Area	Height	ID#
1	5.458	1357436	174035	
Total		1357436	174035	

PDA Ch7 478nm

Peak#	Ret. Time	Area	Height	ID#
Total				

PDA

Figure S74. Analytical HPLC trace of Int_$(R_{F8})_2$_G

Sample Name : ATS121H
Sample ID : ATS121H
Data Filename : origami staples21032022_ATS121H_24.lcd
Method Filename : GSH_Assay_Biozen_in_progress.lcm
Batch Filename : origami staples21032022.lcb
Vial # : 1-23 Sample Type : Unknown
Injection Volume : 10 uL
Date Acquired : 22/03/2022 01:00:54 Acquired by : Shimadzu
Date Processed : 22/03/2022 01:15:26 Processed by : Shimadzu

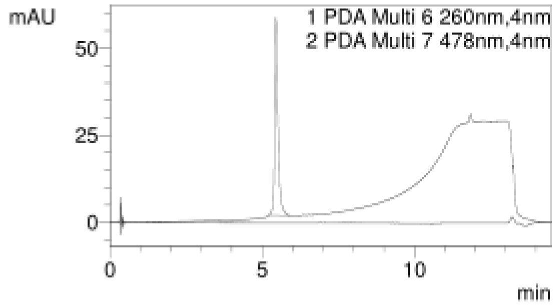

PDA Ch6 260nm

Peak#	Ret. Time	Area	Height	ID#
1	5.447	428342	56481	
Total		428342	56481	

PDA Ch7 478nm

Peak#	Ret. Time	Area	Height	ID#
Total				

PDA

Figure S75. Analytical HPLC trace of Int_$(R_{F8})_2$_H

Sample Name : ATS122I
Sample ID : ATS122I
Data Filename : origami staples21032022_ATS122I_25.lcd
Method Filename : GSH_Assay_Biozen_in_progress.lcm
Batch Filename : origami staples21032022.lcb
Vial # : 1-24 Sample Type : Unknown
Injection Volume : 10 uL
Date Acquired : 22/03/2022 01:15:55 Acquired by : Shimadzu
Date Processed : 22/03/2022 01:30:26 Processed by : Shimadzu

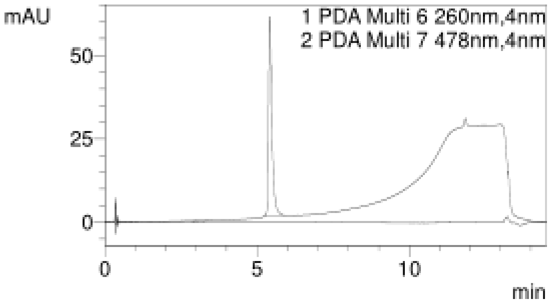

PDA Ch6 260nm

Peak#	Ret. Time	Area	Height	ID#
1	5.418	449716	59413	
Total		449716	59413	

PDA Ch7 478nm

Peak#	Ret. Time	Area	Height	ID#
Total				

PDA

Figure S76. Analytical HPLC trace of Int_$(R_{F8})_2$_I

Sample Name : ATS123J
Sample ID : ATS123J
Data Filename : origami staples21032022_ATS123J_26.lcd
Method Filename : GSH_Assay_Biozen_in_progress.lcm
Batch Filename : origami staples21032022.lcb
Vial # : 1-25 Sample Type : Unknown
Injection Volume : 10 uL
Date Acquired : 22/03/2022 01:30:54 Acquired by : Shimadzu
Date Processed : 22/03/2022 01:45:26 Processed by : Shimadzu

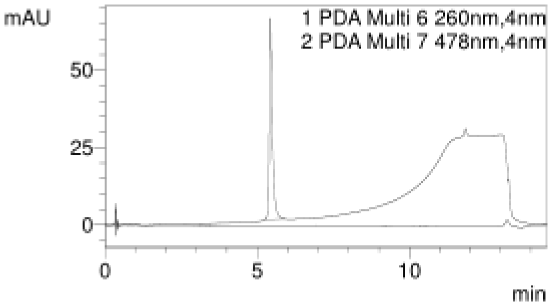

PDA Ch6 260nm

Peak#	Ret. Time	Area	Height	ID#
1	5.431	451380	64639	
Total		451380	64639	

PDA Ch7 478nm

Peak#	Ret. Time	Area	Height	ID#
Total				

PDA

Figure S77. Analytical HPLC trace of Int_$(R_{F8})_2$_J

Sample Name : ATS124K
Sample ID : ATS124K
Data Filename : origami staples21032022_ATS124K_27.lcd
Method Filename : GSH_Assay_Biozen_in_progress.lcm
Batch Filename : origami staples21032022.lcb
Vial # : 1-26
Sample Type : Unknown
Injection Volume : 10 uL
Date Acquired : 22/03/2022 01:45:55
Acquired by : Shimadzu
Date Processed : 22/03/2022 02:00:27
Processed by : Shimadzu

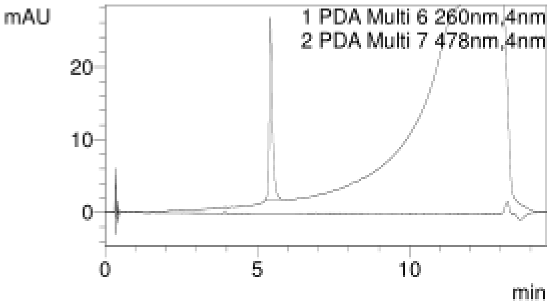

PDA Ch6 260nm

Peak#	Ret. Time	Area	Height	ID#
1	5.432	176121	24848	
Total		176121	24848	

PDA Ch7 478nm

Peak#	Ret. Time	Area	Height	ID#
Total				

PDA

Figure S78. Analytical HPLC trace of Int_$(R_{F8})_2$_K

Sample Name : ATS125L
Sample ID : ATS125L
Data Filename : origami staples21032022_ATS125L_28.lcd
Method Filename : GSH_Assay_Biozen_in_progress.lcm
Batch Filename : origami staples21032022.lcb
Vial # : 1-27
Sample Type : Unknown
Injection Volume : 10 uL
Date Acquired : 22/03/2022 02:00:55
Acquired by : Shimadzu
Date Processed : 22/03/2022 02:15:27
Processed by : Shimadzu

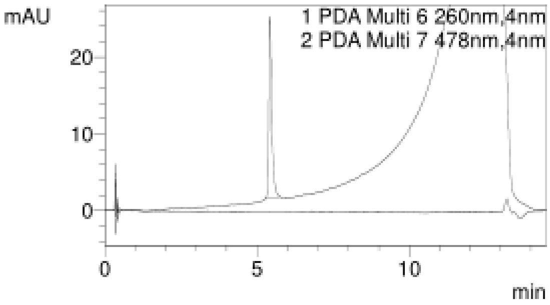

PDA Ch6 260nm

Peak#	Ret. Time	Area	Height	ID#
1	5.425	157290	23420	
Total		157290	23420	

PDA Ch7 478nm

Peak#	Ret. Time	Area	Height	ID#
Total				

PDA

Figure S79. Analytical HPLC trace of Int_$(R_{F8})_2$_L

Sample Name : ATS126M
Sample ID : ATS126M
Data Filename : origami staples21032022_ATS126M_29.lcd
Method Filename : GSH_Assay_Biozen_in_progress.lcm
Batch Filename : origami staples21032022.lcb
Vial # : 1-28
Sample Type : Unknown
Injection Volume : 10 uL
Date Acquired : 22/03/2022 02:15:56
Acquired by : Shimadzu
Date Processed : 22/03/2022 02:30:27
Processed by : Shimadzu

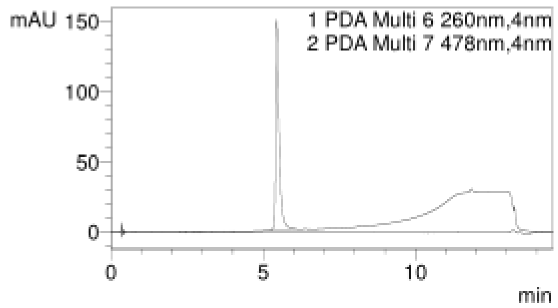

PDA Ch6 260nm

Peak#	Ret. Time	Area	Height	ID#
1	5.443	1172029	148887	
Total		1172029	148887	

PDA Ch7 478nm

Peak#	Ret. Time	Area	Height	ID#
Total				

PDA

Figure S80. Analytical HPLC trace of Int_(R_{F8})$_2$_M

Sample Name : ATS127N
Sample ID : ATS127N
Data Filename : origami staples21032022_ATS127N_30.lcd
Method Filename : GSH_Assay_Biozen_in_progress.lcm
Batch Filename : origami staples21032022.lcb
Vial # : 1-29 Sample Type : Unknown
Injection Volume : 10 uL
Date Acquired : 22/03/2022 02:30:55 Acquired by : Shimadzu
Date Processed : 22/03/2022 02:45:27 Processed by : Shimadzu

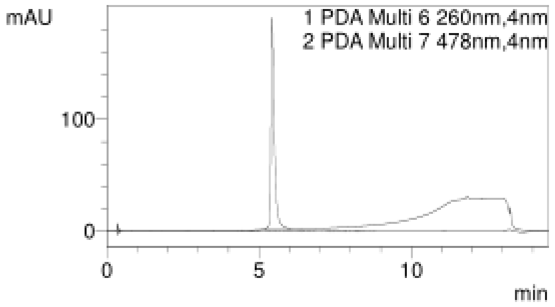

PDA Ch6 260nm

Peak#	Ret. Time	Area	Height	ID#
1	5.429	1432324	187856	
Total		1432324	187856	

PDA Ch7 478nm

Peak#	Ret. Time	Area	Height	ID#
Total				

PDA

Figure S81. Analytical HPLC trace of Int_(R_{F8})$_2$_N

Sample Name : ATS128A
Sample ID : ATS128A
Data Filename : origami staples21032022_ATS128A_32.lcd
Method Filename : GSH_Assay_Biozen_in_progress.lcm
Batch Filename : origami staples21032022.lcb
Vial # : 1-30 Sample Type : Unknown
Injection Volume : 10 uL
Date Acquired : 22/03/2022 03:00:55 Acquired by : Shimadzu
Date Processed : 22/03/2022 03:15:27 Processed by : Shimadzu

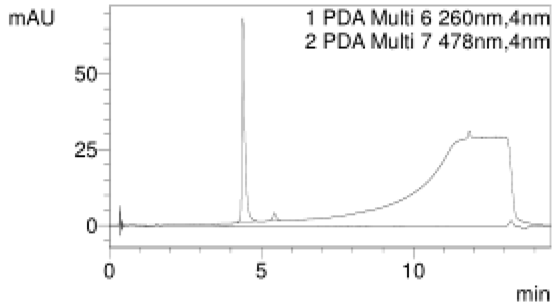

PDA Ch6 260nm

Peak#	Ret. Time	Area	Height	ID#
1	4.395	447477	67066	
2	5.429	16961	2433	
Total		464438	69499	

PDA Ch7 478nm

Peak#	Ret. Time	Area	Height	ID#
Total				

PDA

Figure S82. Analytical HPLC trace of Int_ALKYL_A

Sample Name : ATS129B
Sample ID : ATS129B
Data Filename : origami staples21032022_ATS129B_33.lcd
Method Filename : GSH_Assay_Biozen_in_progress.lcm
Batch Filename : origami staples21032022.lcb
Vial # : 1-31 Sample Type : Unknown
Injection Volume : 10 uL
Date Acquired : 22/03/2022 03:15:54 Acquired by : Shimadzu
Date Processed : 22/03/2022 03:30:26 Processed by : Shimadzu

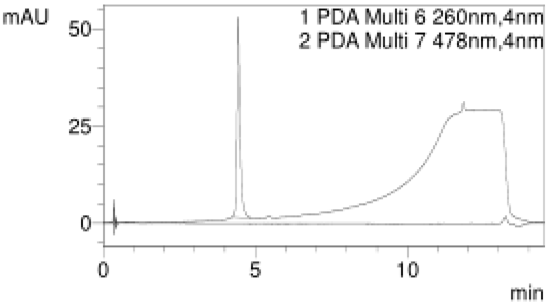

PDA Ch6 260nm

Peak#	Ret. Time	Area	Height	ID#
1	4.438	350489	51380	
Total		350489	51380	

PDA Ch7 478nm

Peak#	Ret. Time	Area	Height	ID#
Total				

PDA

Figure S83. Analytical HPLC trace of Int_ALKYL_B

Sample Name : ATS130C
Sample ID : ATS130C
Data Filename : origami staples21032022_ATS130C_34.lcd
Method Filename : GSH_Assay_Biozen_in_progress.lcm
Batch Filename : origami staples21032022.lcb
Vial # : 1-32 Sample Type : Unknown
Injection Volume : 10 uL
Date Acquired : 22/03/2022 03:30:54 Acquired by : Shimadzu
Date Processed : 22/03/2022 03:45:26 Processed by : Shimadzu

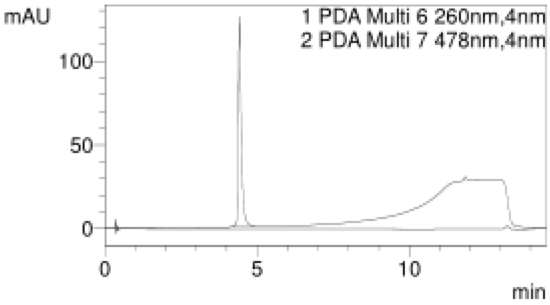

PDA Ch6 260nm

Peak#	Ret. Time	Area	Height	ID#
1	4.420	805626	124531	
Total		805626	124531	

PDA Ch7 478nm

Peak#	Ret. Time	Area	Height	ID#
Total				

PDA

Figure S84. Analytical HPLC trace of Int_ALKYL_C

Sample Name : ATS131D
Sample ID : ATS131D
Data Filename : origami staples21032022_ATS131D_35.lcd
Method Filename : GSH_Assay_Biozen_in_progress.lcm
Batch Filename : origami staples21032022.lcb
Vial # : 1-33 Sample Type : Unknown
Injection Volume : 10 uL
Date Acquired : 22/03/2022 03:45:54 Acquired by : Shimadzu
Date Processed : 22/03/2022 04:00:26 Processed by : Shimadzu

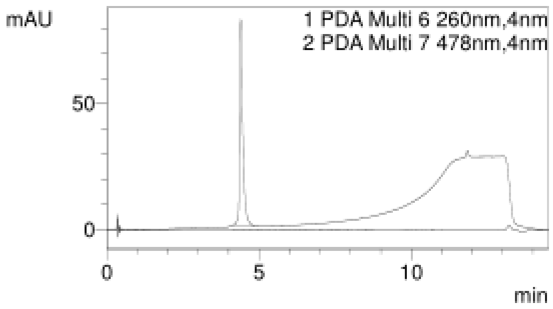

PDA Ch6 260nm

Peak#	Ret. Time	Area	Height	ID#
1	4.400	571437	81228	
Total		571437	81228	

PDA Ch7 478nm

Peak#	Ret. Time	Area	Height	ID#
Total				

PDA

Figure S85. Analytical HPLC trace of Int_ALKYL_D

Sample Name	: ATS132E		
Sample ID	: ATS132E		
Data Filename	: origami staples21032022_ATS132E_36.lcd		
Method Filename	: GSH_Assay_Biozen_in_progress.lcm		
Batch Filename	: origami staples21032022.lcb		
Vial #	: 1-34	Sample Type	: Unknown
Injection Volume	: 10 uL		
Date Acquired	: 22/03/2022 04:00:53	Acquired by	: Shimadzu
Date Processed	: 22/03/2022 04:15:25	Processed by	: Shimadzu

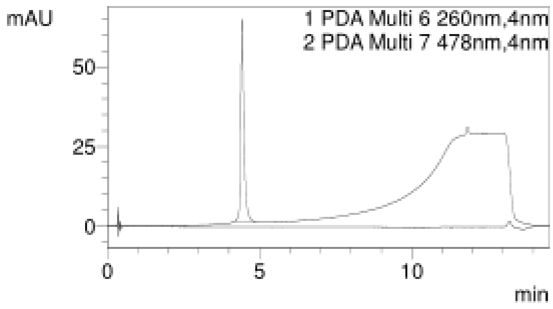

PDA Ch6 260nm

Peak#	Ret. Time	Area	Height	ID#
1	4.426	443029	62918	
Total		443029	62918	

PDA Ch7 478nm

Peak#	Ret. Time	Area	Height	ID#
Total				

PDA

Figure S86. Analytical HPLC trace of Int_ALKYL_E

Sample Name	: ATS133F		
Sample ID	: ATS133F		
Data Filename	: origami staples21032022_ATS133F_37.lcd		
Method Filename	: GSH_Assay_Biozen_in_progress.lcm		
Batch Filename	: origami staples21032022.lcb		
Vial #	: 1-35	Sample Type	: Unknown
Injection Volume	: 10 uL		
Date Acquired	: 22/03/2022 04:15:52	Acquired by	: Shimadzu
Date Processed	: 22/03/2022 04:30:23	Processed by	: Shimadzu

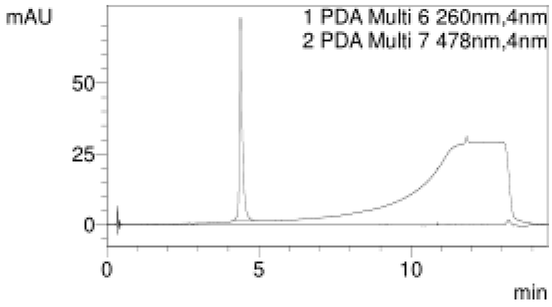

PDA Ch6 260nm

Peak#	Ret. Time	Area	Height	ID#
1	4.398	489028	71467	
Total		489028	71467	

PDA Ch7 478nm

Peak#	Ret. Time	Area	Height	ID#
Total				

PDA

Figure S87. Analytical HPLC trace of Int_ALKYL_F

Sample Name : ATS134G
Sample ID : ATS134G
Data Filename : origami staples21032022_ATS134G_38.lcd
Method Filename : GSH_Assay_Biozen_in_progress.lcm
Batch Filename : origami staples21032022.lcb
Vial # : 1-36 Sample Type : Unknown
Injection Volume : 10 uL
Date Acquired : 22/03/2022 04:30:51 Acquired by : Shimadzu
Date Processed : 22/03/2022 04:45:23 Processed by : Shimadzu

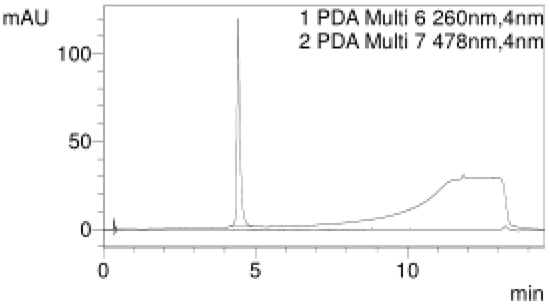

PDA Ch6 260nm

Peak#	Ret. Time	Area	Height	ID#
1	4.433	796434	117623	
Total		796434	117623	

PDA Ch7 478nm

Peak#	Ret. Time	Area	Height	ID#
Total				

PDA

Figure S88. Analytical HPLC trace of Int_ALKYL_G

Sample Name : ATS135H
Sample ID : ATS135H
Data Filename : origami staples21032022_ATS135H_39.lcd
Method Filename : GSH_Assay_Biozen_in_progress.lcm
Batch Filename : origami staples21032022.lcb
Vial # : 1-37 Sample Type : Unknown
Injection Volume : 10 uL
Date Acquired : 22/03/2022 04:45:50 Acquired by : Shimadzu
Date Processed : 22/03/2022 05:00:22 Processed by : Shimadzu

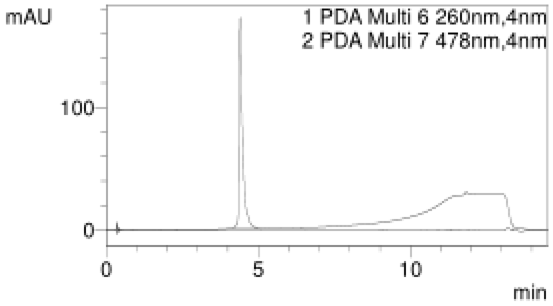

PDA Ch6 260nm

Peak#	Ret. Time	Area	Height	ID#
1	4.406	1290865	171033	
Total		1290865	171033	

PDA Ch7 478nm

Peak#	Ret. Time	Area	Height	ID#
Total				

PDA

Figure S89. Analytical HPLC trace of Int_ALKYL_H

Sample Name : ATS136I
Sample ID : ATS136I
Data Filename : origami staples21032022_ATS136I_40.lcd
Method Filename : GSH_Assay_Biozen_in_progress.lcm
Batch Filename : origami staples21032022.lcb
Vial # : 1-38 Sample Type : Unknown
Injection Volume : 10 uL
Date Acquired : 22/03/2022 05:00:49 Acquired by : Shimadzu
Date Processed : 22/03/2022 05:15:21 Processed by : Shimadzu

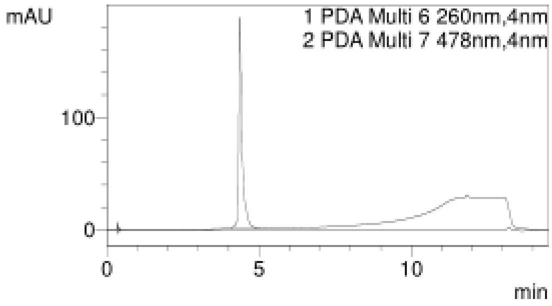

PDA Ch6 260nm

Peak#	Ret. Time	Area	Height	ID#
1	4.371	1374613	186720	
Total		1374613	186720	

PDA Ch7 478nm

Peak#	Ret. Time	Area	Height	ID#
Total				

PDA

Figure S90. Analytical HPLC trace of Int_ALKYL_I

Sample Name : ATS137J
Sample ID : ATS137J
Data Filename : origami staples21032022_ATS137J_41.lcd
Method Filename : GSH_Assay_Biozen_in_progress.lcm
Batch Filename : origami staples21032022.lcb
Vial # : 1-39 Sample Type : Unknown
Injection Volume : 10 uL
Date Acquired : 22/03/2022 05:15:49 Acquired by : Shimadzu
Date Processed : 22/03/2022 05:30:21 Processed by : Shimadzu

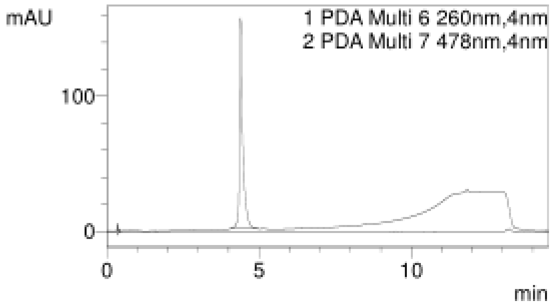

PDA Ch6 260nm

Peak#	Ret. Time	Area	Height	ID#
1	4.396	1184261	155694	
Total		1184261	155694	

PDA Ch7 478nm

Peak#	Ret. Time	Area	Height	ID#
Total				

PDA

Figure S91. Analytical HPLC trace of Int_ALKYL_J

Sample Name : ATS138K
Sample ID : ATS138K
Data Filename : origami staples21032022_ATS138K_42.lcd
Method Filename : GSH_Assay_Biozen_in_progress.lcm
Batch Filename : origami staples21032022.lcb
Vial # : 1-40
Sample Type : Unknown
Injection Volume : 10 uL
Date Acquired : 22/03/2022 05:30:51
Acquired by : Shimadzu
Date Processed : 22/03/2022 05:45:22
Processed by : Shimadzu

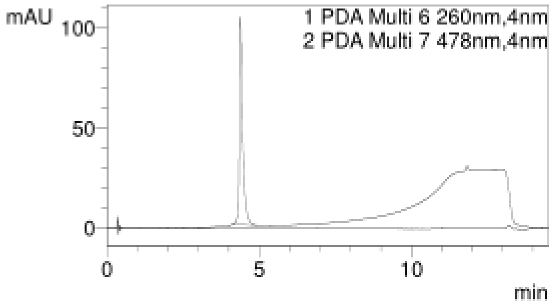

PDA Ch6 260nm

Peak#	Ret. Time	Area	Height	ID#
1	4.383	754735	102983	
Total		754735	102983	

PDA Ch7 478nm

Peak#	Ret. Time	Area	Height	ID#
Total				

PDA

Figure S92. Analytical HPLC trace of Int_ALKYL_K

Sample Name : ATS139L
Sample ID : ATS139L
Data Filename : origami staples21032022_ATS139L_43.lcd
Method Filename : GSH_Assay_Biozen_in_progress.lcm
Batch Filename : origami staples21032022.lcb
Vial # : 1-41
Sample Type : Unknown
Injection Volume : 10 uL
Date Acquired : 22/03/2022 05:45:51
Acquired by : Shimadzu
Date Processed : 22/03/2022 06:00:22
Processed by : Shimadzu

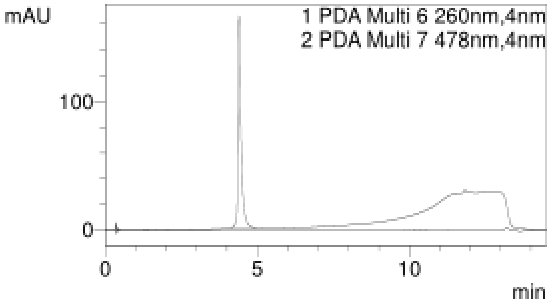

PDA Ch6 260nm

Peak#	Ret. Time	Area	Height	ID#
1	4.400	1143807	162501	
Total		1143807	162501	

PDA Ch7 478nm

Peak#	Ret. Time	Area	Height	ID#
Total				

PDA

Figure S93. Analytical HPLC trace of Int_ALKYL_L

Sample Name : ATS140M
Sample ID : ATS140M
Data Filename : origami staples21032022_ATS140M_44.lcd
Method Filename : GSH_Assay_Biozen_in_progress.lcm
Batch Filename : origami staples21032022.lcb
Vial # : 1-42
Sample Type : Unknown
Injection Volume : 10 uL
Date Acquired : 22/03/2022 06:00:51
Acquired by : Shimadzu
Date Processed : 22/03/2022 06:15:23
Processed by : Shimadzu

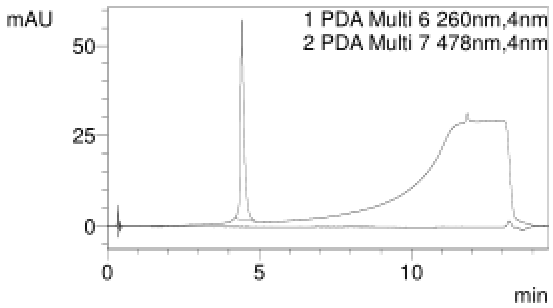

PDA Ch6 260nm

Peak#	Ret. Time	Area	Height	ID#
1	4.425	436320	54972	
Total		436320	54972	

PDA Ch7 478nm

Peak#	Ret. Time	Area	Height	ID#
Total				

PDA

Figure S94. Analytical HPLC trace of Int_ALKYL_M

Sample Name : ATS141N
Sample ID : ATS141N
Data Filename : origami staples21032022_ATS141N_45.lcd
Method Filename : GSH_Assay_Biozen_in_progress.lcm
Batch Filename : origami staples21032022.lcb
Vial # : 1-43
Sample Type : Unknown
Injection Volume : 10 uL
Date Acquired : 22/03/2022 06:15:51
Acquired by : Shimadzu
Date Processed : 22/03/2022 06:30:23
Processed by : Shimadzu

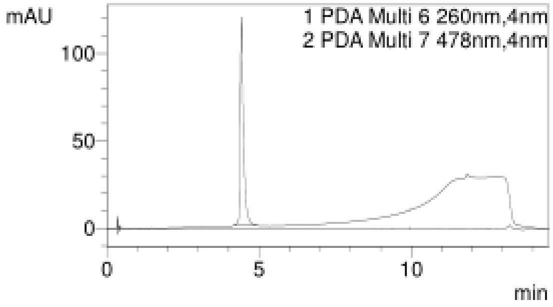

PDA Ch6 260nm

Peak#	Ret. Time	Area	Height	ID#
1	4.422	833470	118675	
Total		833470	118675	

PDA Ch7 478nm

Peak#	Ret. Time	Area	Height	ID#
Total				

PDA

Figure S95. Analytical HPLC trace of Int_ALKYL_N
